# Access to Unsaturated Organogermanes via (De)Hydrosilylation Mediated by Cobalt Complexes

**DOI:** 10.1021/acs.orglett.3c02326

**Published:** 2023-08-30

**Authors:** Konstancja Broniarz, Grzegorz Hreczycho

**Affiliations:** Faculty of Chemistry, Adam Mickiewicz University, Uniwersytetu Poznanskiego St. 8, 61-614 Poznan, Poland

## Abstract

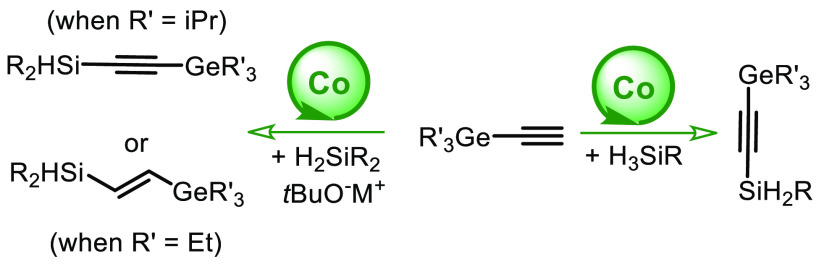

The functionalization of alkynylgermanes using hydrosilanes was accomplished by employing cobalt catalysis. Depending on the reactants used, the reaction can proceed via dehydrogenative coupling or hydrosilylation. Importantly, the presented method is characterized by mild reaction conditions, allowing rapid access to a wide range of organogermanes.

Organogermanium compounds have emerged as valuable building blocks for the construction of highly functionalized molecules^1^ and have hence attracted major attention in molecular synthesis, including cross-coupling reactions,^2^ germylation of amides,^3^ etc.^4^ This has led to increased demand and interest in developing new, efficient methods for constructing sp^2^ C–Ge and sp C–Ge bonds.

Alkynylgermanes can be synthesized via classical stoichiometric reactions between moisture-sensitive halogermanes and metal acetylides (Scheme 1, A).^5^ Beyond this approach, which is accompanied by reactive substrates and byproducts, several catalytic methods have been developed. These include expensive transition metal catalysis (Ru,^6a,6b^ Ir,^6c,6d^ etc.; Scheme 1, B) and Lewis acid-catalyzed procedures (Scheme 1, C).^6e^ Very recently, our group developed two independent protocols allowing for convenient access to alkynylgermanes in the presence of inexpensive potassium bis(trimethylsilyl)amide (KHMDS) (Scheme 1, D).^6f,6g^

**Scheme 1:**
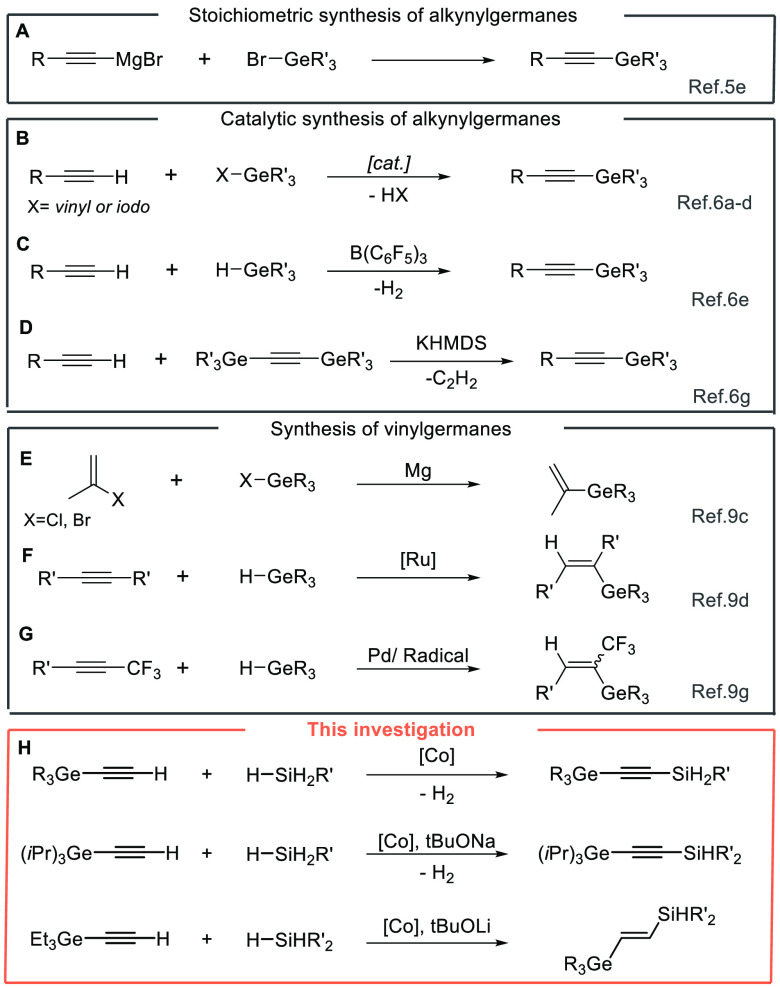
Context of the Investigation

Vinylgermanes also have proven to be useful reagents that can be converted to functional organic molecules with retention of the double-bond geometry.^7^ There are several conventional methods for the synthesis of vinylgermanes.^8^ Catalytic reactions are mainly based on the hydrogermylation of alkynes. Many examples of these reactions are catalyzed by transition metals, including metals like Ru,^9a,9b^ Pd,^9c−9g^ Rh,^9h^ and Pt^9i,9j^ (Scheme 1, E–G). There are also a few methods that proceed in the presence of environmentally friendly and cheaper Fe,^9k^ Mn,^9l^ and Co^9m^ catalysts.

Sustainable and less-toxic synthetic approaches that proceed with 3d metal catalysts have gained significant attention recently. A very promising group of catalysts that have attracted particular attention are pincer cobalt complexes, which are known due to their high stability, activity, and selectivity.^10a−10g^ What is also very interesting is that some cobalt complexes can also change the chemoselectivity or reaction direction depending on the process conditions.^10h−10m^

Recently, we have demonstrated the applicability of cobalt complexes in organosilicon and organoboron synthesis, and the developed methods often exhibited unique selectivity and chemoselectivity.^10n−10s^ Therefore, we decided to examine pincer cobalt complexes based on the triazine backbone (**A**, **B**, and **C**; Table S1 in the Supporting Information) in the functionalization of alkynylgermanes. As a result, we developed a new protocol enabling the synthesis of unsaturated germanium compounds, which are obtained by dehydrogenative coupling or hydrosilylation depending on the reaction conditions and the reactants used. To the best of our knowledge, this is an unprecedented example of a single catalytic system that allows for the synthesis of both vinyl- and alkynylgermane compounds.

First, we performed the optimization study of the catalytic sp C–H silylation of triethylgermylacetylene, which is summarized in Table 1 (please see also Table S1 in the Supporting Information for more details). It is worth noting that all tests were conducted using new Schlenk tubes and magnetic stirrers. This is highly important to eliminate the influence of traces of other transition metal impurities.^11^

**Table 1:**
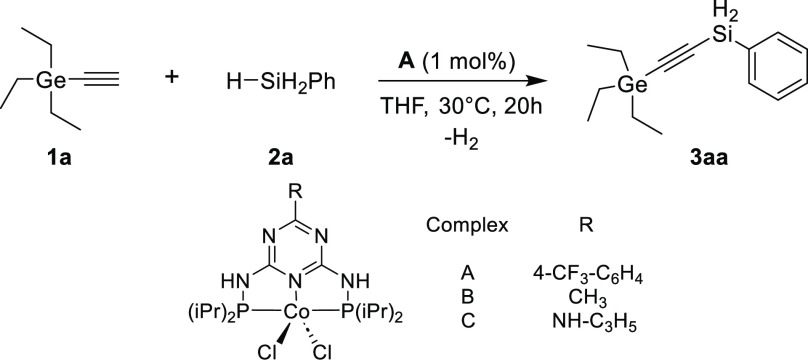
Optimization of Cobalt-Catalyzed Dehydrogenative Coupling with Primary Silanes^a^

entry	variation from standard conditions	conversion of **1a** [%]^b^	selectivity of **3aa** [%]
1	no change	99 (68)^c^	100
2	CoCl_2_ instead of **A**	0	0
3	without catalyst	0	0
4	**B** instead of **A**	96	60
5	**C** instead of **A**	97	33
6	in toluene	0	0
7	in toluene at 40 °C	0	0

a General reaction conditions: **1a** (1 equiv), **2a** (1.5 equiv), **A** (1 mol %), under an argon atmosphere, 30 °C.

b Conversion of **1a** determined by GC with *n*-dodecane as the internal standard.

c Isolated yield.

Initial success was obtained using phenylsilane as a silylating agent in the presence of 1 mol % complex **A** in THF at 30 °C. This combination of reagents afforded the desired product **3aa** with perfect selectivity in a 78% yield (Table 1, entry 1). The tests with other cobalt complexes (**B** and **C**) demonstrated that they were active but significantly less selective than **A** (Table 1, entries 4 and 5, respectively). A catalyst-free attempt was also performed and showed the crucial role of the cobalt precatalyst (Table 1, entry 3).

Cobalt chloride was also tested and found to be inactive in the studied process (Table 1, entry 2). The solvent screening at 30 and 40 °C showed that precatalyst **A** is inactive in toluene, whereas performing the reaction in other solvents led to lower efficiency (please see Table S1 in the Supporting Information, entry 6–13). With the optimized conditions in hand (Table 1, entry 1), we investigated the process of the dehydrogenative silylation of three examples of trisubstituted germylacetylenes (**1a**–**c**) with primary silanes such as phenylsilane (**2a**), hexylsilane (**2b**), *p*-tolylsilane (**2c**), octylsilane (**2d**), cyclohexylsilane (**2e**), and butylsilane (**2f**). The scope of the developed catalytic system proved to be broad (Scheme 2). Our attempts resulted in 16 products, which were obtained with good yields and high selectivity under mild conditions and at low catalyst concentrations. Nevertheless, to maintain the remarkable efficiency and high selectivity of the catalytic process, examples with bulky substituents required elevated temperatures.

**Scheme 2:**
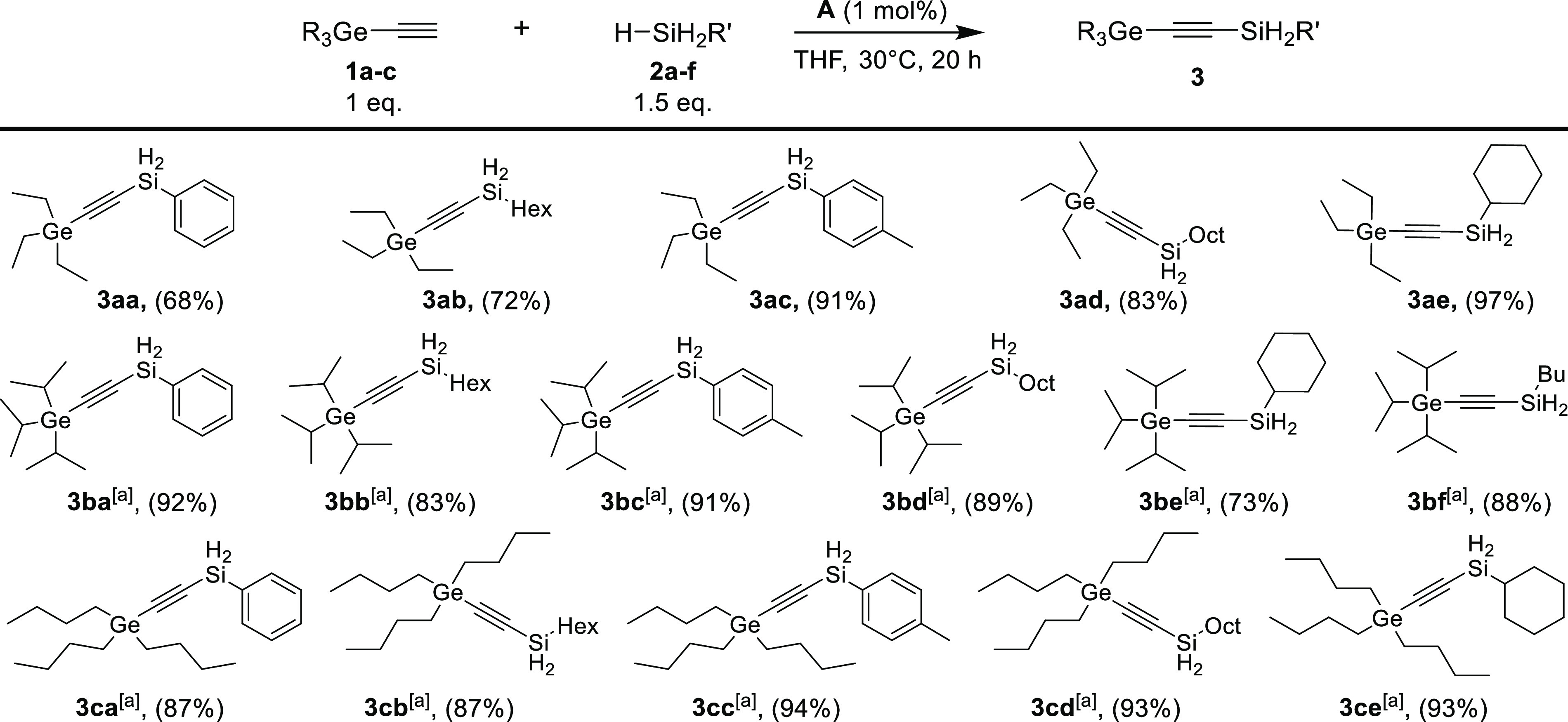
Scope of Dehydrogenative Coupling Products from Primary Silanes

Encouraged by the success of the reaction with primary silanes, we turned our attention toward secondary silanes, including methylphenylsilane (**2g**), diphenylsilane (**2h**) and diethylsilane (**2i**). However, previously established conditions did not bring any satisfactory results in the reaction of secondary silanes with triethylgermylacetylene (**1a**) or triisopropylgermylacetylene (**1b**) (Tables S2 and S3 in the Supporting Information). Increasing the concentration of the catalyst (Table S2 and S3, entry 4) also did not yield the desired product. Therefore, we changed our strategy and decided to investigate the influence of the addition of an activator, especially since it is well-known that metal borohydrides or metallic bases can serve as activators for cobalt complexes in organometallic synthesis.^10p,10t^ Preliminary experiments for the model reaction of triethylgermylacetylene or triisopropylgermylacetylene (**1b**) with diphenylsilane (**2h**) in the presence of **A** and different activators were conducted. The high conversion of substrates was only achieved in the presence of alkali metal alkoxides (Tables S2 and S3 in the Supporting Information), but surprisingly a mixture of hydrosilylation and dehydrogenative coupling products was observed.

However, further studies with a series of activators proved that it is possible to control the chemoselectivity of the reaction based on the substituent at the germanium atoms. First, we decided to optimize the reaction conditions with bulky triisopropylgermyloacetylene. Activator screening showed that carrying out the reaction in THF allows the reaction to proceed selectively toward the dehydrogenative coupling product (**4**) with only trace amounts of hydrosilylation product (**4′**). Ultimately, the best conditions were achieved using sodium *tert*-butoxide at 40 °C in THF, resulting in a nearly complete conversion of the substrate while maintaining very good chemoselectivity (Scheme 3). However, in the case of less sterically hindered triethylgermyloacetylene, the dominant hydrosilylation product was observed, which suggests the large influence of the steric effect on the catalytic cycle of the process (Scheme 4). Interestingly, in the case of primary silanes, the addition of an activator does not affect the selectivity of the process (please see Table S1 in the Supporting Information, entry 14). We have also demonstrated the utility of obtained unsaturated products with Si–H functionalities in the subsequent hydrosilylation process. A Karstedt-catalyzed Si–H addition to unsaturated systems enabled the synthesis of novel multifunctional compounds (Scheme 5).

**Scheme 3:**
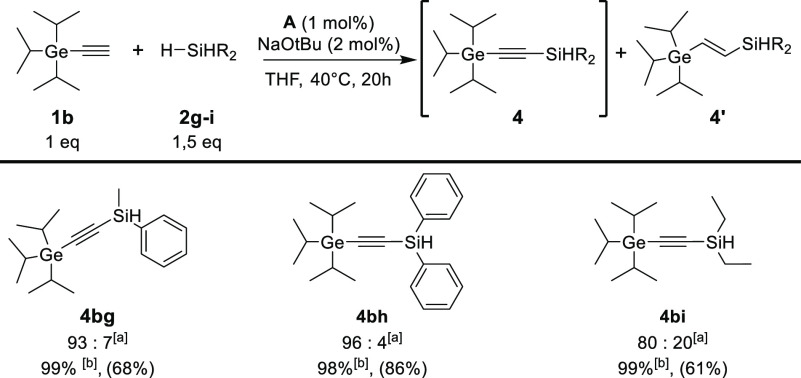
Scope of Dehydrogenative Coupling Products from Secondary Silanes

**Scheme 4:**
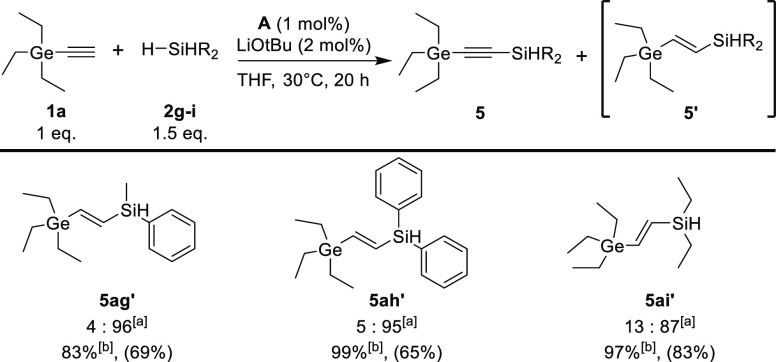
Scope of Hydrosilylation Reaction Products from Secondary Silanes

**Scheme 5:**
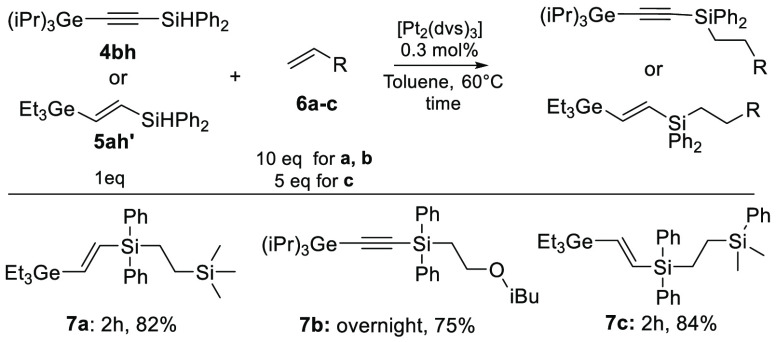
Pt-Catalyzed Hydrosilylation of Organogermanium Compounds

To gain some mechanistic insights into this 3d metal catalysis, we conducted preliminary experiments. Considering our previous investigations,^10r,10s^ again, we confirmed the formation of silyl Co–H species using ^1^H NMR analysis (please see the Supporting Information). In this regard, 10 equiv of **2a** was added to 1 equiv of precatalyst **A** in THF-*d*_8_, and the mixture was stirred at 40 °C for 24 h. The result indicates the generation of (PN^5^P)Co^III^H_2_(SiHPh_2_). Moreover, the Co^I^/Co^III^ pathway is in line with previous investigations.^10n−10s^ A plausible catalytic cycle based on previous literature and our experimental results is presented in Scheme 6.

**Scheme 6:**
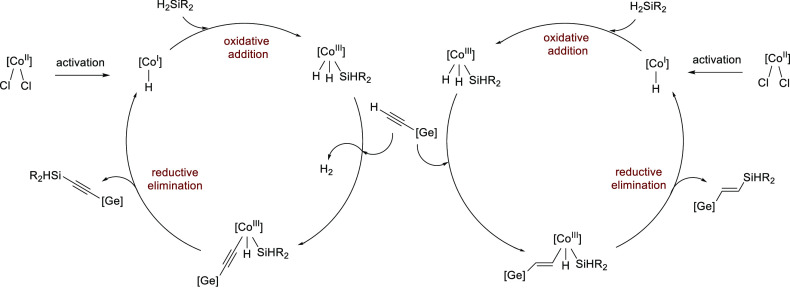
Proposed Catalytic Cycle

Secondary silanes can also activate our precatalyst. When in an NMR tube containing 1 equiv of precatalyst **A** and 10 equiv of H_2_SiPh_2_ in THF-*d*_8_ (stirred at 40 °C for 2 h), two [Co–H] species were registered (Supplement 2 in the Supporting Information). In an analogous reaction with the addition of an activator (LiOtBu), the active form of the catalyst is also formed and one [Co–H] species was observed (Supplement 3 in Supporting Information).

However, in the case of our precatalyst, such an activation mechanism for both the reactions with and without the addition of LiOtBu leads to the same active form of the complex, thus not explaining the differences in the activity that we observed. We suspect that, for our precatalysts, the addition of LiOtBu is probably responsible for the deprotonation of the NH groups of the complex (please compare the chemical shifts of the ^1^H NMR Co–H species; Supplements 2 and 3 in the Supporting Information) in a manner analogous to that proposed in the studies of the Kempe group, in which they postulate that the anionic nature of the catalyst affects its high activity.^12^ Therefore, we believe that the deprotonated form of the complex is fully responsible for the high catalytic activity and the steric effect determines whether the reaction proceeds by dehydrogenative coupling (in the case of **1b**) or hydrosilylation (in the case of **1a**).

In conclusion, we have established a new highly efficient method to obtain functional unsaturated organogermanium compounds in the presence of a cobalt catalyst. A series of silylgermyalcetylenes (19 compounds) and silylated vinylgermanes (3 compounds) have been obtained in high to excellent yields. The pincer-type cobalt complexes used in this work provide an attractive cost-efficient, mild, and highly chemoselective method for the synthesis of unsaturated germanes. It is worth noting that the reaction between secondary silanes and alkynylgermanes can proceed by dehydrogenative coupling or hydrosilylation depending on the substrates used. The described procedure offers a versatile method for obtaining a diverse range of compounds with potential industrial and synthetic applications.

## Data Availability

The data underlying this study are available in the published article and its Supporting Information

## Supporting Information Available

NMR spectra and characterization, general procedures, and mechanistic studies (PDF)
