# Supplementary material for: Access to Unsaturated Organogermanes via (De)Hydrosilylation Mediated by Cobalt Complexes
Source: Org Lett. 2023 Aug 30;25(35):6528–33. doi: 10.1021/acs.orglett.3c02326 (PMC10496132; doi:10.1021/acs.orglett.3c02326)
Supplement: Supplementary file 1 — ol3c02326_si_001.pdf [file ol3c02326_si_001.pdf]

Supporting Information

Access to Unsaturated Organogermanes via (De)hydrosilylation Mediated by Cobalt Complexes

Konstancja Broniarz[a], Grzegorz Hreczycho[a]

[a] M.Sc. K. Broniarz, Prof. Dr G. Hreczycho

Faculty of Chemistry, Adam Mickiewicz University, Uniwersytetu Poznanskiego St. 8, 61-614 Poznan, Poland.

Spis treści

GENERAL INFORMATION....4

OPTIMIZATION OF REACTION CONDITIONS....5

GENERAL SYNTHETIC PROCEDURES....8

CHARACTERISATION DATA FOR ALL PRODUCTS....11

Phenyl((triethylgermyl)ethynyl)silane (3aa)....11

Hexyl((triethylgermyl)ethynyl)silane (3ab)....11

p-Tolyl((triethylgermyl)ethynyl)silane (3ac)....12

Octyl((triethylgermyl)ethynyl)silane (3ad)....12

Cyclohexyl((triethylgermyl)ethynyl)silane (3ae)....13

Phenyl((triisopropylgermyl)ethynyl)silane (3ba)....13

Hexyl((triisopropylgermyl)ethynyl)silane (3bb)....14

p-Tolyl((triisopropylgermyl)ethynyl)silane (3bc)....14

Octyl((triisopropylgermyl)ethynyl)silane (3bd)....15

Cyclohexyl((triisopropylgermyl)ethynyl)silane (3be)....15

Butyl((triisopropylgermyl)ethynyl)silane (3bf)....16

Phenyl((tributylgermyl)ethynyl)silane (3ca)....16

Hexyl((tributylgermyl)ethynyl)silane (3cb)....17

p-Tolyl((tributylgermyl)ethynyl)silane (3cc)....17

Octyl((tributylgermyl)ethynyl)silane (3cd)....18

Cyclohexyl((tributylgermyl)ethynyl)silane (3ce)....18

Methyl(phenyl)((triisopropylgermyl)ethynyl)silane (4bg)....19

Diphenyl((triisopropylgermyl)ethynyl)silane (4bh)....19

Diethyl((triisopropylgermyl)ethynyl)silane (4bi)....20

Methyl(phenyl)(2-(triethylgermyl)vinyl)silane (5ag')....20

Diphenyl(2-(triethylgermyl)vinyl)silane (5ah')....21

Diethyl(2-(triethylgermyl)vinyl)silane (5ai')....21

(2-(diphenyl(2-(triethylgermyl)vinyl)silyl)ethyl)trimethylsilane (6a)....22

(2-isobutoxyethyl)diphenyl((triisopropylgermyl)ethynyl)silane (6b)....22

(2-(dimethyl(phenyl)silyl)ethyl)diphenyl(2-(triethylgermyl)vinyl)silane (6c)....23

SPECTRA FOR ALL PRODUCTS....24

Phenyl((triethylgermyl)ethynyl)silane (3aa)....24

Hexyl((triethylgermyl)ethynyl)silane (3ab)....26

p-Tolyl((triethylgermyl)ethynyl)silane(3ac)....28

Octyl((triethylgermyl)ethynyl)silane (3ad)....30

Cyclohexyl((triethylgermyl)ethynyl)silane (3ae)....32

Phenyl((triisopropylgermyl)ethynyl)silane (3ba)....34

Hexyl((triisopropylgermyl)ethynyl)silane (3bb) 36
p-Tolyl((triisopropylgermyl)ethynyl)silane (3bc) 38
Octyl((triisopropylgermyl)ethynyl)silane (3bd) 40
Cyclohexyl((triisopropylgermyl)ethynyl)silane (3be) 42
Butyl((triisopropylgermyl)ethynyl)silane (3bf) 44
Phenyl((tributylgermyl)ethynyl)silane (3ca) 46
Hexyl((tributylgermyl)ethynyl)silane (3cb) 48
p-Tolyl((tributylgermyl)ethynyl)silane (3cc) 50
Octyl((tributylgermyl)ethynyl)silane (3cd) 52
Cyclohexyl((tributylgermyl)ethynyl)silane (3ce) 54
Methyl(phenyl)((triisopropylgermyl)ethynyl)silane (4bg) 56
Diphenyl((triisopropylgermyl)ethynyl)silane (4bh) 58
Diethyl((triisopropylgermyl)ethynyl)silane (4bi) 60
Methyl(phenyl)(2-(triethylgermyl)vinyl)silane (5ag') 62
Diphenyl(2-(triethylgermyl)vinyl)silane (5ah') 64
Diethyl(2-(triethylgermyl)vinyl)silane (5ai') 66
(2-(diphenyl(2-(triethylgermyl)vinyl)silyl)ethyl)trimethylsilane (6a) 68
(2-isobutoxyethyl)diphenyl((triisopropylgermyl)ethynyl)silane (6b) 70
(2-(dimethyl(phenyl)silyl)ethyl)diphenyl(2-(triethylgermyl)vinyl)silane (6c) 72
MECHANISTIC STUDIES 74
Supplement 1 74
Supplement 2 75
Supplement 3 76

GENERAL INFORMATION

Air- and moisture-sensitive reactions were carried out under argon atmosphere using standard Schlenk techniques or a glove box. Solvents used for all experiments were purchased from Honeyweel or Sigma Aldrich (Merck), dried over calcium hydride (CaH_2) and purified by distillation. Tetrahydrofurane was additionally dried over sodium with a benzophenone system. All alkali metal compounds (lithium tert-butoxide, sodium tert-butoxide, potassium tert-butoxide) were purchased in the solid state from Sigma Aldrich (Merck) or StanLab. Ligands and Co-complexes were prepared in accordance with previously reported methods[1], using reagents purchased from Sigma Aldrich (Merck) or ABCR GmBH. Commercially available hydrosilanes (phenylsilane, n-hexylsilane, n-butylsilane, methylphenylsilane, diphenylsilane and diethylsilane) and olefins (vinyltrimethylsilane, dimethyphenylvinylsilane, isobutyl vinyl ether) were purchased from Sigma Aldrich (Merck), ABCR GmBH, Ambeed, Apollo Scientific or Acros Organics, dried over calcium hydride and purified by distillation. Other hydrosilanes (e.g. cyclohexylsilane, octylsilane) were synthesized from corresponding trichlorosilanes by well-known procedure utilizing $LiAlH_4$ as a reducing agent. Chloro- and bromogermanes were purchased from Gelest, ABCR GmBH and were used with ethynylmagnesium bromide solution in THF (Grignard reagent) for synthesis of alkynylgermanes (triethyl(ethynyl)germane, ethynyltriisopropylgermane, tributyl(ethynyl)germane) by well-known procedure. Column chromatography was performed using silica gel 60 A (230-400 mesh particle size) purchased from Sigma Aldrich (Merck)

Reaction progress (conversion of alkynylgermanes and hydrosilanes) was monitored by GC chromatography using Bruker Scion 460-GC and Agilent 5977B GC/MSD with Agilent 8860 GC System. The structures of products were determined by NMR spectroscopy and MS spectrometry. The ^{1}H NMR (400 or 600 MHz), ^{13}C NMR (101 or 151 MHz) and ^{29}Si NMR (79 or 119 MHz) spectra were recorded on Bruker Avance III HD NanoBay spectrometer, using chloroform-d1 ($CDCl_3$) or tetrahydrofuran-d_8 (THF-d_8) as solvent. Deuterated solvents were purchased from respectively Deutero GmbH (CDCl3 99.6 atom% D), Sigma Aldrich (Merck) (THF-d_8 99.5 atom% D) and used as received.

(1) Deibl, N.; Kempe, R. General and Mild Cobalt-Catalyzed C-Alkylation of Unactivated Amides and Esters with Alcohols. *J Am Chem Soc* **2016**, *138* (34), 10786–10789. DOI: 10.1021/jacs.6b06448.

OPTIMIZATION OF REACTION CONDITIONS

Table S1. Optimization of cobalt catalyzed dehydrogenative coupling with primary silanes.[a]

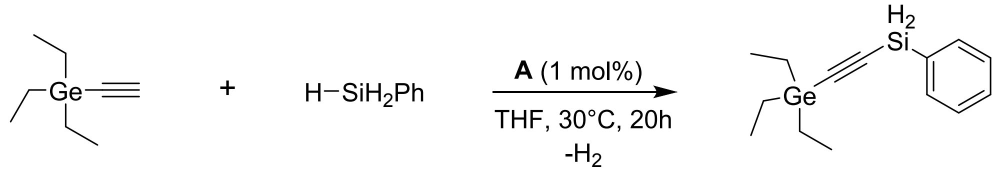

1a 2a 3aa

Examined cobalt complexes:

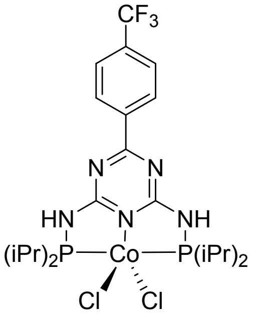

A

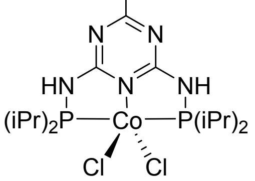

B

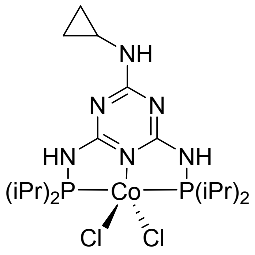

C

Entry	Variation from standard conditions	Conversion of 1a [%][b]	Selectivity of 3aa [%]
1	No change	97 (68)[c]	100
2	$CoCl_2$ instead of **A**	0	0
3	no catalyst	0	-
4	**B** instead of **A**	97	60
5	**C** instead of **A**	99	33
6	In toluene	0	0
7	In toluene at 40°C	0	0
8	In chlorobenzene	0	-
9	In chlorobenzene at 40°C	0	-
10	In diglyme	85	86
11	In diglyme at 40°C	89	95
12	Solvent-free	0	-
13	Solvent-free at 40°C	4	0
14	A in THF with NaO*t*Bu at 30°C	95	100

[a] General reaction conditions: **1a** (1 eq.), **2a** (1.5 eq.), **A** (1 mol%), under argon atmosphere, 30°C. [b] Conversion of 1a determined by GC with n-dodecane as the internal standard. [c] Isolated yield

Table S2. Optimization of cobalt catalyzed coupling of ethynyltriisopropylgermane with dihydrosilanes.

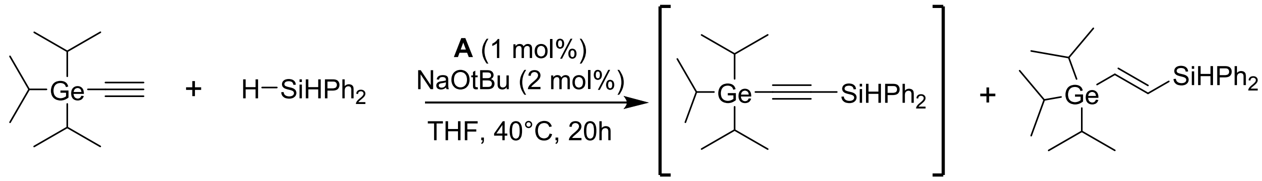

Entry	Variation from standard conditions	Conversion of 1b [%][b]	Selectivity [%] [4bh] : [4bh']][c]
1	No change	99	96 : 4
2	Without activator	0	-
3	Only NaO^{t}Bu	0	-
4	5 mol% of **A**, without activator	0	-
5	H_3SiPh instead of NaO^{t}Bu	0	-
6	$NaHBEt_3$ instead of NaO^{t}Bu	0	-
7	KO^{t}Bu instead of NaO^{t}Bu	99	87 : 13
8	LiO^{t}Bu instead of NaO^{t}Bu	37	77 : 23
9	2 mol% of **A**, 4 mol% of NaO^{t}Bu	67	89 : 11

[a] General reaction conditions: **1a** (1 eq.), **2h** (1.5 eq.), **A** (1 mol%), under argon atmosphere, 40°C. [b] Conversion of 1b determined by GC with n-dodecane as the internal standard. [c] Selectivity of dehydrogenative coupling and hydrosililation products determined by GC-MS.

Table S3. Optimization of cobalt catalyzed coupling of triethyl(ethynyl)germane with dihydrosilanes.

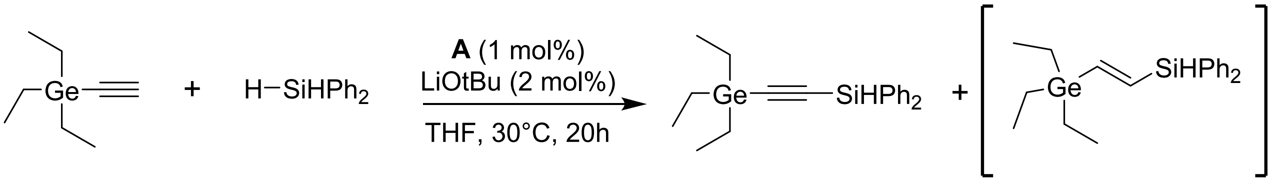

1a 2h 5ah 5ah'

Entry	Variation from standard conditions	Conversion of 1a [%][b]	Selectivity [%] [5aa] : [5aa'][c]
1	No change	90	2 : 98
2	Without activator	0	-
3	Only NaO*t*Bu	0	-
4	5 mol% of A, without activator	0	-
5	H_3SiPh instead of LiO*t*Bu	25	0:0
6	$NaHBEt_3$ instead of LiO*t*Bu	29	32 : 68
7	KO*t*Bu instead of LiO*t*Bu	55	45 : 55
8	NaO*t*Bu instead of LiO*t*Bu	17	42 : 58

[a] General reaction conditions: **1a** (1 eq.), **2h** (1.5 eq.), **A** (1 mol%), under argon atmosphere, 30°C. [b] Conversion of 1a determined by GC with n-dodecane as the internal standard. [c] Selectivity of dehydrogenative coupling and hydrosililation products determined by GC-MS.

GENERAL SYNTHETIC PROCEDURES

General procedure for cobalt catalyzed dehydrogenative coupling of alkynylgermanes with primary silanes.

<u>The synthesis of compounds **3aa**</u>

To a 12 mL vial equipped with magnetic stirring bar, 0.03M solution of pre-catalyst **A** (1 mol%, 0.01 mmol, 0.01 eq) in THF, phenylsilane (1.5 mmol, 162.3 mg, 1.5 eq), triethyl(ethynyl)germane (1 mmol, 184.8 mg, 1 eq) were added under inert gas atmosphere (glove box). Subsequently, reaction mixture was placed in a heating block and stirred at 30°C for 20 h. After this time, the catalyst was precipitated by addition of pentane or hexane (2 mL) and filtered from the mixture. In the next step, solvent and volatile residues were evaporated under a high vacuum giving desired product. The pure product was identified by ^{1}H, ^{13}C, ^{29}Si spectroscopies and MS spectrometry

<u>The synthesis of compounds **3ab, 3ac, 3ad, 3ae**</u>

To a 12 mL vial equipped with magnetic stirring bar, 0.03M solution of pre-catalyst **A** (1 mol%, 0.54 µmol, 0.01 eq) in THF, primary silane (0.08mmol, 1.5 eq of hexylsilane or 0.08 mmol, 1.5 eq of p-tolylsilane or 0.08 mmol, 1.5 eq of cyclohexylsilane or 0.08 mmol, 1.5 eq of octylsilane), triethyl(ethynyl)germane (0.053 mmol, 1 eq) were added under inert gas atmosphere (glove box). Subsequently, reaction mixture was placed in a heating block and stirred at 30°C for 20 h. After this time, the catalyst was precipitated by addition of pentane or hexane (1 mL) and filtered from the mixture. In the next step, solvent and volatile residues were evaporated under a high vacuum giving desired product. The pure products were identified by ^{1}H, ^{13}C, ^{29}Si spectroscopies and MS spectrometry.

<u>The synthesis of compounds **3ba, 3bb, 3bc, 3bd, 3be, 3bf, 3ca, 3cb, 3cc, 3cd, 3ce**</u>

To a 12 mL vial equipped with magnetic stirring bar, 0.03M solution of pre-catalyst A (1 mol%, 0.47 µmol, 0.01 eq) in THF , primary silane (0.07 mmol, 1.5 eq of phenylsilane or 0.07 mmol, 1.5 eq of hexylsilane or 0.07 mmol, 1.5 eq of p-tolylsilane or 0.07 mmol, 1.5 eq of cyclohexylsilane or 0.07 mmol, 1.5 eq of octylsilane or 0.07 mmol, 1.5 eq of n-butylsilane), ethynyltriisopropylgermane (0.047 mmol, 1 eq) or tributyl(ethynyl)-germane (0.047 mmol, 1 eq) were added under inert gas atmosphere (glove box). Subsequently, reaction mixture was placed in a heating block and stirred at 40°C for 20 h. After this time, the catalyst was precipitated by addition of pentane or hexane (1 mL) and filtered from the mixture. In the next step, solvent and volatile residues were evaporated under a high vacuum giving desired product. The pure products were identified by ^{1}H, ^{13}C, ^{29}Si spectroscopies and MS spectrometry.

General procedure for cobalt catalyzed dehydrogenative coupling of alkynylgermanes with secondary silanes.

The synthesis of compounds **4bg, 4bh, 4bi**

To a 12 mL vial equipped with magnetic stirring bar, 0.06M solution of pre-catalyst A (1 mol%, 0.47 µmol, 0.01 eq or 1.5 mol%, 0.7 µmol, 0.015 eq for diethylsilane) in THF, 0.06M solution of sodium tert-butoxide (2 mol%, 0.94 µmol, 0.02 eq or 3 mol%, 1.41 µmol, 0.03 eq for diethylsilane) in THF, secondary silane (0.07 mmol, 1.5 eq of diphenylsilane or 0.07mmol, 1.5 eq of methylphenylsilane or 0.07 mmol, 1.5 eq of diethylsilane) ethynyltriisopropylgermane (0.047 mmol, 1 eq) were added under inert gas atmosphere (glove box). Subsequently, reaction mixture was placed in a heating block and stirred at 40°C for 20 h. After this time, the catalyst was precipitated by addition of pentane or hexane (1 mL) and filtered from the mixture. In the next step, solvent and volatile residues were evaporated under a high vacuum. The product was purified by silica gel column chromatography (eluent used: hexane). Lastly, solvent was evaporated under high vacuum giving desired products. The pure products were identified by ^{1}H, ^{13}C, ^{29}Si spectroscopies and MS spectrometry.

General procedure for cobalt catalyzed hydrosilylation of alkynylgermanes with secondary silanes.

The synthesis of compounds **5ag', 5ah', 5hi**

To a 12 mL vial equipped with magnetic stirring bar, 0.06M solution of pre-catalyst A (1 mol%, 0.54 µmol, 0.01 eq or 1.5 mol%, 0.81 µmol, 0.015 eq for diethylsilane) in THF, 0.06M solution of lithium tert-butoxide (2 mol%, 1.08 µmol, 0.02 eq or 3 mol%, 1.62 µmol, 0.03 eq for diethylsilane) in THF, secondary silane (0.08 mmol, 1.5 eq of diphenylsilane or 0.08 mmol, 1.5 eq of methylphenylsilane or 0.08 mmol, 1.5 eq of diethylsilane), triethyl(ethynyl)germane (0.053 mmol, 1 eq) were added under inert gas atmosphere (glove box). Subsequently, reaction mixture was placed in a heating block and stirred at 30°C for 20 h. After this time, the catalyst was precipitated by addition of pentane or hexane (1 mL) and filtered from the mixture. In the next step, solvents and volatile residues were evaporated under a high vacuum. The residue mixture was purified by silica gel column chromatography (eluent used: hexane). Lastly, solvent was evaporated under high vacuum giving desired products. The pure products were identified by ^{1}H, ^{13}C, ^{29}Si spectroscopies and MS spectrometry.

General procedure for hydrosilylation catalyzed by Karstedt catalyst:

The synthesis of **6a**

To a 25 mL Schlenk tube equipped with a magnetic stirring bar, diphenyl(2-(triethylgermyl)vinyl)silane (0.17 mmol, 63 mg, 1 eq), toluene (100 µL), vinyltrimethylsilane (1.73 mmol, 253.2 µL, 10 eq) and Karstedt catalyst (0.3 mol%) were added under argon atmosphere. Reaction mixture was stirred at 60°C in an oil bath for 2 hours. After that, solvent and volatile residues were evaporated under high vacuum. Catalyst was precipitated by the addition of hexane (1 mL), filtered from the mixture. Solvent was evaporated under reduced pressure giving desired product **6a**. The obtained product was identified ^{1}H, ^{13}C, ^{29}Si spectroscopies and MS spectrometry.

The synthesis of **6b**

To a 25 mL Schlenk tube equipped with a magnetic stirring bar diphenyl((triisopropylgermyl)ethynyl)silane (0.085 mmol, 35 mg, 1 eq), toluene (100 µL), isobutyl vinyl ether (0.85 mmol, 85,6 mg, 10 eq) and Karstedt catalyst (0.3 mol%) were added under argon atmosphere. Reaction mixture was stirred at 60°C in an oil bath overnight. After that, solvent and volatile residues were evaporated under high vacuum. Catalyst was precipitated by the addition of hexane (1 mL), filtered from the mixture. Solvent was evaporated under reduced pressure giving desired product **6b**. The obtained product was identified ^{1}H, ^{13}C, ^{29}Si spectroscopies and MS spectrometry.

The synthesis of **6c**

To a 25 mL Schlenk tube equipped with a magnetic stirring bar, diphenyl(2-(triethylgermyl)vinyl)silane (0.17 mmol, 65 mg, 1 eq), toluene (100 µL), dimethylphenylvinylsilane (0.88 mmol, 143 mg, 5 eq) and Karstedt catalyst (0.3 mol%) were added under argon atmosphere. Reaction mixture was stirred at 60°C in an oil bath for 2 hours. After that, solvent and volatile residues were evaporated under high vacuum. Catalyst was precipitated by the addition of hexane (1 mL), filtered from the mixture. Solvent was evaporated under reduced pressure giving desired product **6c**. The obtained product was identified ^{1}H, ^{13}C, ^{29}Si spectroscopies and MS spectrometry.

CHARACTERISATION DATA FOR ALL PRODUCTS

Phenyl((triethylgermyl)ethynyl)silane (3aa)

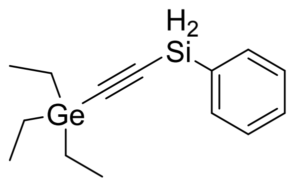

Phenyl((triethylgermyl)ethynyl)silane was obtained as a liquid in 68% yield (201.9 mg). The title compound was previously unknown.

^{1}H NMR: (600 MHz, Chloroform-d) δ 7.80 – 7.59 (m, 2H), 7.48 – 7.40 (m, 3H), 4.63 (s, 2H), 1.15 (t, *J* = 7.9 Hz, 9H), 0.95 (q, 6H).

^{13}C NMR: (151 MHz, Chloroform-d) δ 135.2, 130.0, 129.7, 128.1, 118.5, 103.5, 9.0, 5.7.

^{29}Si NMR: (79 MHz, Chloroform-d) δ -62.55.

EI-MS m/z (rel. int.): 263 (100%, $[M-Et]^{+}$), 235 (45), 204 (22), 150 (20), 105 (23)

EA: $C_{14}H_{22}GeSi$ (292.070): calcd. 57.78; H, 7.62; found C 57.67, H 7.57.

Hexyl((triethylgermyl)ethynyl)silane (3ab)

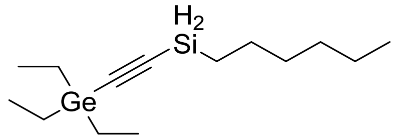

Hexyl((triethylgermyl)ethynyl)silane was obtained as a liquid in 72% yield (11.6 mg). The title compound was previously unknown.

^{1}H NMR: (400 MHz, Chloroform-d) δ 3.94 (t, *J* = 3.6 Hz, 2H), 1.51 – 1.43 (m, 2H), 1.40 – 1.34 (m, 2H), 1.31 – 1.26 (m, 4H), 1.09 (t, 9H), 0.90 – 0.86 (m, 8H), 0.84 – 0.75 (m, 3H).

^{13}C NMR: (101 MHz, Chloroform-d) δ 116.6, 105.4, 32.3, 31.6, 24.9, 22.7, 14.2, 10.1, 9.1, 5.8.

^{29}Si NMR: (79 MHz, Chloroform-d) δ -59.14.

EI-MS m/z (rel. int.): 271 (100%, $[M-Et]^{+}$), 156,9 (21), 128,9 (43), 100,9 (18)

EA: $C_{14}H_{30}GeSi$ (300.133): calcd. 56.22; H, 10.11; found C 56.42, H 10.01.

p-Tolyl((triethylgermyl)ethynyl)silane (3ac)

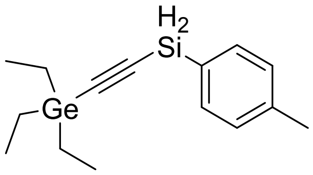

p-Tolyl((triethylgermyl)ethynyl)silane was obtained as a liquid in 91% yield (15 mg). The title compound was previously unknown.

^{1}H NMR: (400 MHz, Chloroform-d) δ 7.62 – 7.58 (m, 2H), 7.25 – 7.22 (m, 2H), 4.61 (s, 2H), 2.39 (s, 3H), 1.14 (t, 9H), 0.93 (q, 6H).

^{13}C NMR: (101 MHz, Chloroform-d) δ 140.0, 135.2, 128.9, 125.8, 118.1, 103.8, 21.5, 8.9, 5.6.

^{29}Si NMR: (79 MHz, Chloroform-d) δ -62.73.

EI-MS m/z (rel. int.): 277 (100%, $[M\text{-}Et]^+$), 249 (29), 219 (14), 164,9 (13), 6,9 (12)

EA: $C_{15}H_{24}GeSi$ (306.086): calcd. 59.06; H, 7.93; found C 59.14, H 7.99.

Octyl((triethylgermyl)ethynyl)silane (3ad)

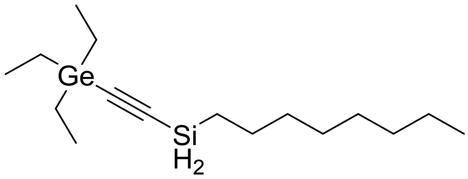

Octyl((triethylgermyl)ethynyl)silane was obtained as a liquid in 83% yield (14.7 mg). The title compound was previously unknown.

^{1}H NMR: (600 MHz, Chloroform-d) δ 3.97 (t, *J* = 3.5 Hz, 2H), 1.53 – 1.47 (m, 2H), 1.41 – 1.35 (m, 2H), 1.34 – 1.27 (m, 9H), 1.12 (t, *J* = 7.9 Hz, 9H), 0.92 – 0.88 (m, 8H), 0.84 – 0.80 (m, 2H).

^{13}C NMR: (151 MHz, Chloroform-d) δ 116.5, 105.3, 32.5, 31.9, 29.3, 29.2, 24.8, 22.7, 14.1, 10.0, 8.9, 5.6.

^{29}Si NMR: (119 MHz, Chloroform-d) δ -59.14.

EI-MS m/z (rel. int.): 299 (100%, $[M\text{-}Et]^+$), 195 (12), 156,9 (28), 128,9 (37), 100,9 (18)

EA: $C_{16}H_{34}GeSi$ (328.164): calcd. 58.74; H, 10.48; found C 58.65, H 10.49.

Cyclohexyl((triethylgermyl)ethynyl)silane (3ae)

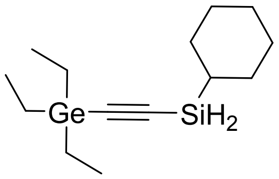

Cyclohexyl((triethylgermyl)ethynyl)silane was obtained as a liquid in 97% yield (15.5 mg). The title compound was previously unknown.

^{1}H NMR: (400 MHz, Chloroform-d) δ 3.85 (d, *J* = 2.8 Hz, 2H), 1.85 – 1.68 (m, 5H), 1.36 – 1.24 (m, 6H), 1.12 (t, *J* = 7.9 Hz, 9H), 0.89 (q, *J* = 7.7, 1.1 Hz, 6H).

^{13}C NMR: (101 MHz, Chloroform-d) δ 116.8, 104.4, 28.4, 27.5, 26.6, 21.6, 8.9, 5.7.

^{29}Si NMR: (79 MHz, Chloroform-d) δ -53.75.

EI-MS m/z (rel. int.): 269 (100%, [m-Et$^+$), 211 (11), 165 (40), 128,9 (40), 104,9 (18), 81 (25), 55 (22)

EA: $C_{14}H_{28}GeSi$ (298.177): calcd. 56.60; H, 9.50; found C 56.67, H 9.44.

Phenyl((triisopropylgermyl)ethynyl)silane (3ba)

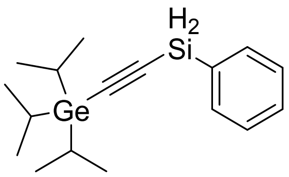

Phenyl((triisopropylgermyl)ethynyl)silane was obtained as a liquid in 92% yield (14.3 mg). The title compound was previously unknown.

^{1}H NMR: (600 MHz, Chloroform-d) δ 7.79 – 7.58 (m, 2H), 7.47 – 7.36 (m, 3H), 4.61 (s, 2H), 1.50 – 1.37 (m, 3H), 1.20 (d, *J* = 7.4 Hz, 18H).

^{13}C NMR: (151 MHz, Chloroform-d) δ 135.1, 130.0, 129.9, 128.1, 117.8, 104.2, 19.9, 14.9.

^{29}Si NMR: (119 MHz, Chloroform-d) δ -62.83.

EI-MS m/z (rel. int.): 291 (100%, [M-iPr]$^+$), 249 (37), 204,9 (19), 151 (28), 105 (20)

EA: $C_{17}H_{28}GeSi$ (334.177): calcd. 61.29; H, 8.47; found C 61.27, H 8.40.

Hexyl((triisopropylgermyl)ethynyl)silane (3bb)

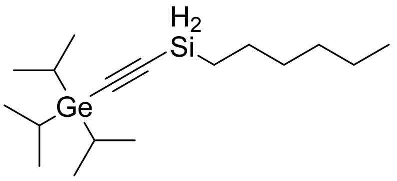

Hexyl((triisopropylgermyl)ethynyl)silane was obtained as a liquid in 83% yield (13.2 mg). The title compound was previously unknown.

^{1}H NMR: (400 MHz, Chloroform-d) δ 3.95 (t, *J* = 3.4 Hz, 2H), 1.53 – 1.44 (m, 2H), 1.43 – 1.33 (m, 5H), 1.31 – 1.24 (m, 4H), 1.17 (d, *J* = 7.5 Hz, 18H), 0.88 (t, *J* = 6.7 Hz, 3H), 0.83 – 0.75 (m, 2H).

^{13}C NMR: (101 MHz, Chloroform-d) δ 115.6, 106.0, 32.1, 31.5, 24.8, 22.5, 19.8, 14.8, 14.1, 10.1.

^{29}Si NMR: (79 MHz, Chloroform-d) δ -59.36.

EI-MS m/z (rel. int.): 299 (100%, [M-iPr]$^{+}$), 185 (20), 142,9 (24), 126,9 (39), 97 (18)

EA: $C_{17}H_{36}GeSi$ (342.180): calcd. 59.85; H, 10.64; found C 59.73, H 10.58.

p-Tolyl((triisopropylgermyl)ethynyl)silane (3bc)

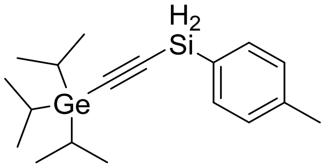

p-Tolyl((triisopropylgermyl)ethynyl)silane was obtained as a liquid in 91% yield (14.7 mg). The title compound was previously unknown.

^{1}H NMR: (400 MHz, Chloroform-d) δ 7.63 – 7.58 (m, 2H), 7.25 – 7.21 (m, 2H), 4.61 (s, 2H), 2.39 (s, 3H), 1.52 – 1.40 (m, 3H), 1.21 (d, *J* = 7.3 Hz, 18H).

^{13}C NMR: (101 MHz, Chloroform-d) δ 140.0, 135.1, 128.9, 126.0, 117.4, 104.5, 21.5, 19.8, 14.9.

^{29}Si NMR: (79 MHz, Chloroform-d) δ -63.02.

EI-MS m/z (rel. int.): 305 (100%, [M-iPr]$^{+}$), 263 (42), 219 (20), 164,9 (36), 119 (26), 91 (24)

EA: $C_{18}H_{30}GeSi$ (348.133): calcd. 62.28; H, 8.71; found C 62.32, H 8.69.

Octyl((triisopropylgermyl)ethynyl)silane (3bd)

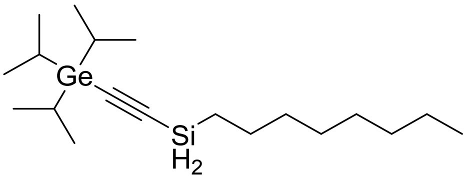

Octyl((triisopropylgermyl)ethynyl)silane was obtained as a liquid in 86% yield (15.4 mg). The title compound was previously unknown.

^{1}H NMR: (400 MHz, Chloroform-d) δ 3.95 (t, *J* = 3.5 Hz, 2H), 1.53 – 1.45 (m, 2H), 1.44 – 1.36 (m, 4H), 1.33 – 1.24 (m, 9H), 1.17 (d, *J* = 7.3 Hz, 18H), 0.91 – 0.86 (m, 3H), 0.84 – 0.76 (m, 2H).

^{13}C NMR: (101 MHz, Chloroform-d) δ 115.6, 106.0, 32.5, 31.9, 29.3, 29.2, 24.8, 22.7, 19.8, 14.8, 14.1, 10.1.

^{29}Si NMR: (79 MHz, Chloroform-d) δ -59.33.

EI-MS m/z (rel. int.): 327 (100%, [M-iPr]$^{+}$), 185 (13), 156,9 (20), 128,8 (28), 96,9 (20)

EA: $C_{19}H_{40}GeSi$ (370.211): calcd. 61.80; H, 10.92; found C 61.85, H 10.85.

Cyclohexyl((triisopropylgermyl)ethynyl)silane (3be)

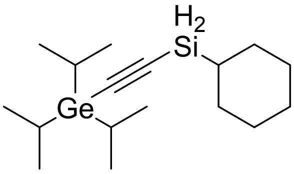

Cyclohexyl((triisopropylgermyl)ethynyl)silane was obtained as a liquid in 73% yield (11.6 mg). The title compound was previously unknown.

^{1}H NMR: (400 MHz, Chloroform-d) δ 3.86 (d, *J* = 2.7 Hz, 2H), 1.86 – 1.68 (m, 5H), 1.42 (h, *J* = 7.4 Hz, 3H), 1.35 – 1.25 (m, 6H), 1.19 (d, *J* = 7.4 Hz, 18H).

^{13}C NMR: (101 MHz, Chloroform-d) δ 116.0, 105.1, 28.4, 27.5, 26.6, 21.7, 19.8, 14.8.

^{29}Si NMR: (79 MHz, Chloroform-d) δ -53.87.

EI-MS m/z (rel. int.): 297 (100%, [M-iPr]$^{+}$), 211 (16), 170,9 (11), 126,9 (33), 81 (16), 55 (19)

EA: $C_{17}H_{34}GeSi$ (340.164): calcd. 60.20; H, 10.10; found C 60.18, H 10.07.

Butyl((triisopropylgermyl)ethynyl)silane (3bf)

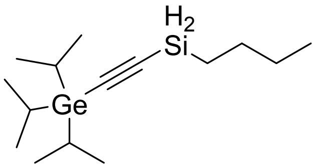

Butyl((triisopropylgermyl)ethynyl)silane was obtained as a liquid in 88% yield (12.9 mg). The title compound was previously unknown.

^{1}H NMR: (400 MHz, Chloroform-d) δ 3.88 (t, *J* = 3.5 Hz, 2H), 1.45 – 1.26 (m, 7H), 1.10 (d, *J* = 7.5 Hz, 18H), 0.83 (t, *J* = 7.2 Hz, 4H), 0.78 – 0.69 (m, 2H).

^{13}C NMR: (101 MHz, Chloroform-d) δ 115.7, 106.0, 27.0, 25.5, 19.8, 14.8, 13.8, 9.8.

^{29}Si NMR: (80 MHz, Chloroform-d) δ -59.30.

EI-MS m/z (rel. int.): 271 (100%, $[M\text{-}iPr]^{+}$), 229 (15), 184,9 (19), 159 (15), 126,9 (31), 111 (16)

EA: $C_{15}H_{32}GeSi$ (314.149): calcd. 57.54; H, 10.30; found C 57.61, H 10.33.

Phenyl((tributylgermyl)ethynyl)silane (3ca)

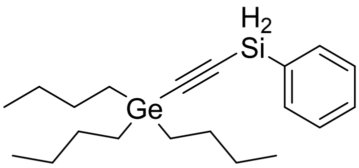

Phenyl((tributylgermyl)ethynyl)silane was obtained as a liquid in 87% yield (15.3 mg). The title compound was previously unknown.

^{1}H NMR: (600 MHz, Chloroform-d) δ 7.75 – 7.68 (m, 2H), 7.48 – 7.39 (m, 3H), 4.62 (s, 2H), 1.52 – 1.44 (m, 6H), 1.43 – 1.36 (m, 6H), 0.97 – 0.90 (m, 15H).

^{13}C NMR: (151 MHz, Chloroform-d) δ 135.2, 130.0, 129.7, 128.1, 119.5, 103.4, 27.4, 26.1, 14.0, 13.8.

^{29}Si NMR: (119 MHz, Chloroform-d) δ -62.58.

EI-MS m/z (rel. int.): 319 (100%, $[M\text{- }nBu]^{+}$), 263 (56), 206,8 (45), 187 (70), 130,9 (41)

EA: $C_{20}H_{34}GeSi$ (314.149): calcd. 64.02; H, 9.13; found C 64.11, H 9.09.

Hexyl((tributylgermyl)ethynyl)silane (3cb)

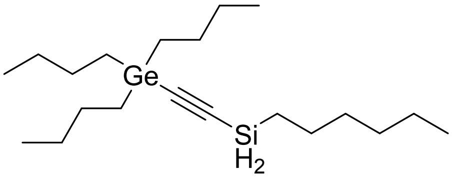

Hexyl((tributylgermyl)ethynyl)silane was obtained as a liquid in 87% yield (15.6 mg). The title compound was previously unknown.

^{1}H NMR: (600 MHz, Chloroform-d) δ 3.97 (t, *J* = 3.5 Hz, 2H), 1.55 – 1.23 (m, 21H), 1.00 – 0.77 (m, 19H).

^{13}C NMR: (151 MHz, Chloroform-d) δ 117.5, 105.1, 32.2, 31.5, 27.3, 26.1, 24.8, 22.5, 14.1, 14.0, 13.7, 10.0.

^{29}Si NMR: (119 MHz, Chloroform-d) δ -59.14.

EI-MS m/z (rel. int.): 327 (100%, $[M-Bu]^{+}$), 270,9 (29), 186,9 (37), 158,8 (22), 128,8 (54), 110,9 (32)

EA: $C_{20}H_{42}GeSi$ (384.227): calcd. 62.68; H, 11.05; found C 62.72, H 11.07.

p-Tolyl((tributylgermyl)ethynyl)silane (3cc)

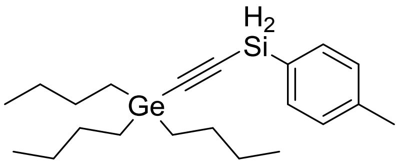

p-Tolyl((tributylgermyl)ethynyl)silane was obtained as a liquid in 94% yield (17.1 mg). The title compound was previously unknown.

^{1}H NMR: (600 MHz, Chloroform-d) δ 7.60 (d, *J* = 7.6 Hz, 2H), 7.24 (d, *J* = 7.4 Hz, 2H), 4.60 (s, 2H), 2.40 (s, 3H), 1.51 – 1.45 (m, 6H), 1.43 – 1.36 (m, 6H), 0.96 – 0.91 (m, 15H).

^{13}C NMR: (151 MHz, Chloroform-d) δ 140.1, 135.2, 128.9, 126.0, 119.2, 103.7, 27.4, 26.1, 21.6, 14.0, 13.6.

^{29}Si NMR: (119 MHz, Chloroform-d) δ -62.79.

EI-MS m/z (rel. int.): 333 (100%, $[M-Bu]^{+}$), 277 (73), 218,9 (39), 201 (53), 164,9 (39), 119 (28), 91 (19)

EA: $C_{21}H_{36}GeSi$ (390,180): calcd. 64.80; H, 9.32; found C 64.84, H 9.28.

Octyl((tributylgermyl)ethynyl)silane (3cd)

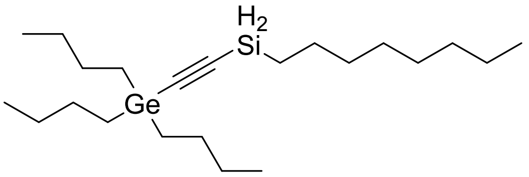

Octyl((tributylgermyl)ethynyl)silane was obtained as a liquid in 93% yield (17.9 mg). The title compound was previously unknown.

^{1}H NMR: (400 MHz, Chloroform-d) δ 3.94 (t, *J* = 3.5 Hz, 2H), 1.52 – 1.24 (m, 25H), 0.98 – 0.84 (m, 17H), 0.82 – 0.75 (m, 2H).

^{13}C NMR: (101 MHz, Chloroform-d) δ 117.4, 105.1, 32.5, 31.9, 29.3, 29.2, 27.3, 26.1, 24.8, 22.7, 14.1, 14.0, 13.7, 10.0.

^{29}Si NMR: (79 MHz, Chloroform-d) δ -59.10.

EI-MS m/z (rel. int.): 355 (100%, [M-nBu]$^{+}$), 299 (22), 187 (41), 154,8 (22), 128,9 (57), 111 (33), 98,9 (14), 54,9 (18)

EA: $C_{22}H_{46}GeSi$ (412,258): calcd. 64.24; H, 11.27; found C 64.21, H 11.25.

Cyclohexyl((tributylgermyl)ethynyl)silane (3ce)

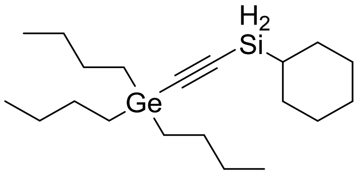

Cyclohexyl((tributylgermyl)ethynyl)silane was obtained as a liquid in 93% yield (16.6 mg). The title compound was previously unknown.

^{1}H NMR: (400 MHz, Chloroform-d) δ 3.85 (d, *J* = 2.8 Hz, 2H), 1.86 – 1.68 (m, 6H), 1.50 – 1.27 (m, 18H), 0.95 – 0.86 (m, 15H).

^{13}C NMR: (101 MHz, Chloroform-d) δ 117.9, 104.6, 28.4, 27.6, 27.4, 26.6, 26.1, 21.6, 14.0, 13.7.

^{29}Si NMR: (79 MHz, Chloroform-d) δ -53.73.

EI-MS m/z (rel. int.): 325 (100%, [M-nBu]$^{+}$), 269 (32), 184,9 (35), 156,9 (16), 128,9 (44), 111 (22), 81 (23), 55 (31)

EA: $C_{20}H_{40}GeSi$ (382.211): calcd. 63.01; H, 10.58; found C 63.11, H 10.49.

Methyl(phenyl)((triisopropylgermyl)ethynyl)silane (4bg)

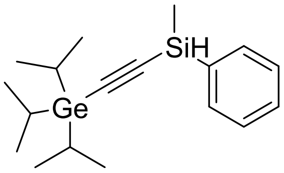

Methyl(phenyl)((triisopropylgermyl)ethynyl)silane was obtained as a liquid in 68% yield (11 mg). The title compound was previously unknown.

^{1}H NMR: (600 MHz, Chloroform-d) δ 7.73 – 7.68 (m, 2H), 7.45 – 7.38 (m, 3H), 4.67 (q, *J* = 3.8 Hz, 1H), 1.48 – 1.42 (m, 3H), 1.21 (d, *J* = 7.4 Hz, 18H), 0.50 (d, *J* = 3.8 Hz, 3H).

^{13}C NMR: (151 MHz, Chloroform-d) δ 134.4, 134.3, 129.6, 127.9, 115.4, 109.0, 19.9, 14.9, -3.1.

^{29}Si NMR: (119 MHz, Chloroform-d) δ -41.37.

EI-MS m/z (rel. int.): 305 (100%, [M-iPr]$^+$), 263 (41), 218,9 (19), 145 (29), 121 (17), 105 (13)

EA: $C_{18}H_{30}GeSi$ (348.133): calcd. 62.28; H, 8.71; found C 62.20, H 8.75.

Diphenyl((triisopropylgermyl)ethynyl)silane (4bh)

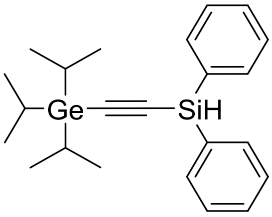

Diphenyl((triisopropylgermyl)ethynyl)silane was obtained as a liquid in 86% yield (16.4 mg). The title compound was previously unknown.

^{1}H NMR: (400 MHz, Chloroform-d) δ 7.64 – 7.59 (m, 4H), 7.34 – 7.25 (m, 6H), 5.08 (s, 1H), 1.44 – 1.32 (m, 3H), 1.13 (d, 18H).

^{13}C NMR: (101 MHz, Chloroform-d) δ 135.1, 132.9, 129.9, 128.0, 117.6, 106.9, 19.9, 15.0.

^{29}Si NMR: (80 MHz, Chloroform-d) δ -42.50.

EI-MS m/z (rel. int.): 367 (100%,[M-iPr]$^+$), 324,9 (25), 207 (24), 183 (16), 150 (20), 78 (13)

EA: $C_{23}H_{32}GeSi$ (410.149): calcd. 67.51; H, 7.88; found C 67.44, H 7.81.

Diethyl((triisopropylgermyl)ethynyl)silane (4bi)

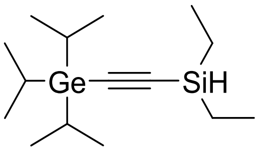

Diethyl((triisopropylgermyl)ethynyl)silane was obtained as a liquid in 61% yield (8.9 mg). The title compound was previously unknown.

^{1}H NMR: (400 MHz, Chloroform-d) δ 3.87 (p, *J* = 3.2 Hz, 1H), 1.38 – 1.26 (m, 3H), 1.09 (d, *J* = 7.3 Hz, 18H), 0.98 (t, 6H), 0.66 – 0.56 (m, 4H).

^{13}C NMR: (101 MHz, Chloroform-d) δ 113.6, 109.4, 19.9, 14.8, 7.9, 4.1.

^{29}Si NMR: (79 MHz, Chloroform-d) δ -28.20.

EI-MS m/z (rel. int.): 271 (100%, [M-iPr]$^{+}$), 229 (27), 201 (22), 156,9 (15), 126,9 (23)

EA: $C_{15}H_{32}GeSi$ (314.149): calcd. 57.54; H, 10.30; found C 57.50, H 10.21.

Methyl(phenyl)(2-(triethylgermyl)vinyl)silane (5ag')

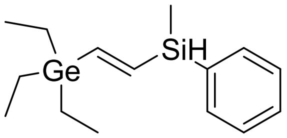

Methyl(phenyl)(2-(triethylgermyl)vinyl)silane was obtained as a liquid in 69%% yield (11.5 mg). The title compound was previously unknown.

^{1}H NMR: (400 MHz, Chloroform-d) δ 7.63 – 7.54 (m, 2H), 7.45 – 7.37 (m, 3H), 6.99 (d, *J* = 22.1 Hz, 1H), 6.66 (dd, *J* = 22.1, 2.6 Hz, 1H), 4.64 – 4.59 (m, 1H), 1.08 (t, *J* = 7.9 Hz, 9H), 0.87 (q, 6H), 0.47 (d, *J* = 3.8 Hz, 3H).

^{13}C NMR: (101 MHz, Chloroform-d) δ 153.4, 144.4, 136.0, 134.6, 129.3, 127.9, 8.9, 4.1, -5.4.

^{29}Si NMR: (79 MHz, Chloroform-d) δ -22.64.

EI-MS m/z (rel. int.): 279 (100%, [M-Et]$^{+}$), 222,9 (13), 145 (54), 121 (41), 104,9 (36)

EA: $C_{15}H_{26}GeSi$ (308.102): calcd. 58.67; H, 8.53; found C 58.69, H 8.47.

Diphenyl(2-(triethylgermyl)vinyl)silane (5ah’)

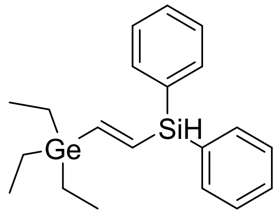

Diphenyl(2-(triethylgermyl)vinyl)silane was obtained as a liquid in 65% yield (12.9 mg). The title compound was previously unknown.

^{1}H NMR: (400 MHz, Chloroform-d) δ 7.52 – 7.44 (m, 4H), 7.35 – 7.24 (m, 6H), 6.94 (d, J = 22.1 Hz, 1H), 6.69 (dd, J = 22.1, 2.9 Hz, 1H), 5.02 (s, 1H), 0.95 (t, J = 8.0 Hz, 9H), 0.74 (q, J = 8.0 Hz, 6H).

^{13}C NMR: (101 MHz, Chloroform-d) δ 156.4, 142.0, 135.5, 133.9, 129.6, 128.0, 9.0, 4.1.

^{29}Si NMR: (80 MHz, Chloroform-d) δ -22.81.

EI-MS m/z (rel. int.): 341 (77), 207 (100%), 133 (15), 105 (28)

EA: $C_{20}H_{28}GeSi$ (370.117): calcd. 65.07; H, 7.65; found C 64.95, H 7.62.

Diethyl(2-(triethylgermyl)vinyl)silane (5ai’)

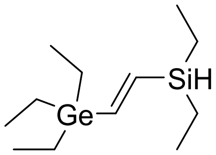

Diethyl(2-(triethylgermyl)vinyl)silane was obtained as a liquid in 83% yield (12.3 mg). The title compound was previously unknown.

^{1}H NMR: (400 MHz, Chloroform-d) δ 6.85 (d, J = 22.2 Hz, 1H), 6.47 (dd, J = 22.2, 2.9 Hz, 1H), 3.87 (h, J = 3.1 Hz, 1H), 1.08 – 0.97 (m, 15H), 0.81 (q, J = 7.7, 1.0 Hz, 6H), 0.72 – 0.63 (m, 4H).

^{13}C NMR: (101 MHz, Chloroform-d) δ 151.9, 144.5, 8.9, 8.1, 4.1, 3.1.

^{29}Si NMR: (79 MHz, Chloroform-d) δ -10.01.

EI-MS m/z (rel. int.): 245 (100%, $[M-Et]^{+}$), 217 (27), 189 (36), 158,9 (19), 133 (67), 102,9 (50), 83 (15), 59 (28)

EA: $C_{12}H_{28}GeSi$ (274.117): calcd. 52.78; H, 10.34; found C 62.81, H 10.30.

(2-(Diphenyl(2-(triethylgermyl)vinyl)silyl)ethyl)trimethylsilane (7a)

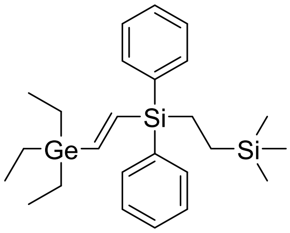

(2-(diphenyl(2-(triethylgermyl)vinyl)silyl)ethyl)trimethylsilane was obtained as a liquid in 82% yield (66.1 mg). The title compound was previously unknown.

^{1}H NMR (600 MHz, Chloroform-d) δ 7.55 (d, J = 7.0 Hz, 4H), 7.41 – 7.37 (m, 5H), 6.92 (d, J = 22.1 Hz, 1H), 6.84 (d, J = 22.4 Hz, 1H), 1.08 (t, J = 8.0 Hz, 10H), 0.92 (t, J = 7.8 Hz, 2H), 0.86 (q, J = 7.9 Hz, 6H), 0.56 (t, 2H), 0.02 (s, 9H).

^{13}C NMR: (151 MHz, Chloroform-d) δ 153.9, 144.1, 145.9, 135.3, 129.1, 127.7, 8.9, 5.2, 4.2, -2.2

^{29}Si NMR: (79 MHz, Chloroform-d) δ 3.25, -14.46.

EI-MS m/z (rel. int.): 441 (100%, $[M-Et]^+$), 369 (23), 183 (65), 135 (76), 104 (49), 73 (90)

EA: $C_{25}H_{40}GeSi_2$ (470.188): calcd. 63.97; H, 8.59; found C 63.99, H 8.55.

(2-Isobutoxyethyl)diphenyl((triisopropylgermyl)ethynyl)silane (7b)

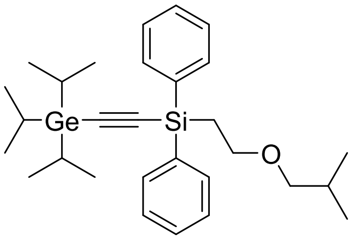

(2-isobutoxyethyl)diphenyl((triisopropylgermyl)ethynyl)silane was obtained as a liquid in 75% yield (32.7 mg). The title compound was previously unknown.

^{1}H NMR: (401 MHz, Chloroform-d) δ 7.73 – 7.69 (m, 4H), 7.41 – 7.37 (m, 6H), 3.68 – 3.63 (m, 2H), 3.14 (d, J = 6.7 Hz, 2H), 1.87 – 1.76 (m, 1H), 1.63 – 1.58 (m, 2H), 1.50 – 1.44 (m, 3H), 1.23 (d, J = 7.3 Hz, 12H), 0.87 (d, J = 6.7 Hz, 6H).

^{13}C NMR: (101 MHz, Chloroform-d) δ 134.7, 129.5, 127.82, 127.75, 116.5, 108.8, 77.4, 67.6, 28.4, 19.9, 19.4, 16.3, 14.9.

^{29}Si NMR: (79 MHz, Chloroform-d) δ -27.81.

EI-MS m/z (rel. int.): 467 (100%, $[M-iPr]^+$), 367 (93), 280 (38), 207 (59), 183 (30), 150 (47)

EA: $C_{29}H_{44}GeOSi$ (510.237): calcd. 68.38; H, 8.71; found C 68.44, H 8.67.

(2-(dimethyl(phenyl)silyl)ethyl)diphenyl(2-(triethylgermyl)vinyl)silane (&c)

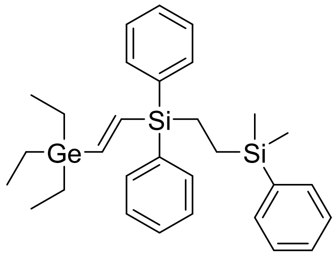

(2-(dimethyl(phenyl)silyl)ethyl)diphenyl(2-(triethylgermyl)vinyl)silane was obtained as a liquid in 84% yield (78.8 mg). The title compound was previously unknown.

^{1}H NMR: (400 MHz, Chloroform-d) δ 7.37 – 7.31 (m, 5H), 7.22 – 7.17 (m, 10H), 6.73 (d, J = 22.3 Hz, 1H), 6.65 (d, J = 22.3 Hz, 1H), 0.89 (t, J = 6.5, 5.1 Hz, 9H), 0.87 – 0.80 (m, 4H), 0.73 – 0.63 (m, 7H), 0.11 (d, J = 6.2 Hz, 6H).

^{13}C NMR: (101 MHz, Chloroform-d) δ 154.2, 144.0, 135.8, 135.3, 135.0, 133.7, 129.2, 128.9, 127.82, 127.77, 9.1, 4.2, -3.52, -3.55.

^{29}Si NMR: (80 MHz, Chloroform-d) δ -1.20, -14.46.

EI-MS m/z (rel. int.) 503 (60%, $[M\text{-}Et]^{+}$), 477 (100), 281 (78), 207 (85), 151 (82), 135 (33)

EA: $C_{30}H_{42}GeSi_2$ (532.204): calcd. 67.80; H, 7.97; found C 67.75, H 7.90.

SPECTRA FOR ALL PRODUCTS

Phenyl((triethylgermyl)ethynyl)silane (3aa)

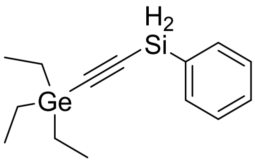

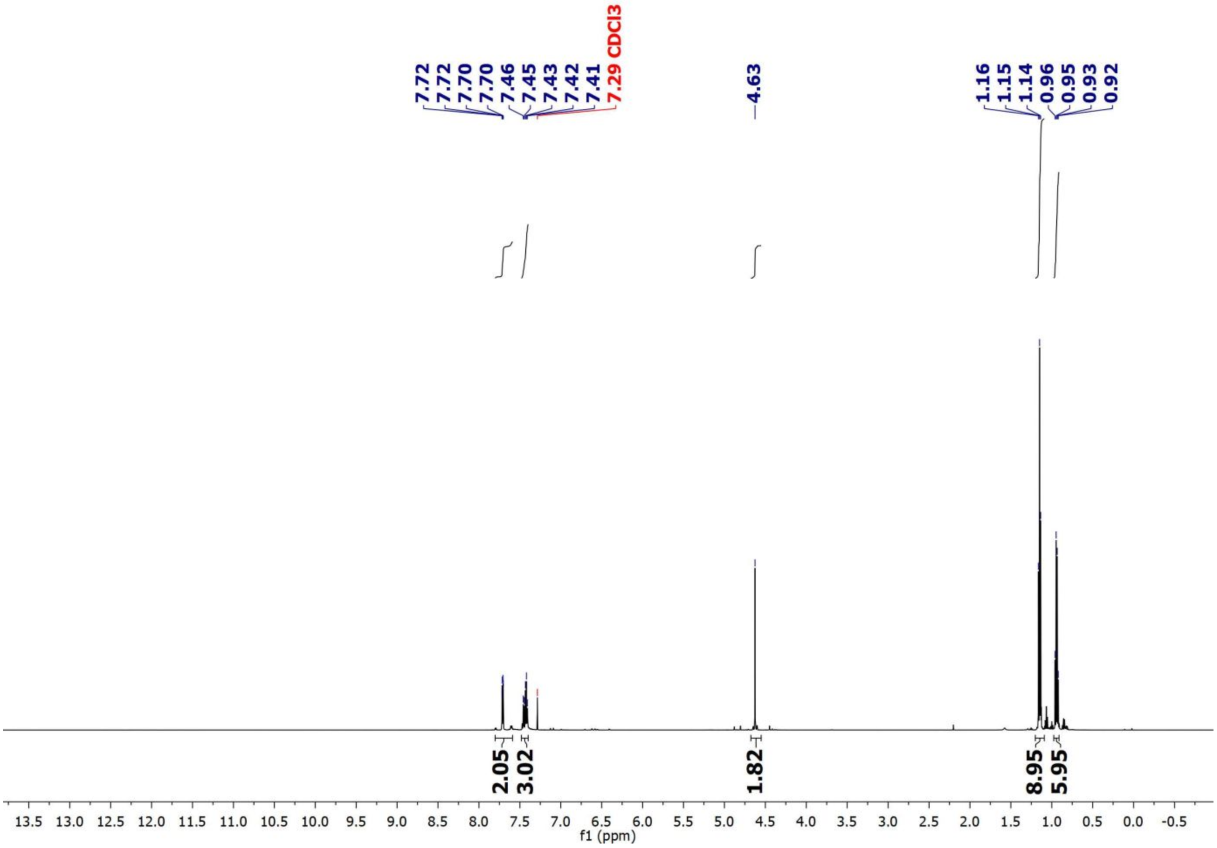

Figure S1. ^{1}H NMR (600 MHz, Chloroform-d) of phenyl((triethylgermyl)ethynyl)silane (**3aa)**

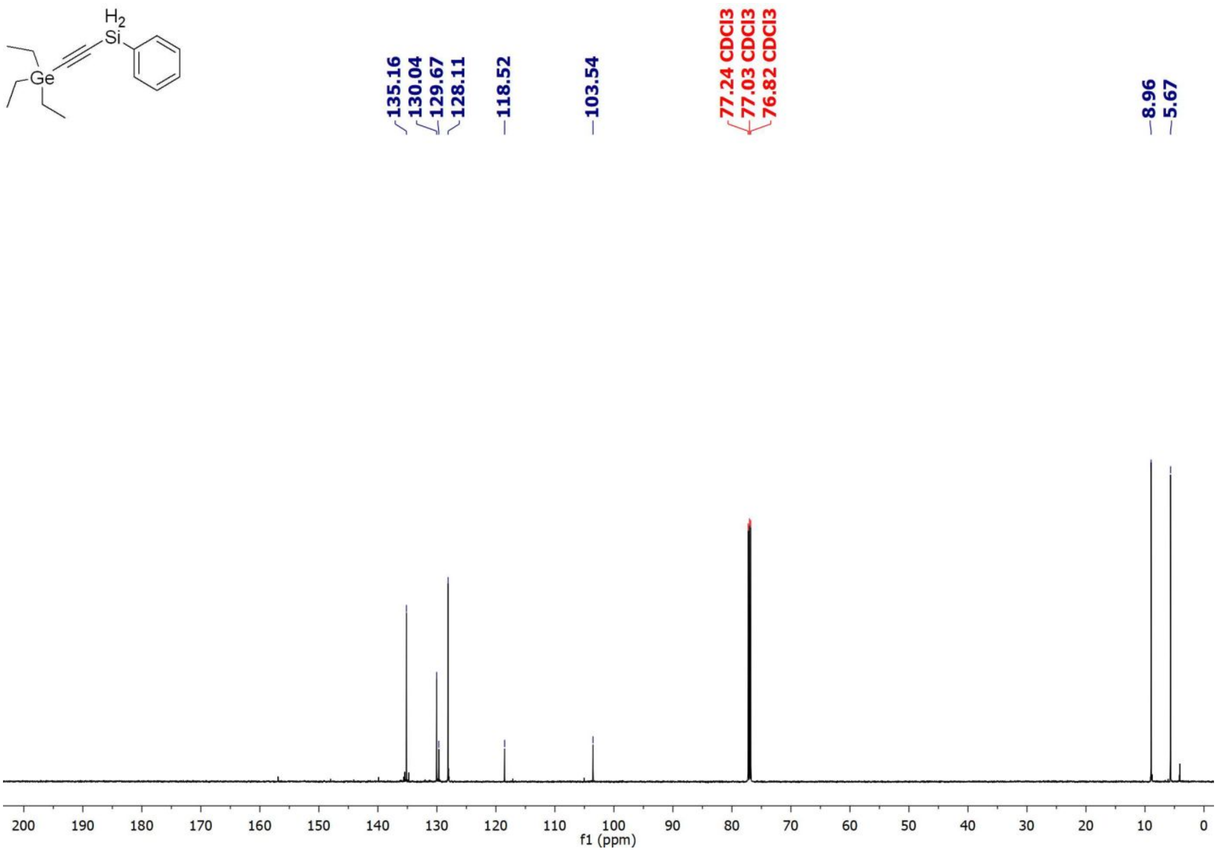

Figure S2. ^{13}C NMR (151 MHz, Chloroform-d) of phenyl((triethylgermyl)ethynyl)silane (**3aa**)

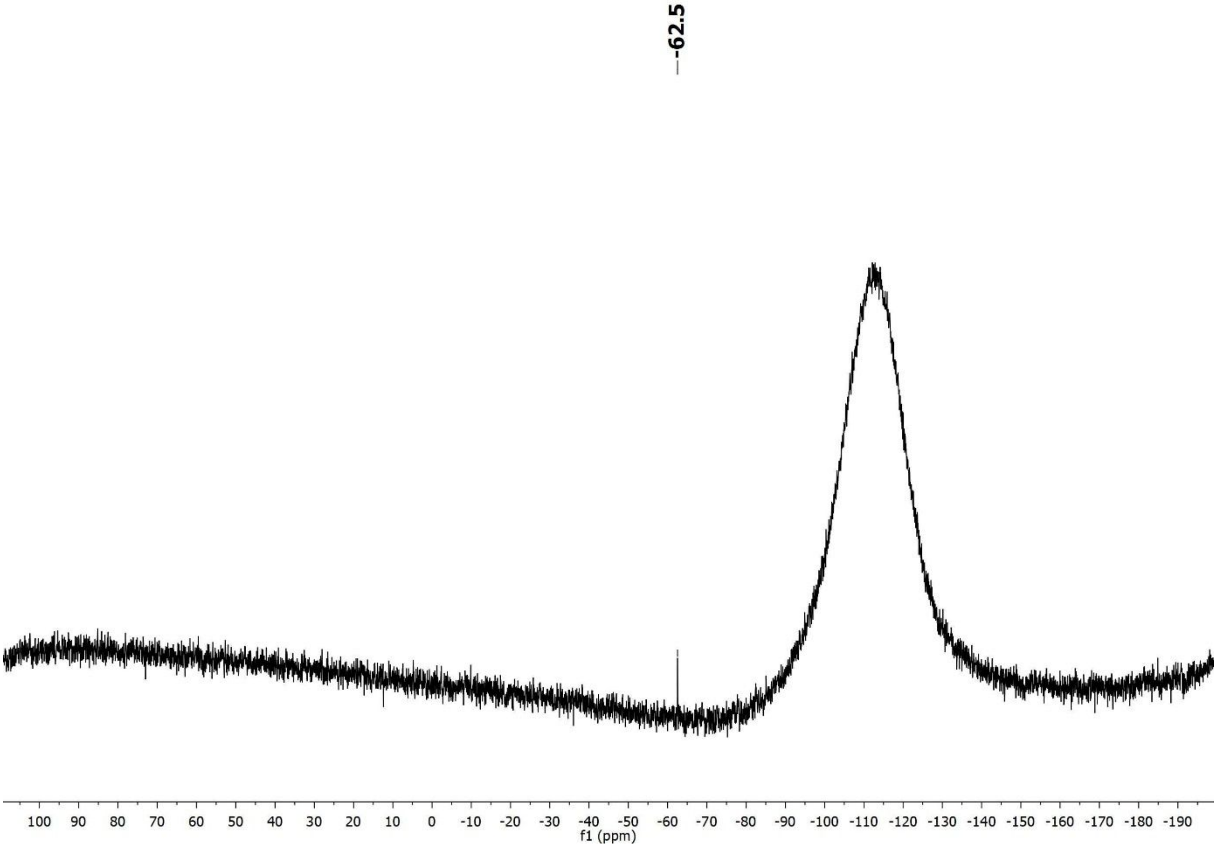

Figure S3. ^{29}Si NMR (79 MHz, Chloroform-d) of phenyl((triethylgermyl)ethynyl)silane (**3aa**)

Hexyl((triethylgermyl)ethynyl)silane (3ab)

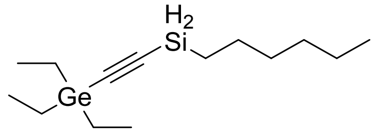

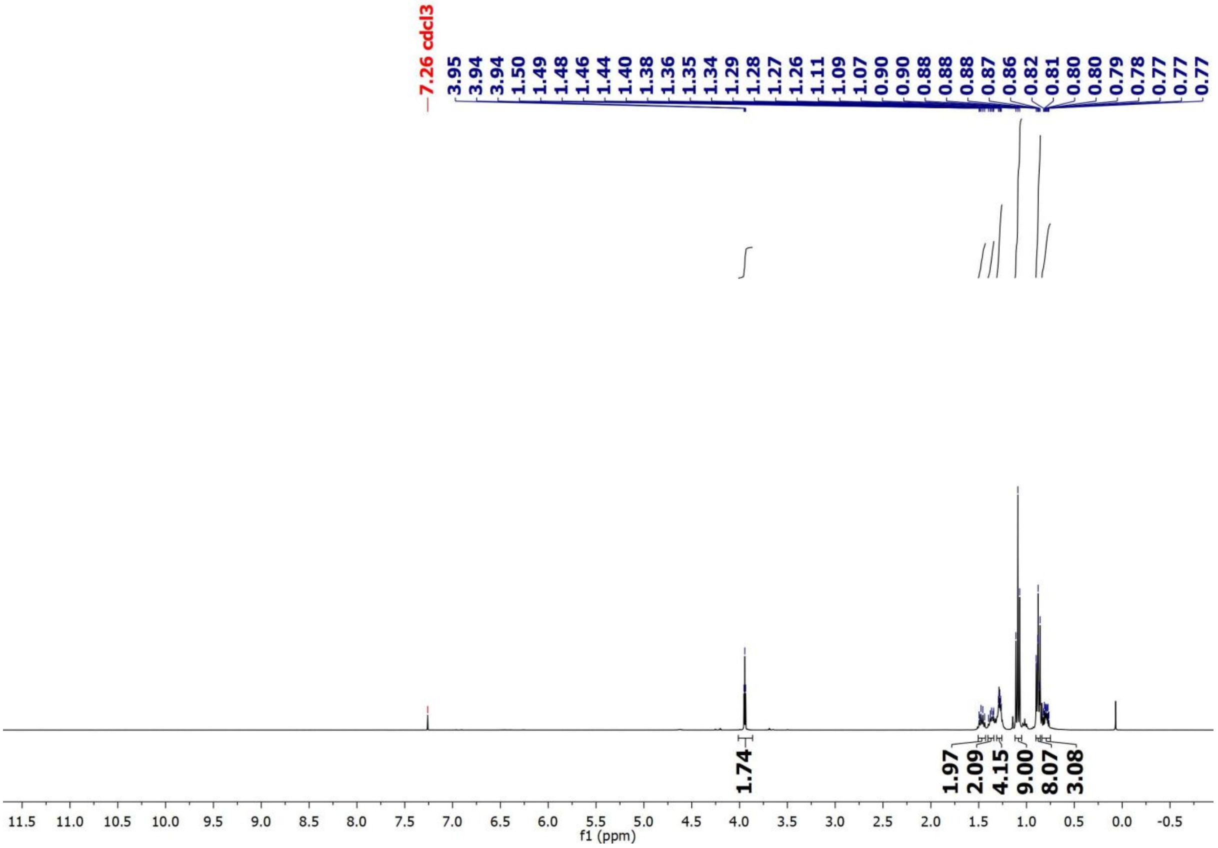

Figure S4. ^1H NMR (400 MHz, Chloroform-d) of hexyl((triethylgermyl)ethynyl)silane (**3ab**)

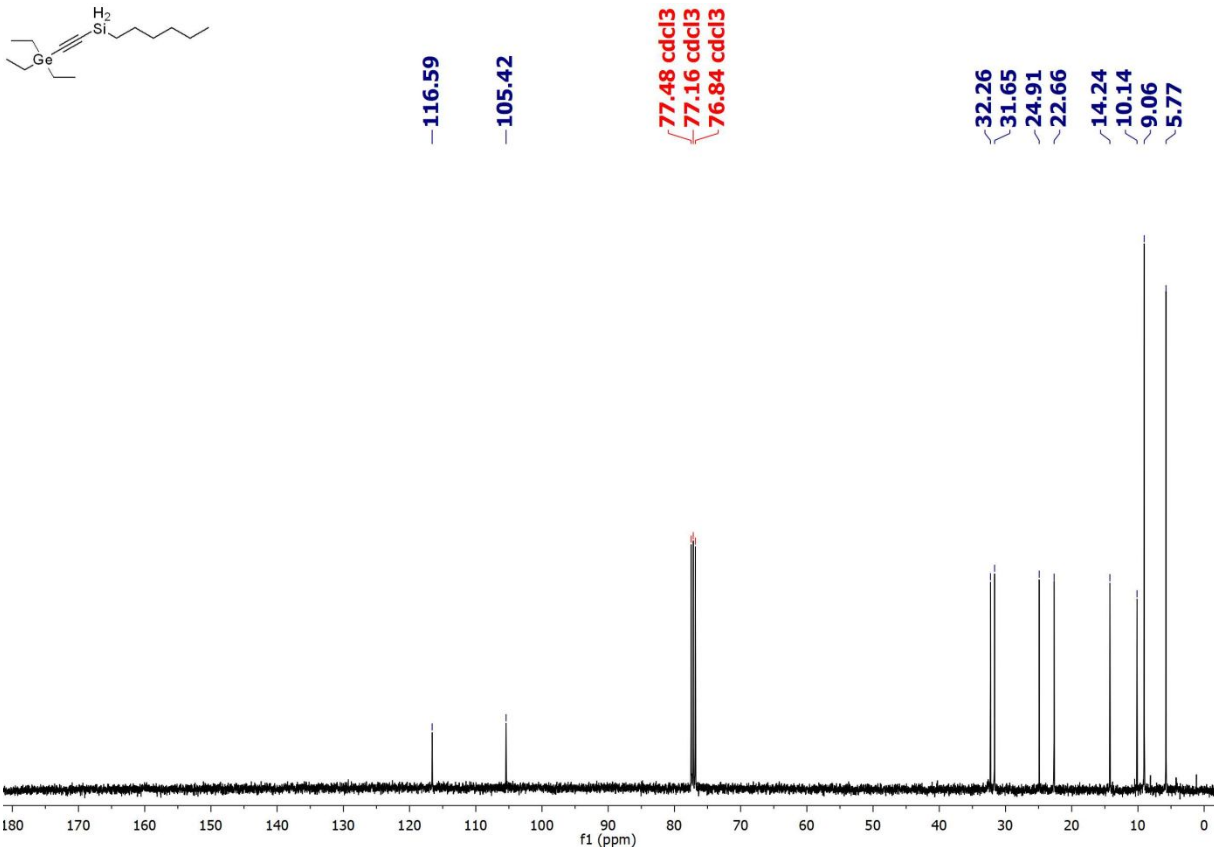

Figure S5. ^{13}C NMR (101 MHz, Chloroform-d) of hexyl((triethylgermyl)ethynyl)silane (**3ab**)

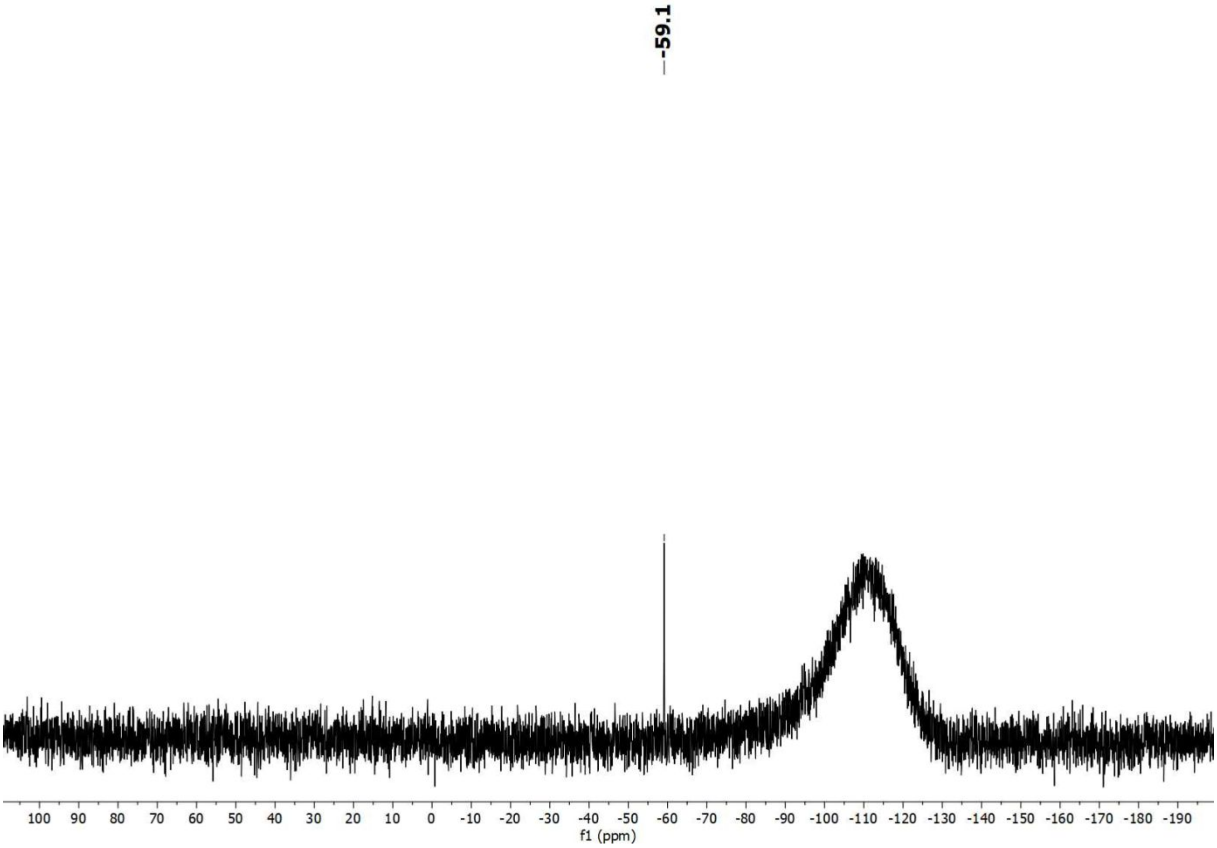

Figure S6. ^{29}Si NMR (79 MHz, Chloroform-d) of hexyl((triethylgermyl)ethynyl)silane (**3ab**)

p-Tolyl((triethylgermyl)ethynyl)silane(3ac)

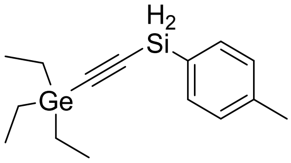

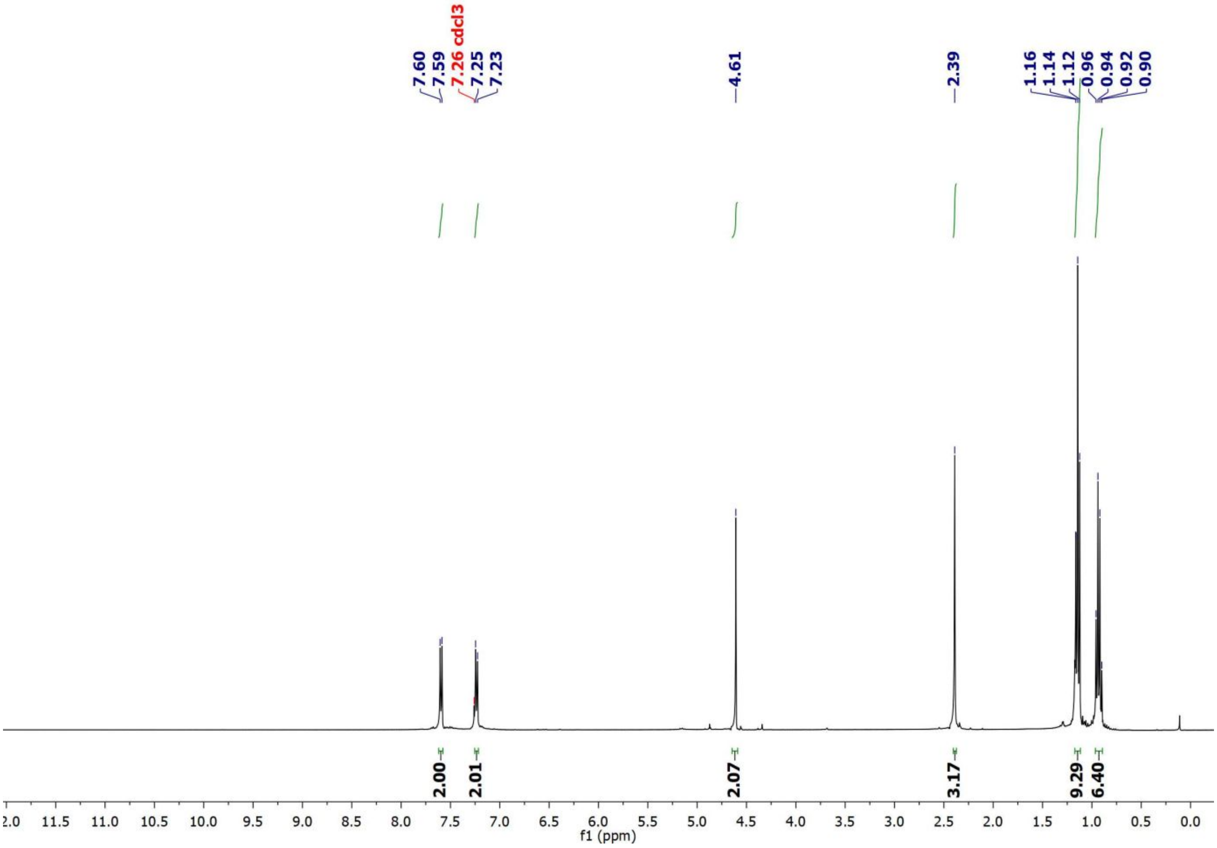

Figure S7. ^{1}H NMR (400 MHz, Chloroform-d) of *p*-tolyl((triethylgermyl)ethynyl)silane **(3ac)**

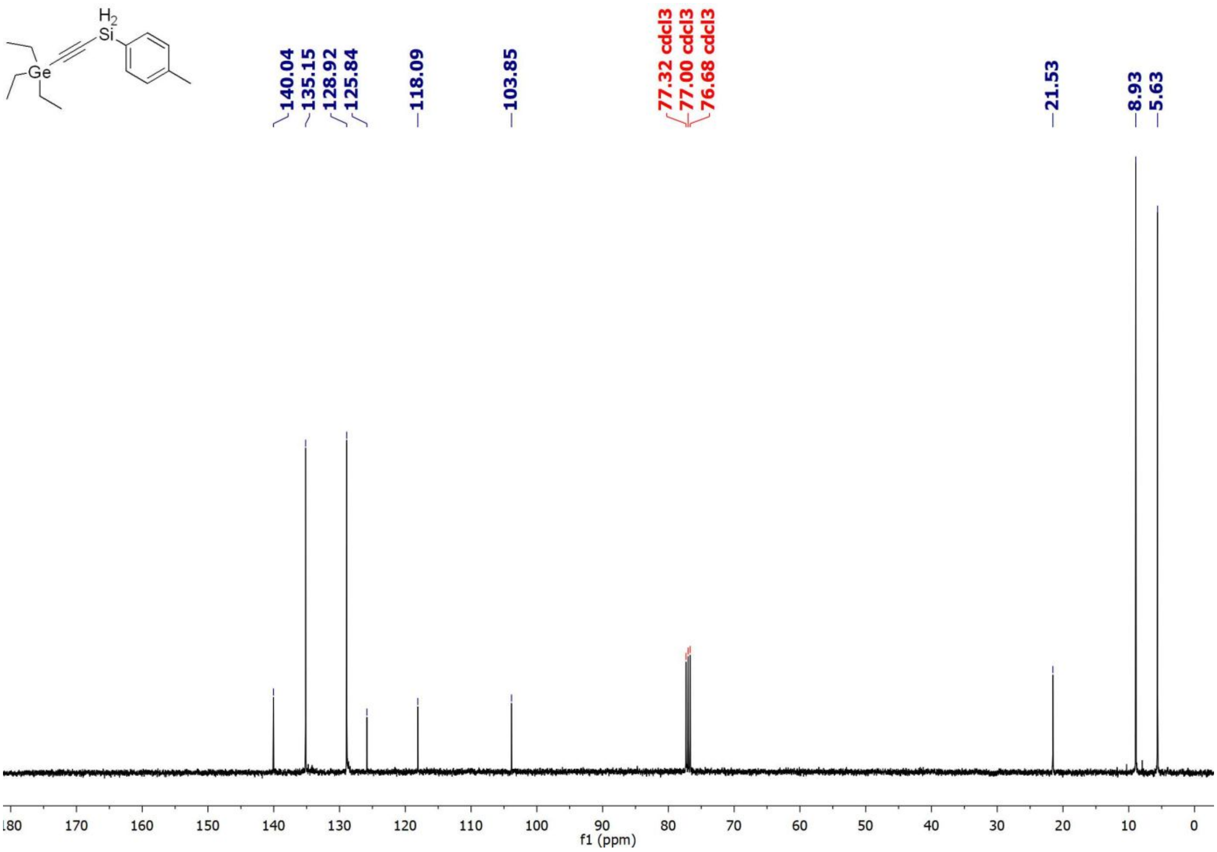

Figure S8. ^{13}C NMR (101 MHz, Chloroform-d) of *p*-tolyl((triethylgermyl)ethynyl)silane **(3ac)**

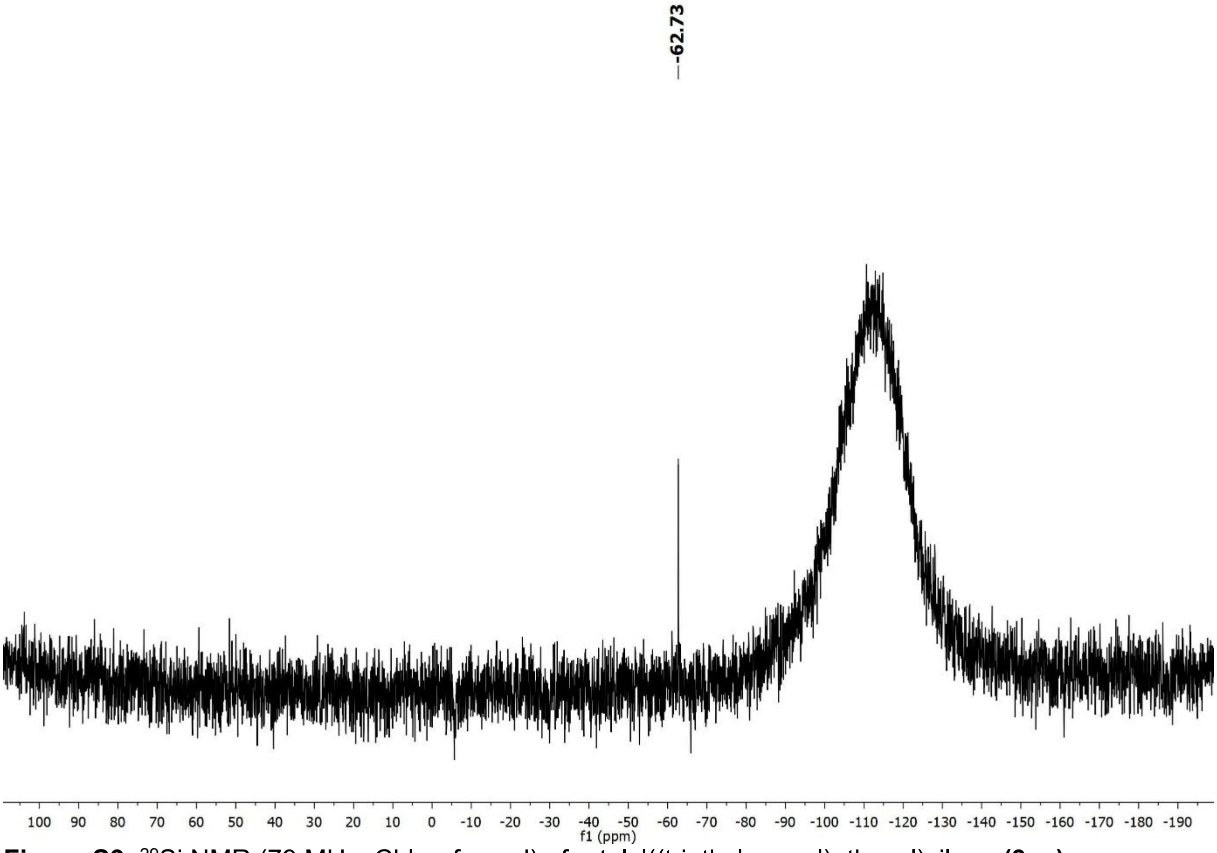

Figure S9. ^{29}Si NMR (79 MHz, Chloroform-d) of *p*-tolyl((triethylgermyl)ethynyl)silane **(3ac)**

Octyl((triethylgermyl)ethynyl)silane (3ad)

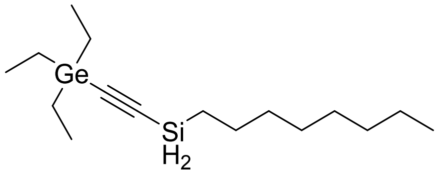

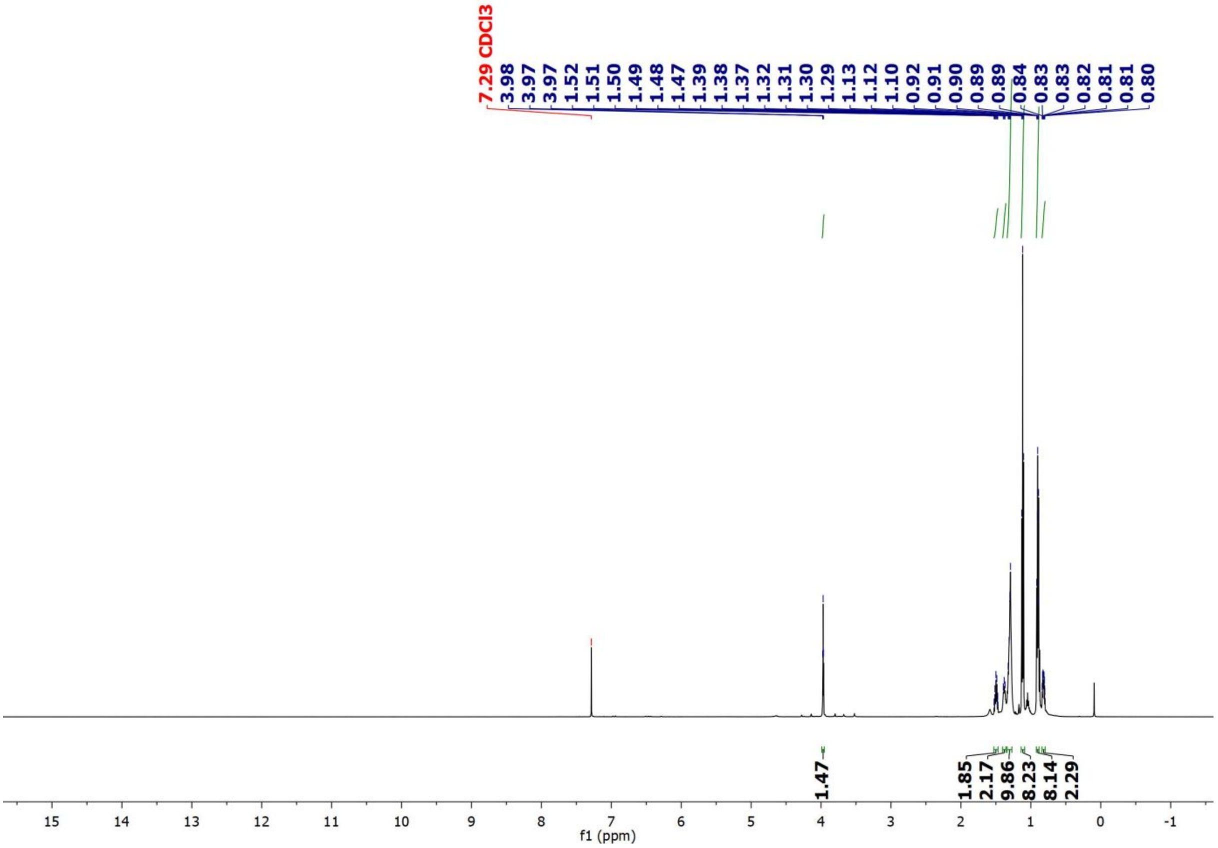

Figure S10. ^{1}H NMR (600 MHz, Chloroform-d) of octyl((triethylgermyl)ethynyl)silane **(3ad)**

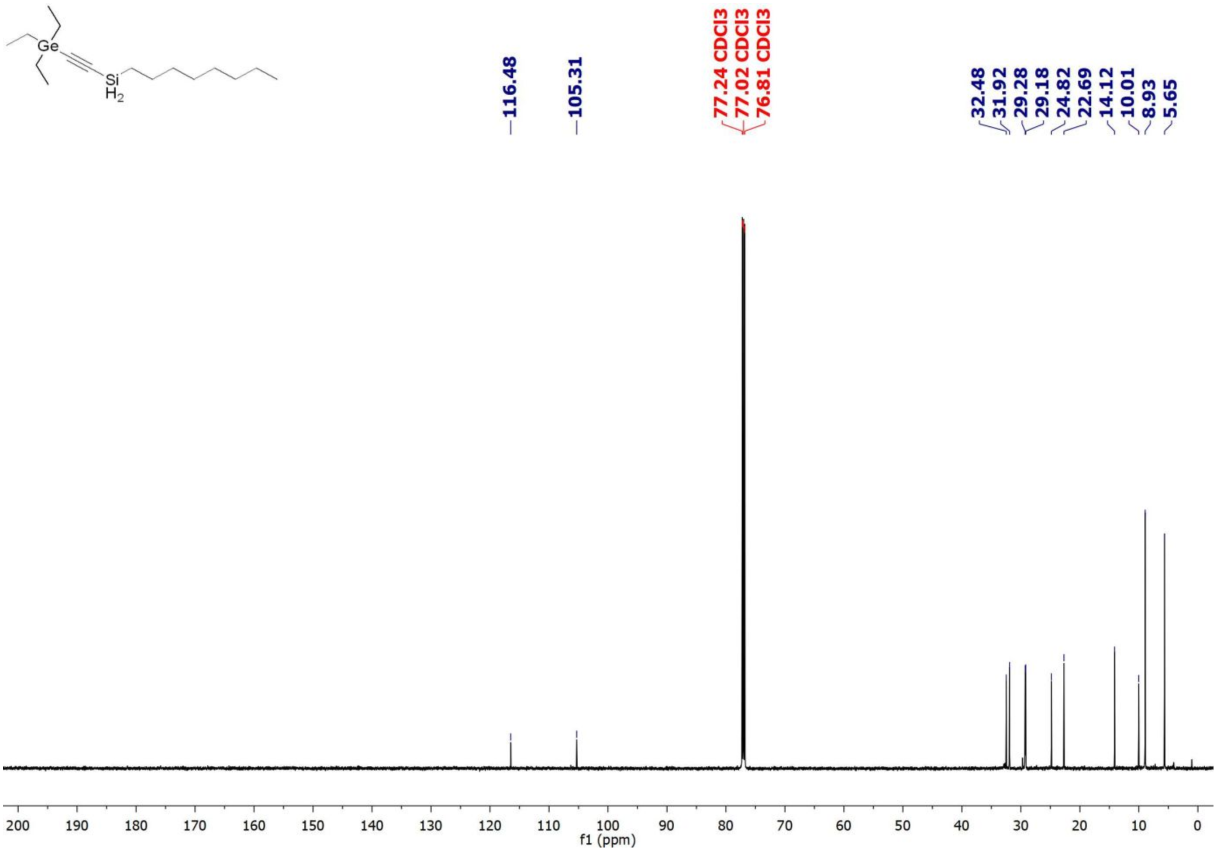

Figure S11. ^{13}C NMR (151 MHz, Chloroform-d) of octyl((triethylgermyl)ethynyl)silane **(3ad)**

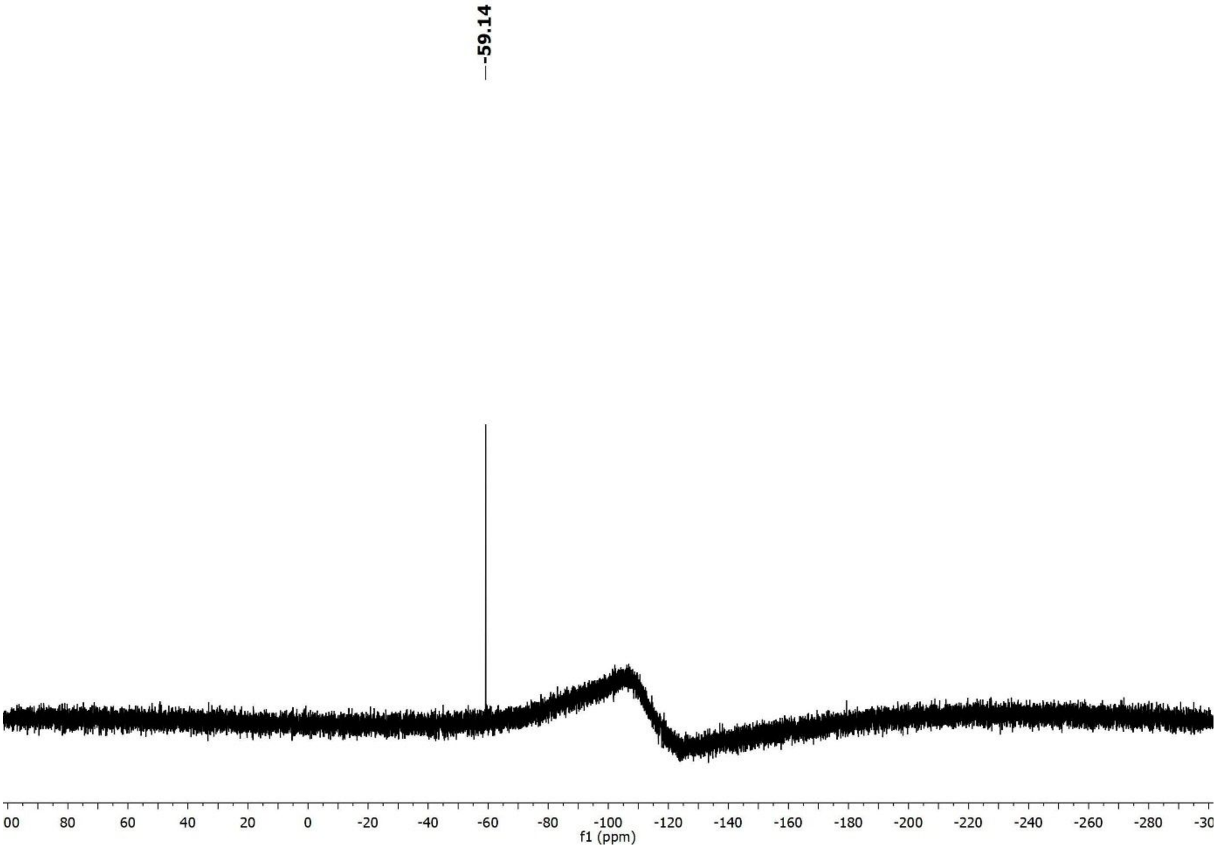

Figure S12. ^{29}Si NMR (119 MHz, Chloroform-d) of octyl((triethylgermyl)ethynyl)silane **(3ad)**

Cyclohexyl((triethylgermyl)ethynyl)silane (3ae)

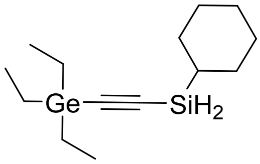

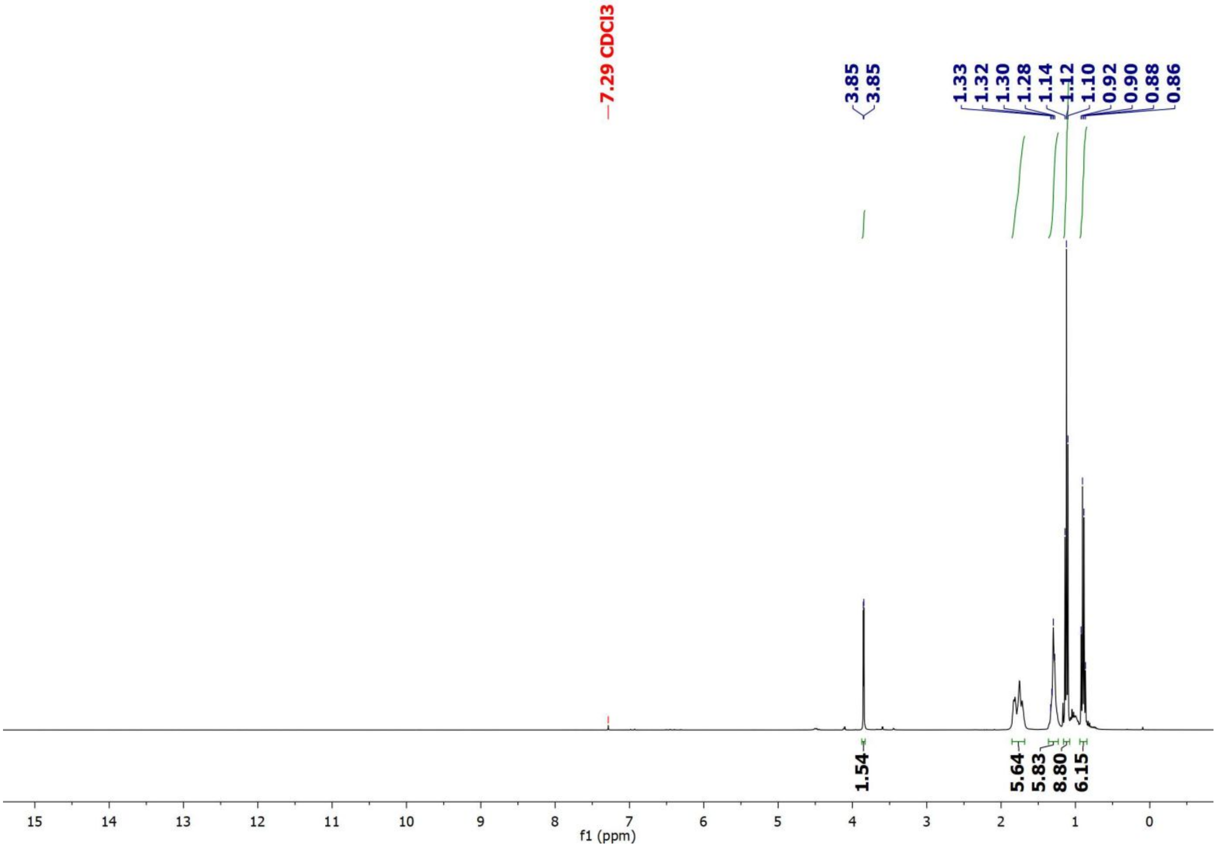

Figure S13. ^{1}H NMR (400 MHz, Chloroform-d) of cyclohexyl((triethylgermyl)ethynyl)silane **(3ae)**

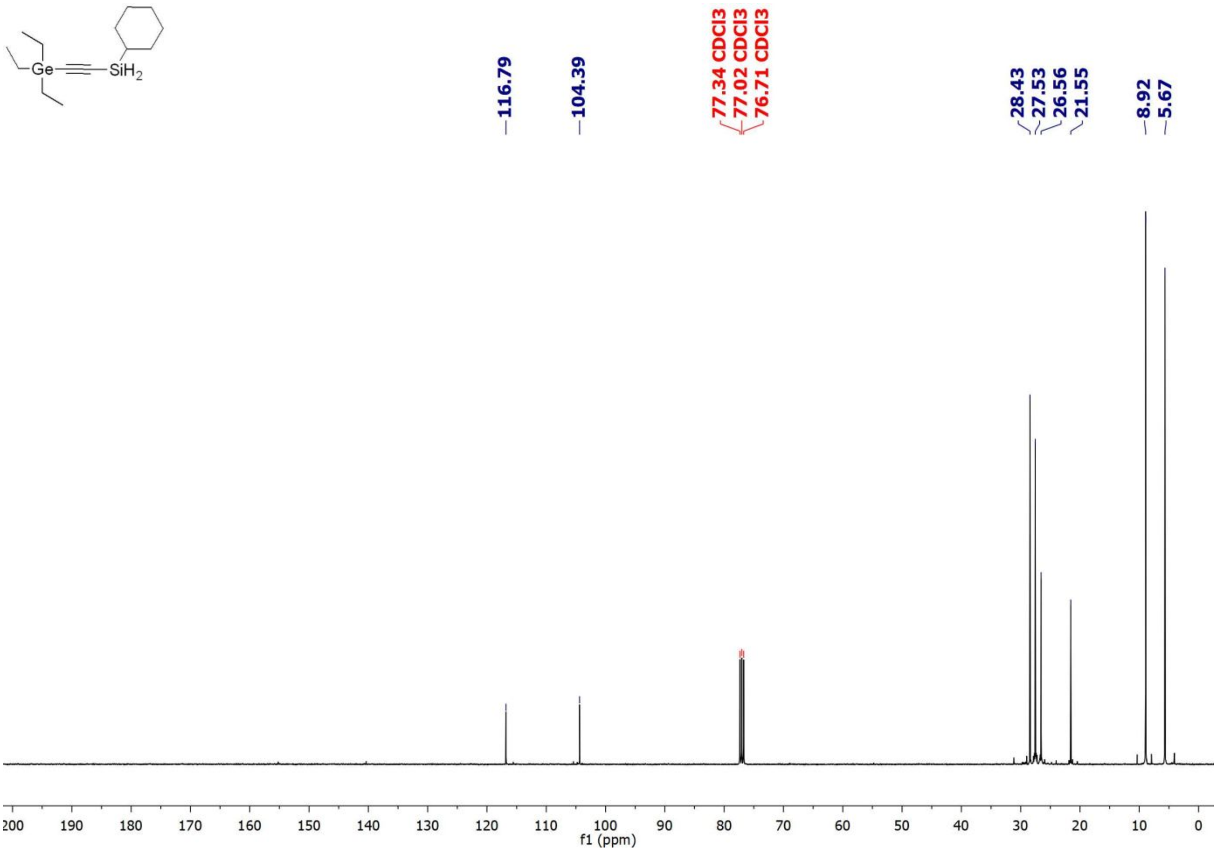

Figure S14. ^{13}C NMR (101 MHz, Chloroform-d) of cyclohexyl((triethylgermyl)ethynyl)silane **(3ae)**

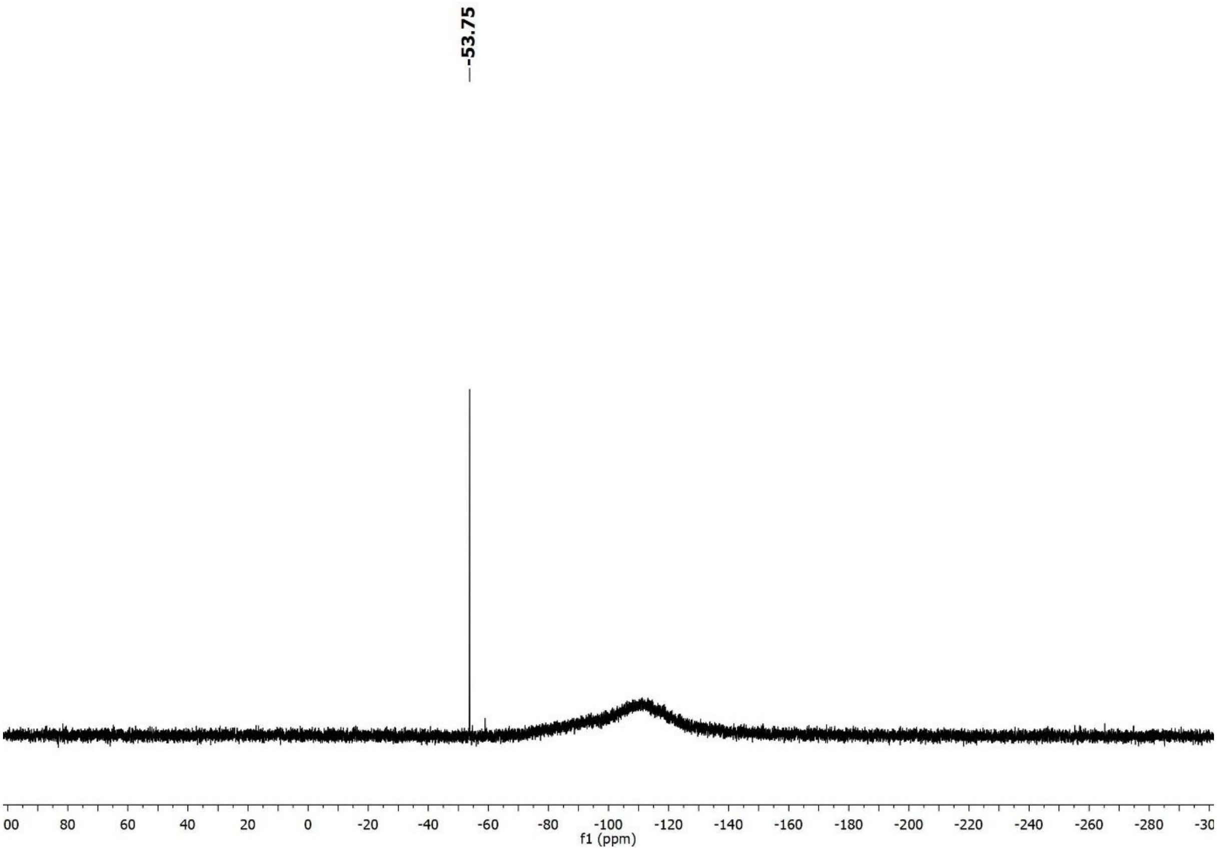

Figure S15. ^{29}Si NMR (79 MHz, Chloroform-d) of cyclohexyl((triethylgermyl)ethynyl)silane **(3ae)**

Phenyl((triisopropylgermyl)ethynyl)silane (3ba)

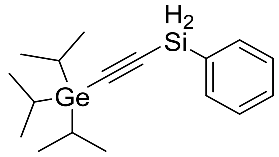

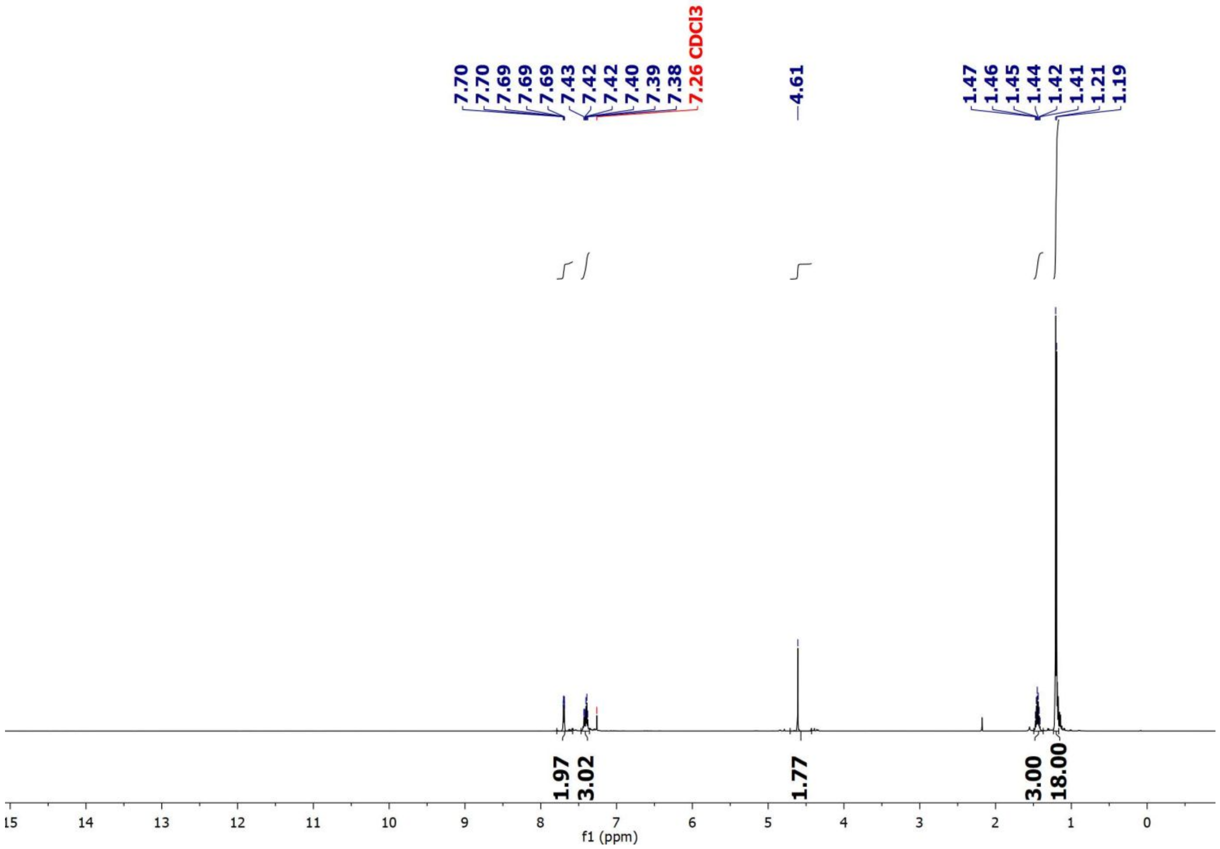

Figure S16. ^{1}H NMR (600 MHz, Chloroform-d) of phenyl((triisopropylgermyl)ethynyl)silane (**3ba**)

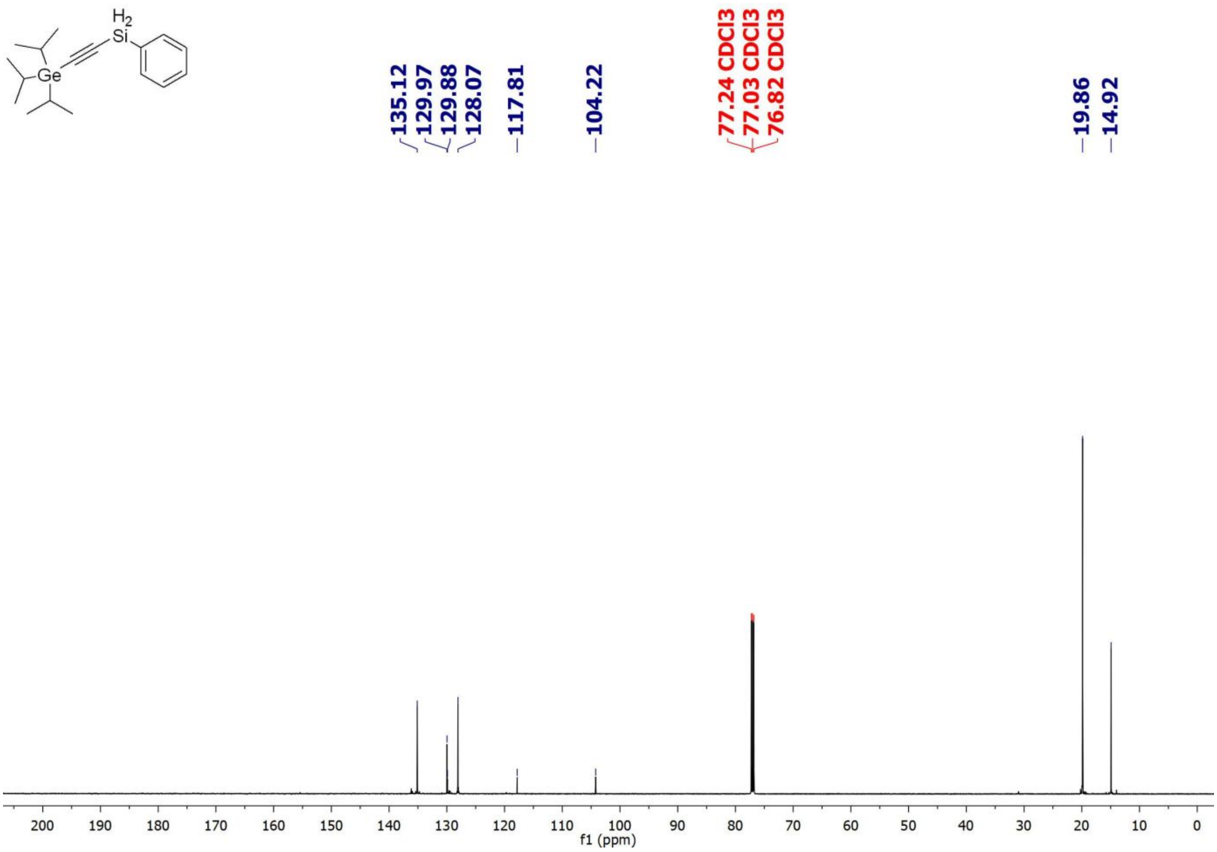

Figure S17. ^{13}C NMR (151 MHz, Chloroform-d) of phenyl((triisopropylgermyl)ethynyl)silane (**3ba)**

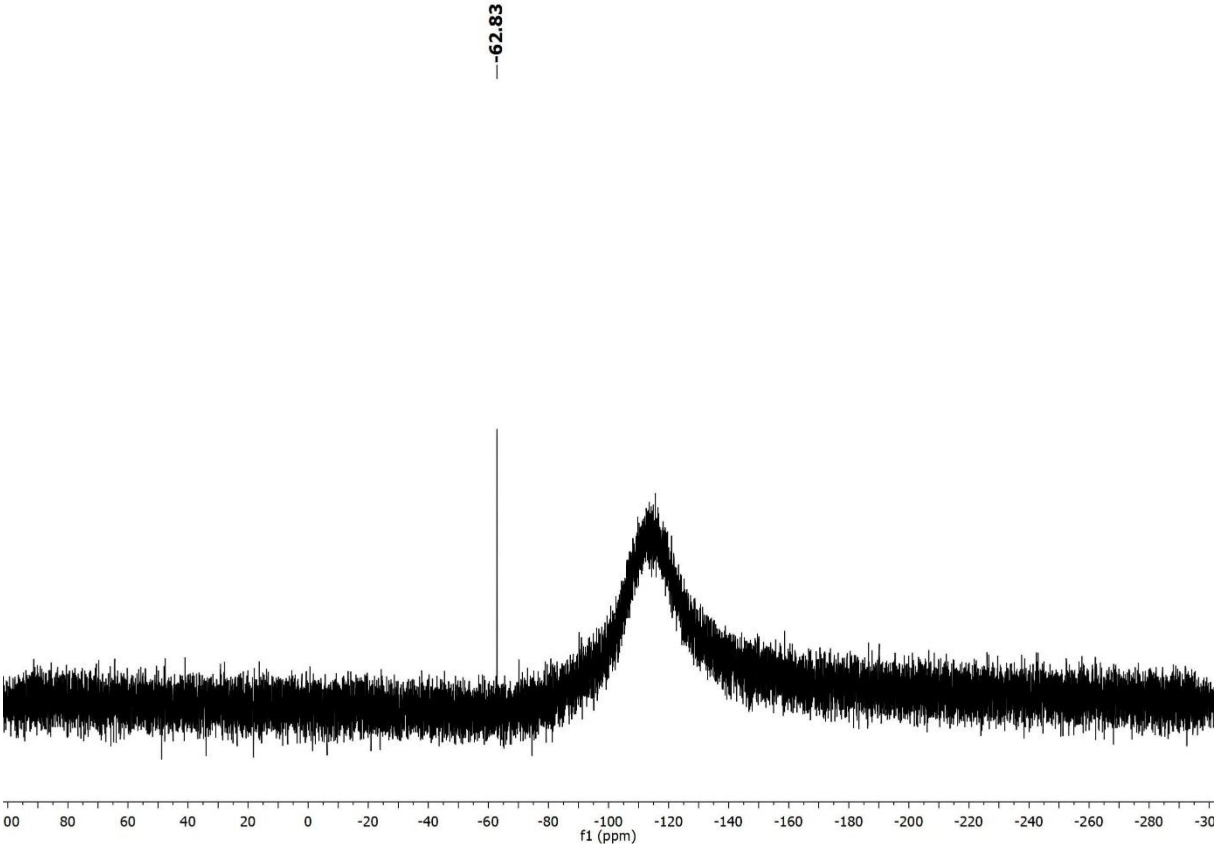

Figure S18. ^{29}Si NMR (119 MHz, Chloroform-d) of phenyl((triisopropylgermyl)ethynyl)silane (**3ba)**

Hexyl((triisopropylgermyl)ethynyl)silane (3bb)

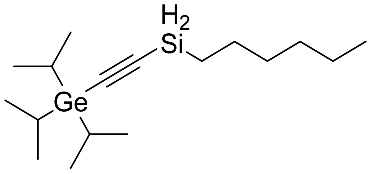

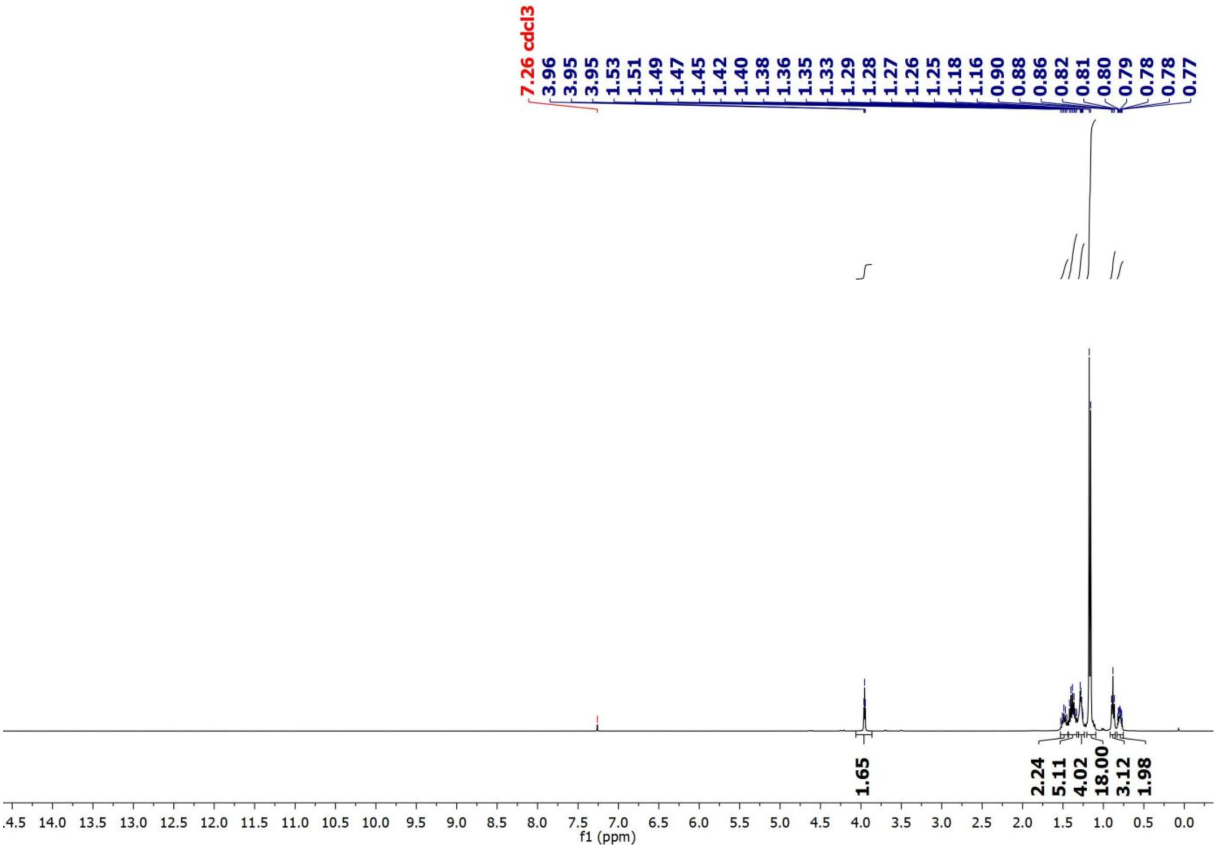

Figure S19. ^{1}H NMR (400 MHz, Chloroform-d) of hexyl((triisopropylgermyl)ethynyl)silane (**3bb**)

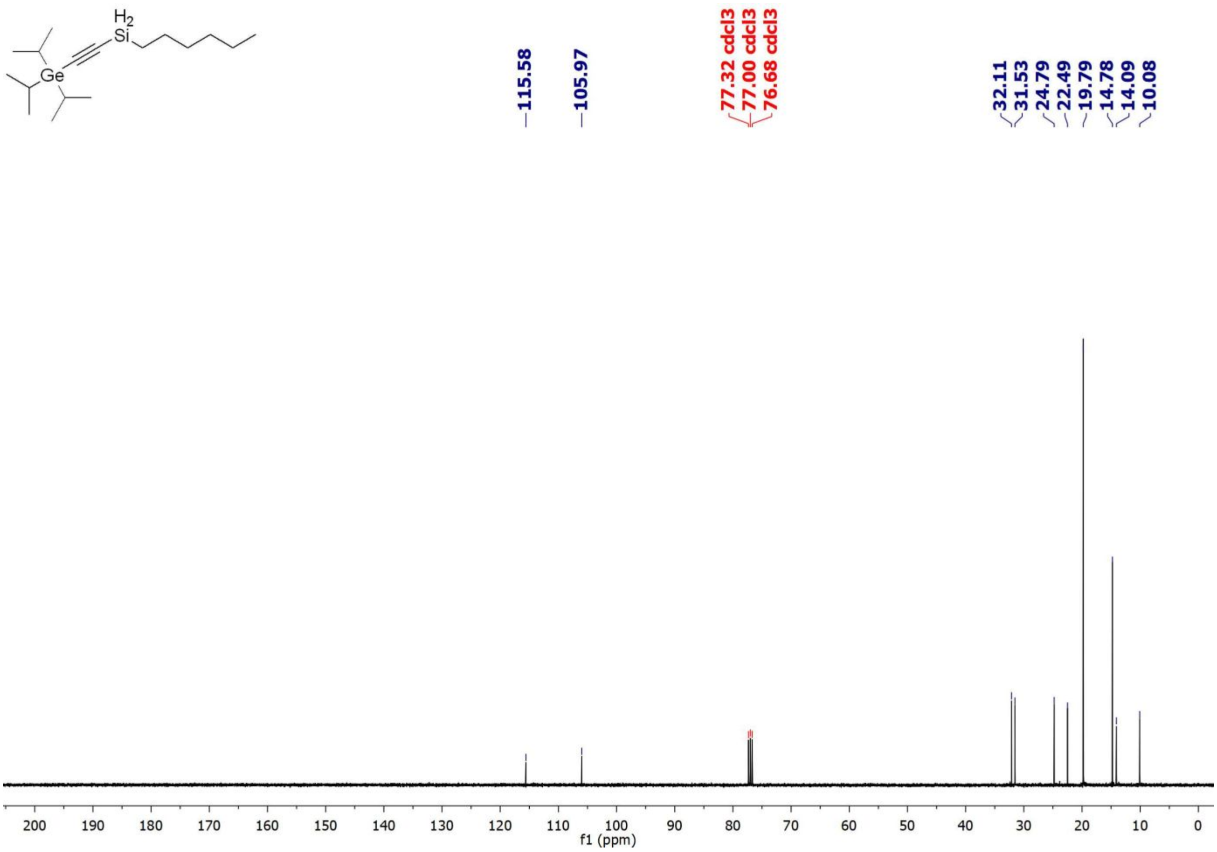

Figure S20. ^{13}C NMR (101 MHz, Chloroform-d) of hexyl((triisopropylgermyl)ethynyl)silane (**3bb**)

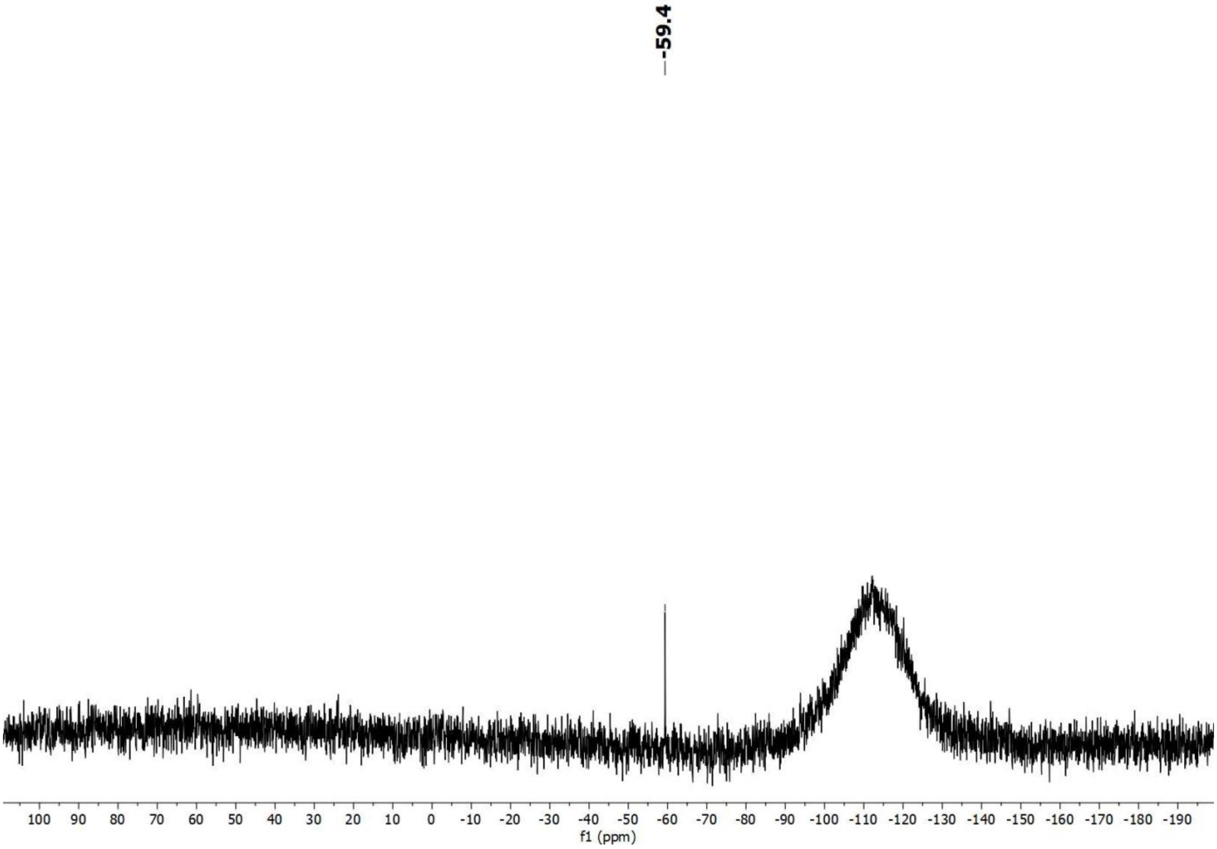

Figure S21. ^{29}Si NMR (79 MHz, Chloroform-d) of hexyl((triisopropylgermyl)ethynyl)silane (**3bb**)

p-Tolyl((triisopropylgermyl)ethynyl)silane (3bc)

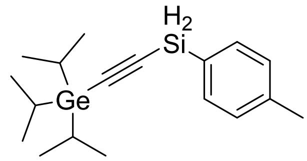

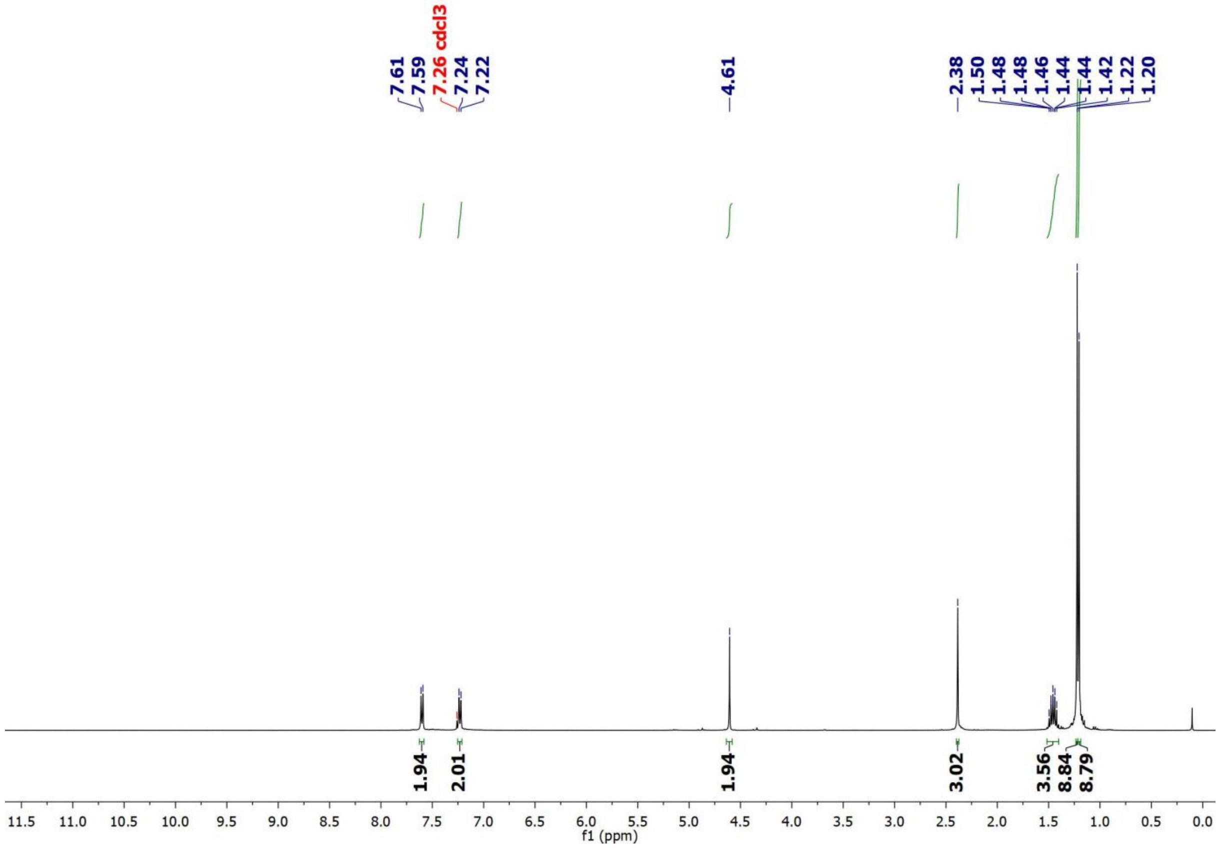

Figure S22. ^{1}H NMR (400 MHz, Chloroform-d) of *p*-tolyl((triisopropylgermyl)ethynyl)silane **(3bc)**

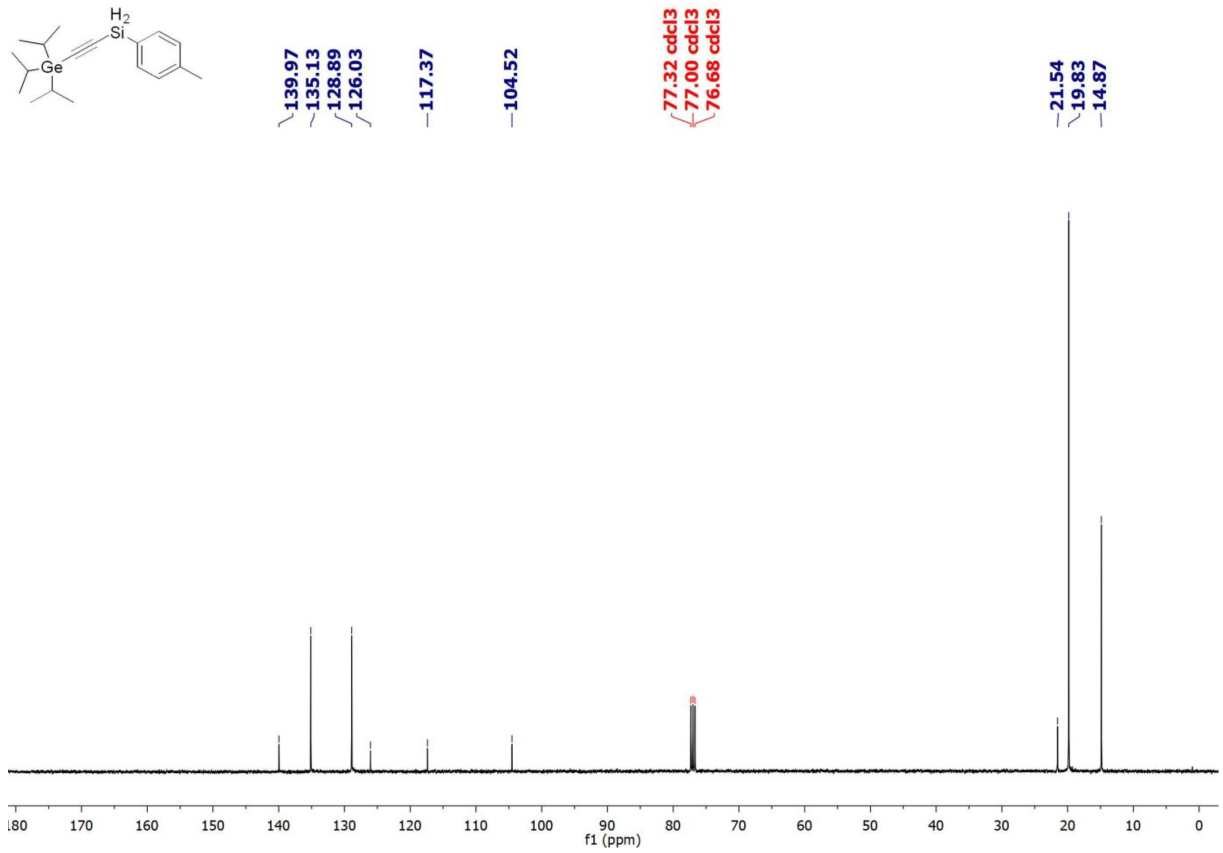

Figure S23. ^{13}C NMR (101 MHz, Chloroform-d) of *p*-tolyl((triisopropylgermyl)ethynyl)silane **(3bc)**

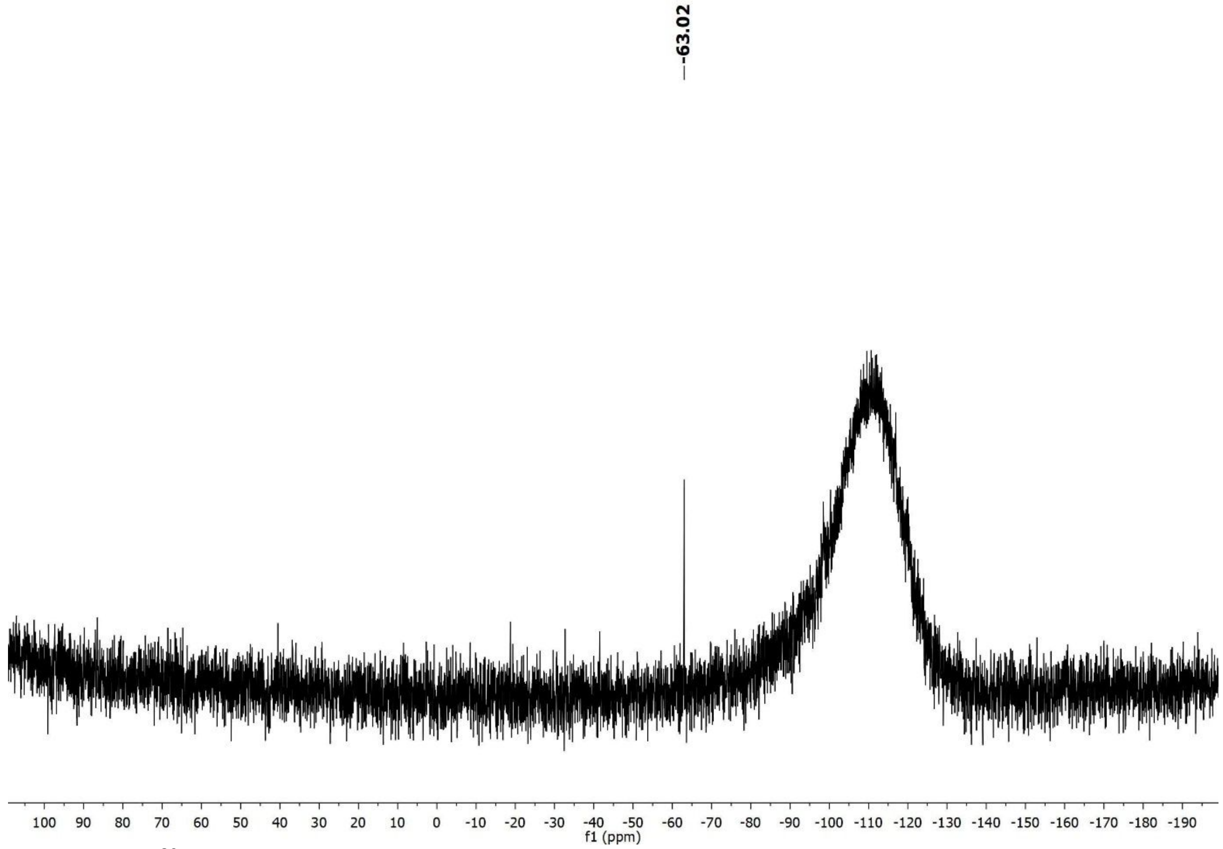

Figure S24. ^{29}Si NMR (79 MHz, Chloroform-d) of *p*-tolyl((triisopropylgermyl)ethynyl)silane **(3bc)**

Octyl((triisopropylgermyl)ethynyl)silane (3bd)

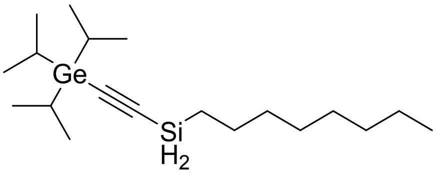

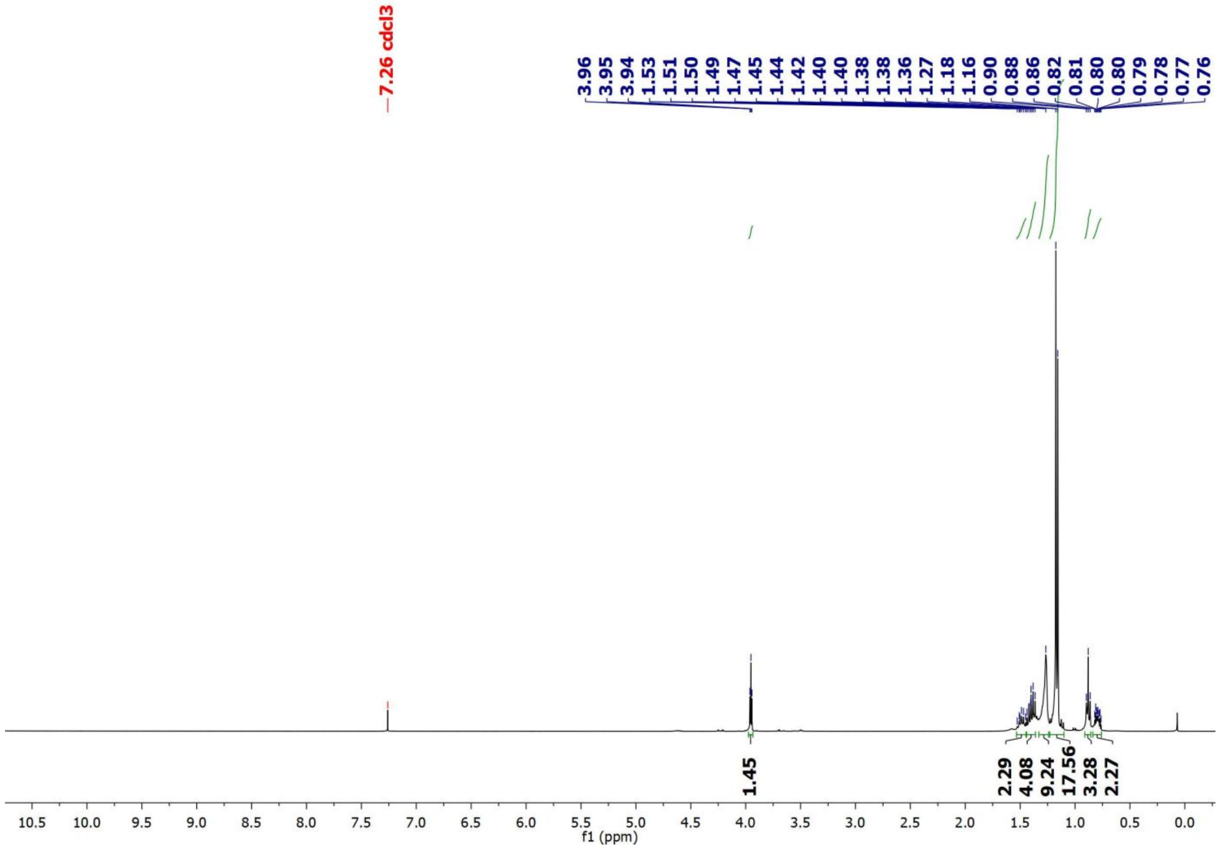

Figure S25. ^{1}H NMR (400 MHz, Chloroform-d) of octyl((triisopropylgermyl)ethynyl)silane **(3bd)**

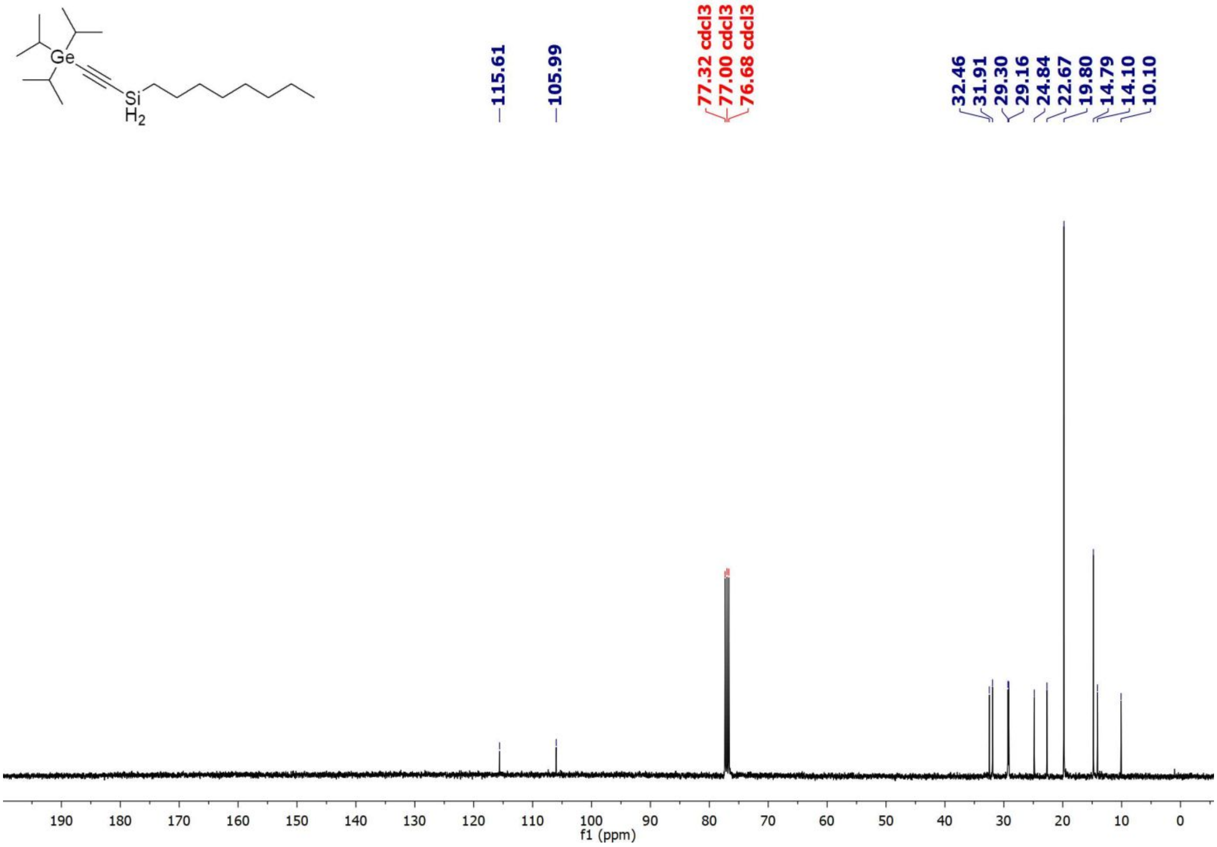

Figure S26. ^{13}C NMR (101 MHz, Chloroform-d) of octyl((triisopropylgermyl)ethynyl)silane **(3bd)**

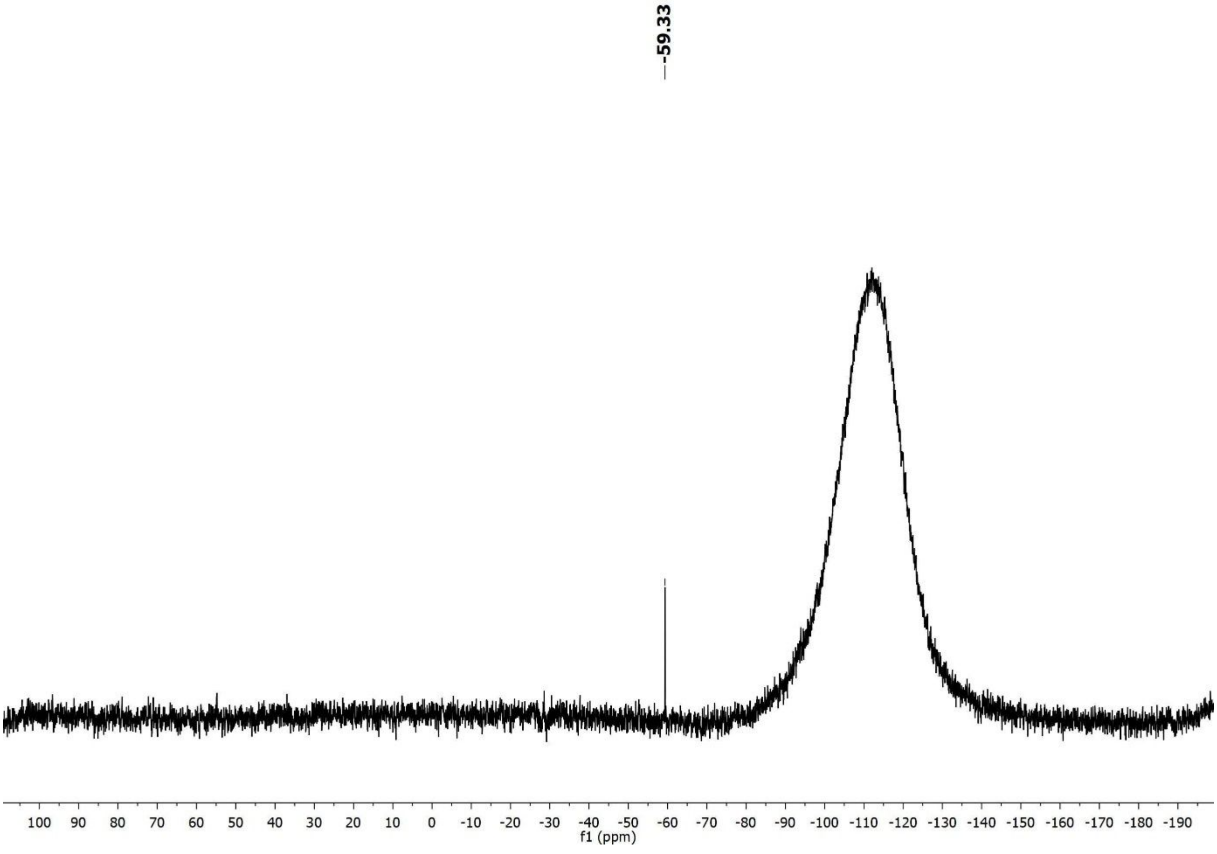

Figure S27. ^{29}Si NMR (79 MHz, Chloroform-d) of octyl((triisopropylgermyl)ethynyl)silane **(3bd)**

Cyclohexyl((triisopropylgermyl)ethynyl)silane (3be)

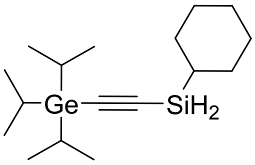

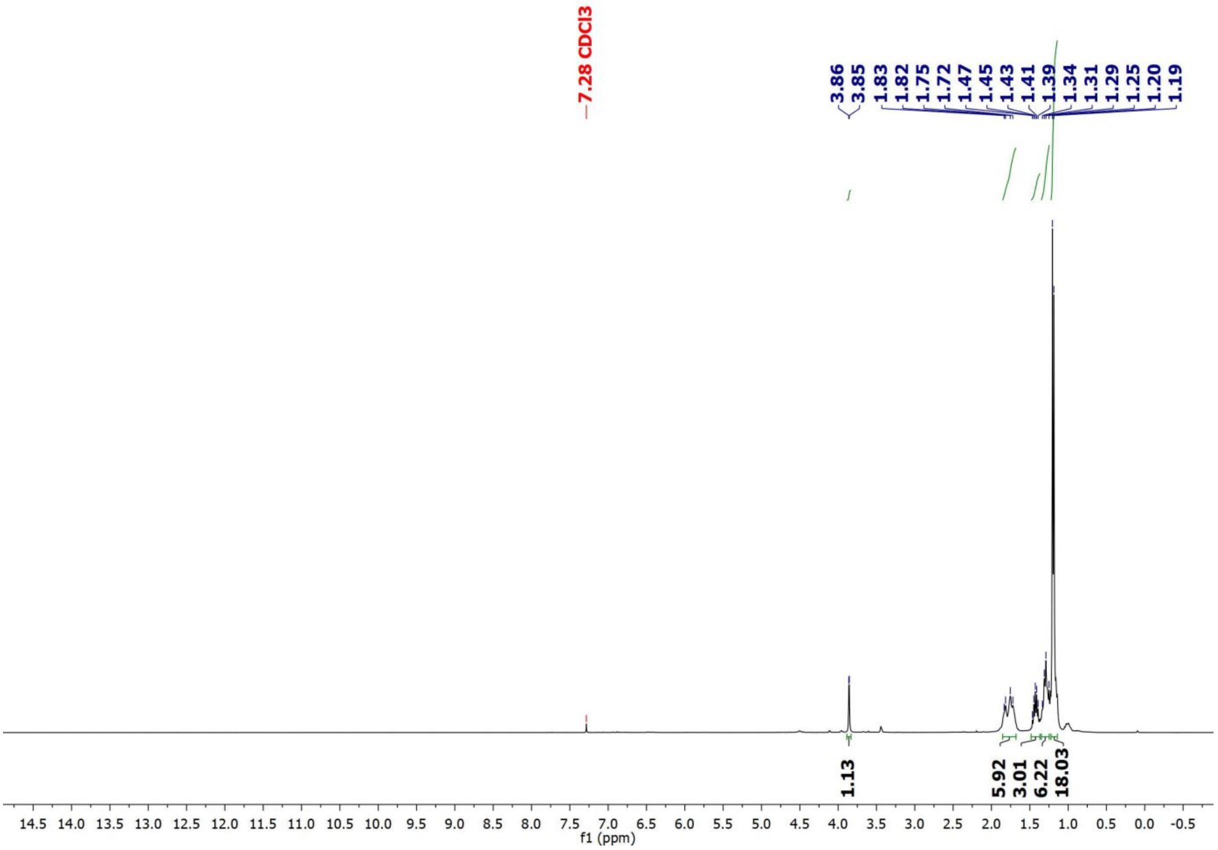

Figure S28. ^{1}H NMR (400 MHz, Chloroform-d) of cyclohexyl((triisopropylgermyl)ethynyl)silane **(3be)**

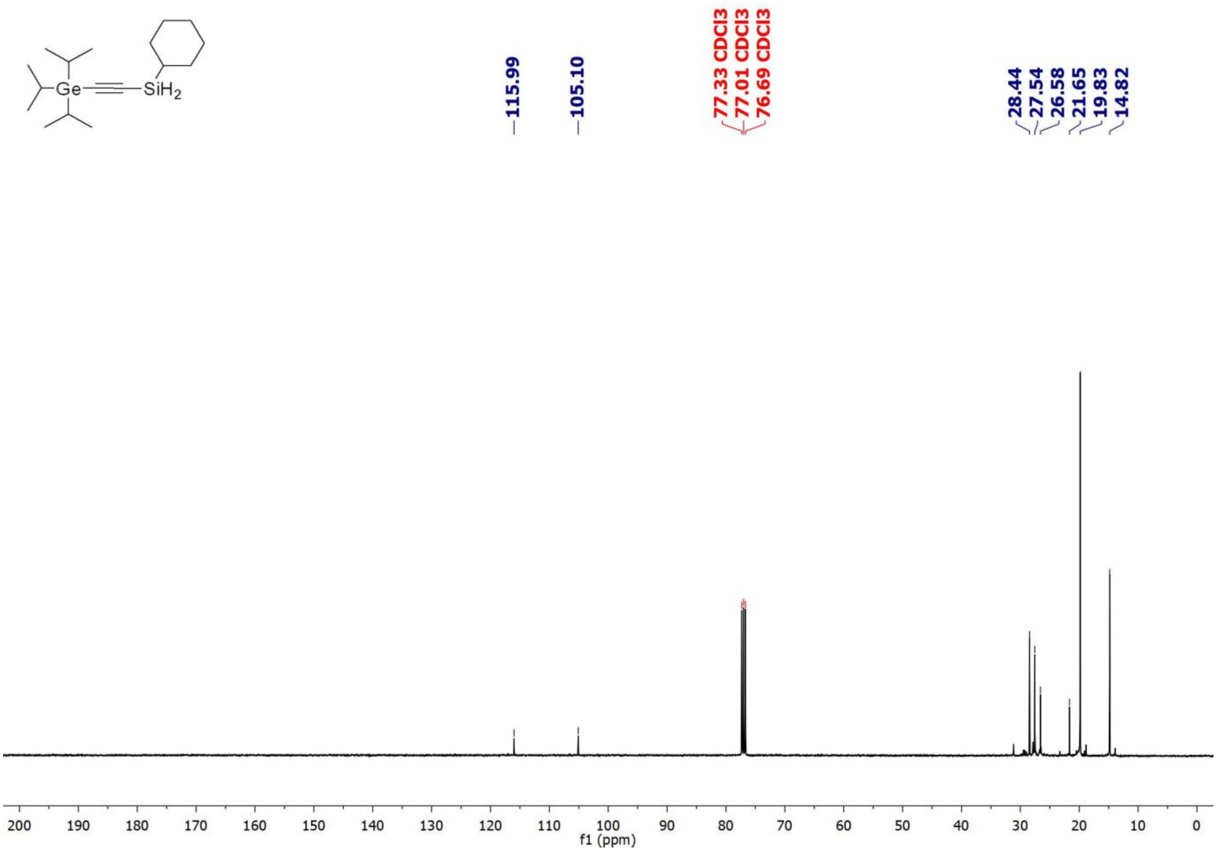

Figure S29. ^{13}C NMR (101 MHz, Chloroform-d) of cyclohexyl((triisopropylgermyl)ethynyl)silane **(3be)**

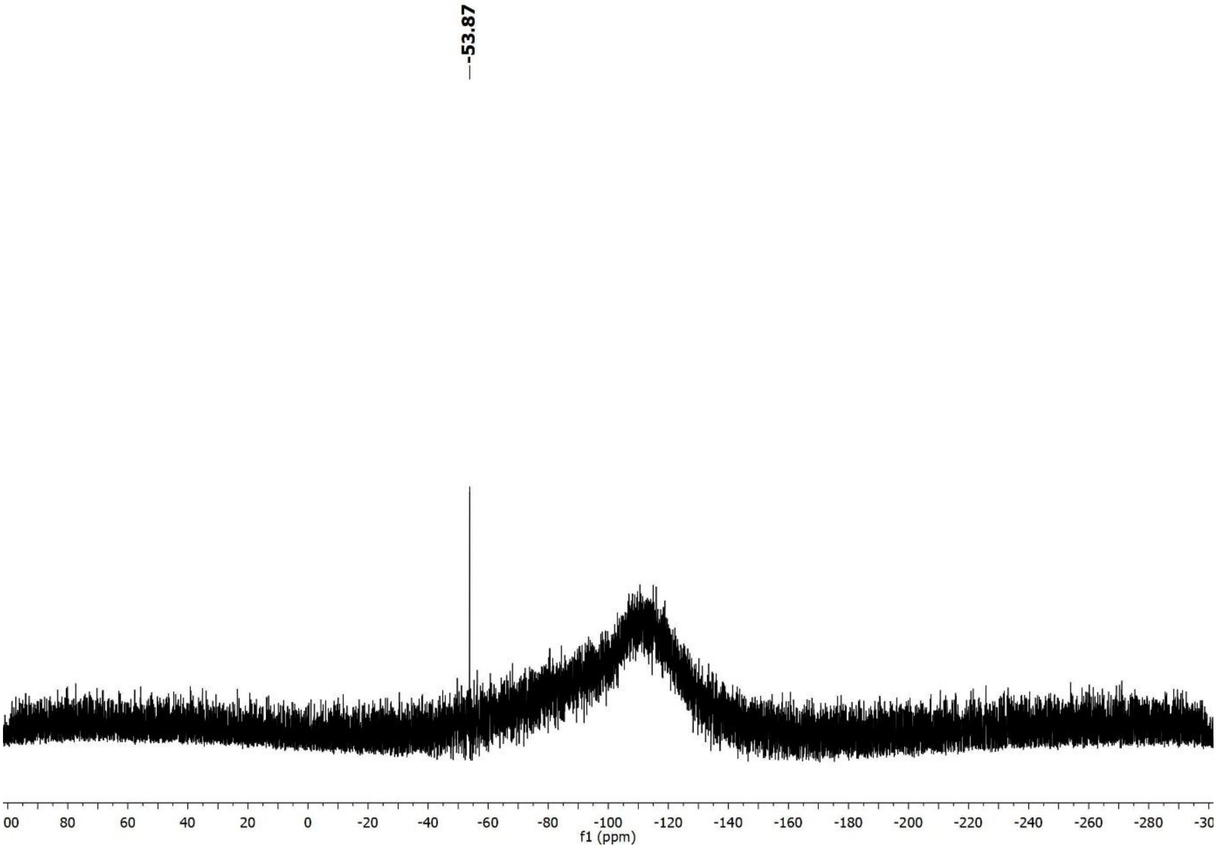

Figure S30. ^{29}Si NMR (79 MHz, Chloroform-d) of cyclohexyl((triisopropylgermyl)ethynyl)silane **(3be)**

Butyl((triisopropylgermyl)ethynyl)silane (3bf)

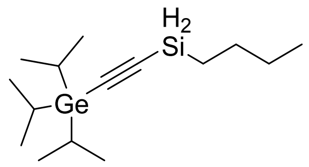

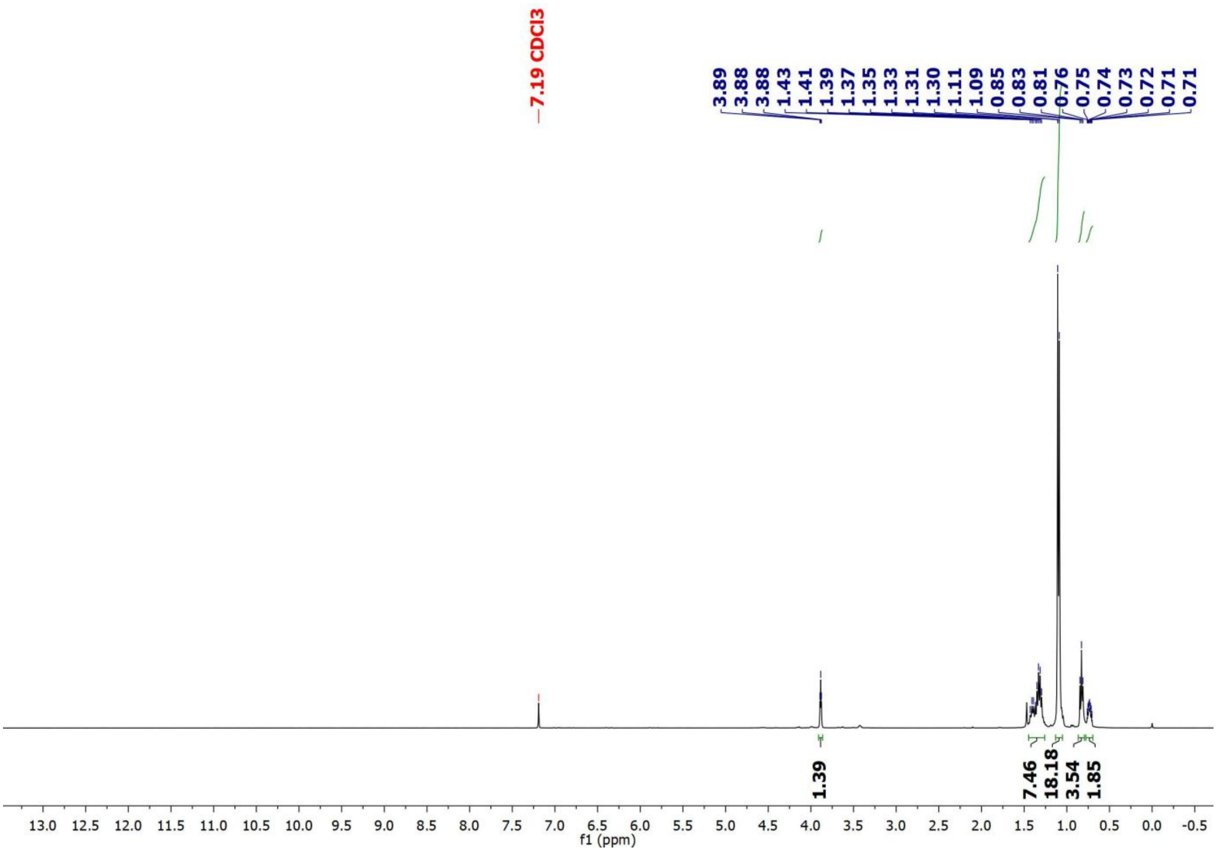

Figure S31. ^{1}H NMR (400 MHz, Chloroform-d) of butyl((triisopropylgermyl)ethynyl)silane **(3bf)**

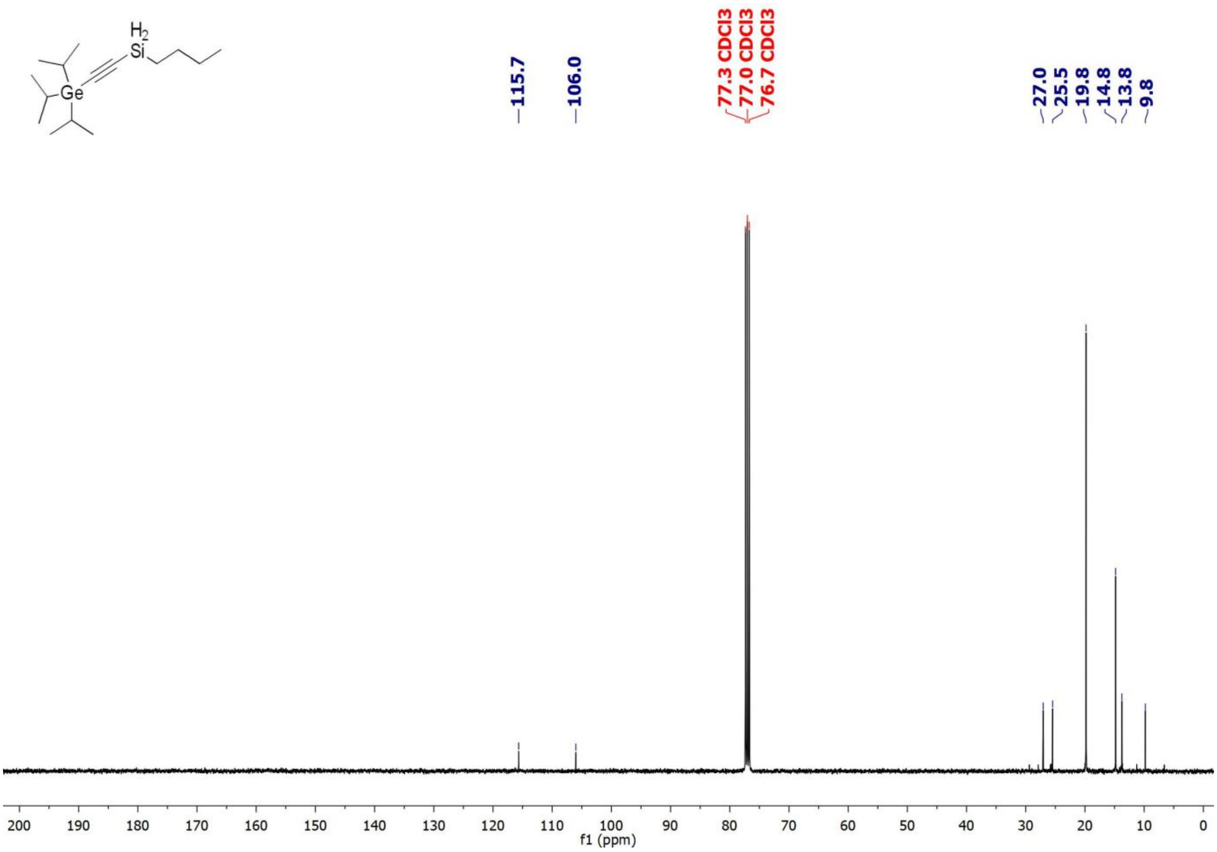

Figure S32. ^{13}C NMR (101 MHz, Chloroform-d) of butyl((triisopropylgermyl)ethynyl)silane **(3bf)**

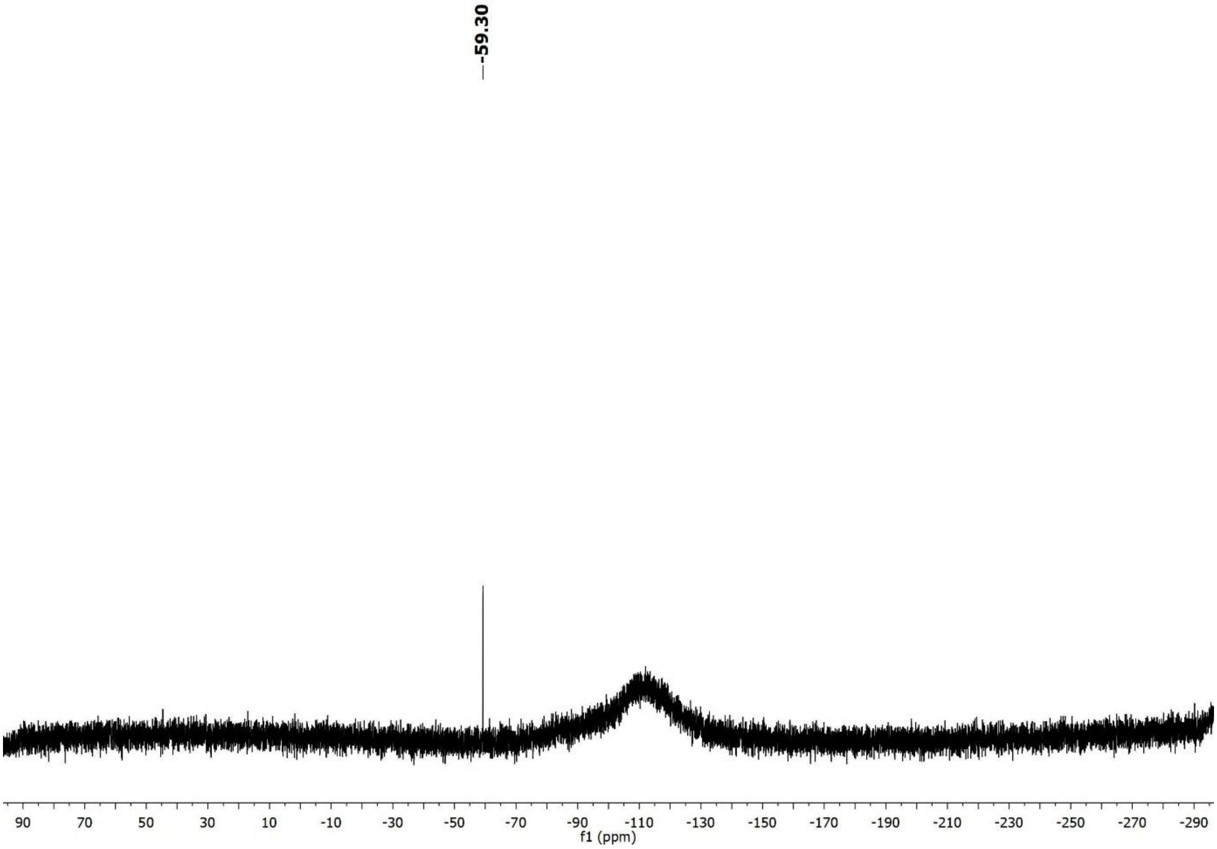

Figure S33. ^{29}Si NMR (80 MHz, Chloroform-d) of butyl((triisopropylgermyl)ethynyl)silane **(3bf)**

Phenyl((tributylgermyl)ethynyl)silane (3ca)

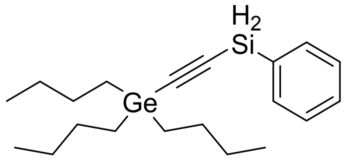

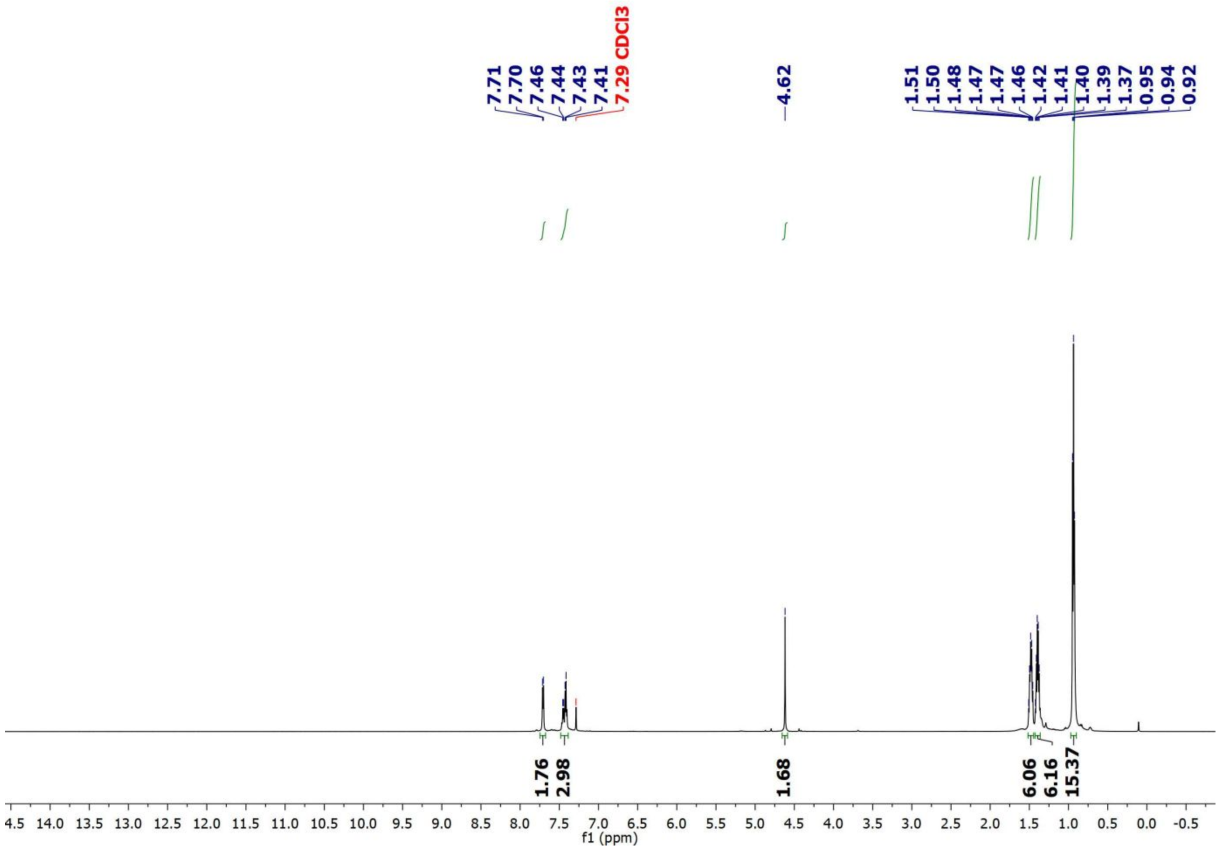

Figure S34. ^{1}H NMR (600 MHz, Chloroform-d) of phenyl((tributylgermyl)ethynyl)silane (**3ca**)

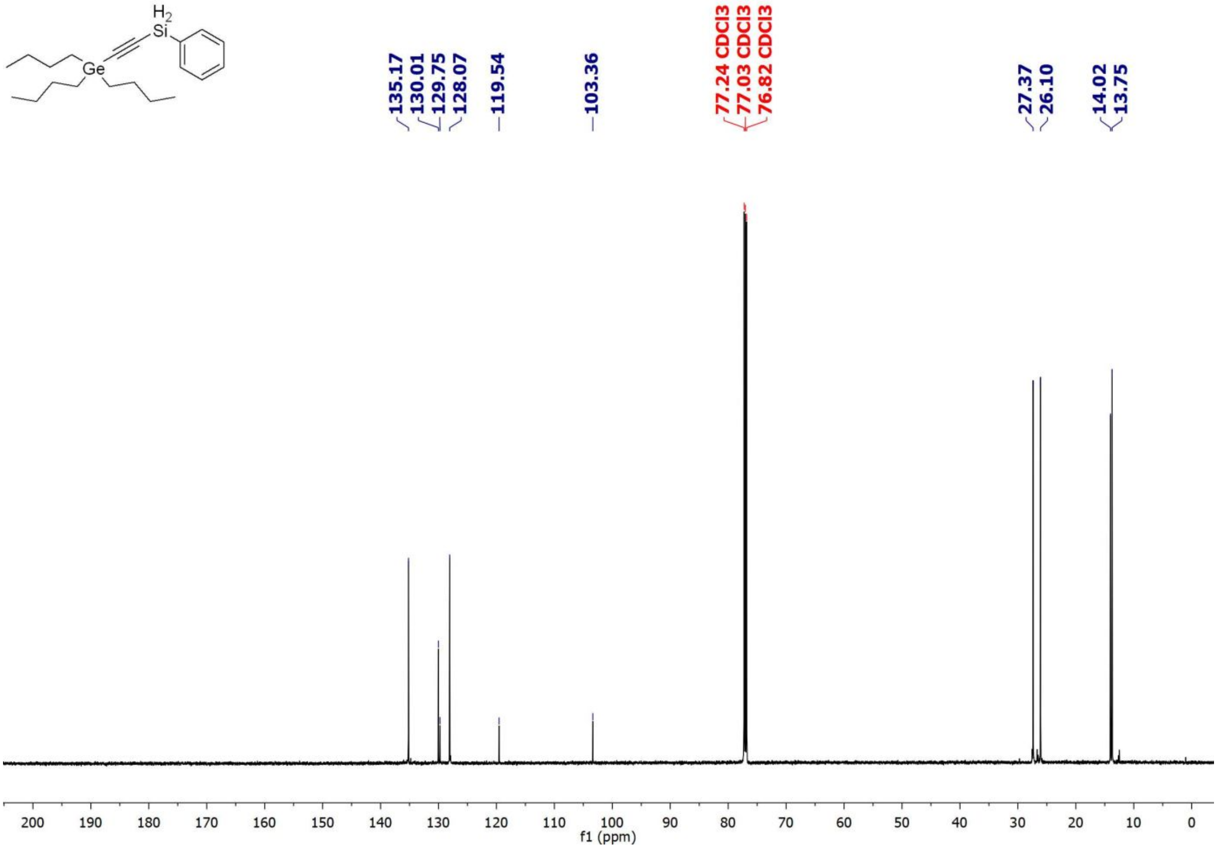

Figure S35. ^{13}C NMR (151 MHz, Chloroform-d) of phenyl((tributylgermyl)ethynyl)silane (**3ca**)

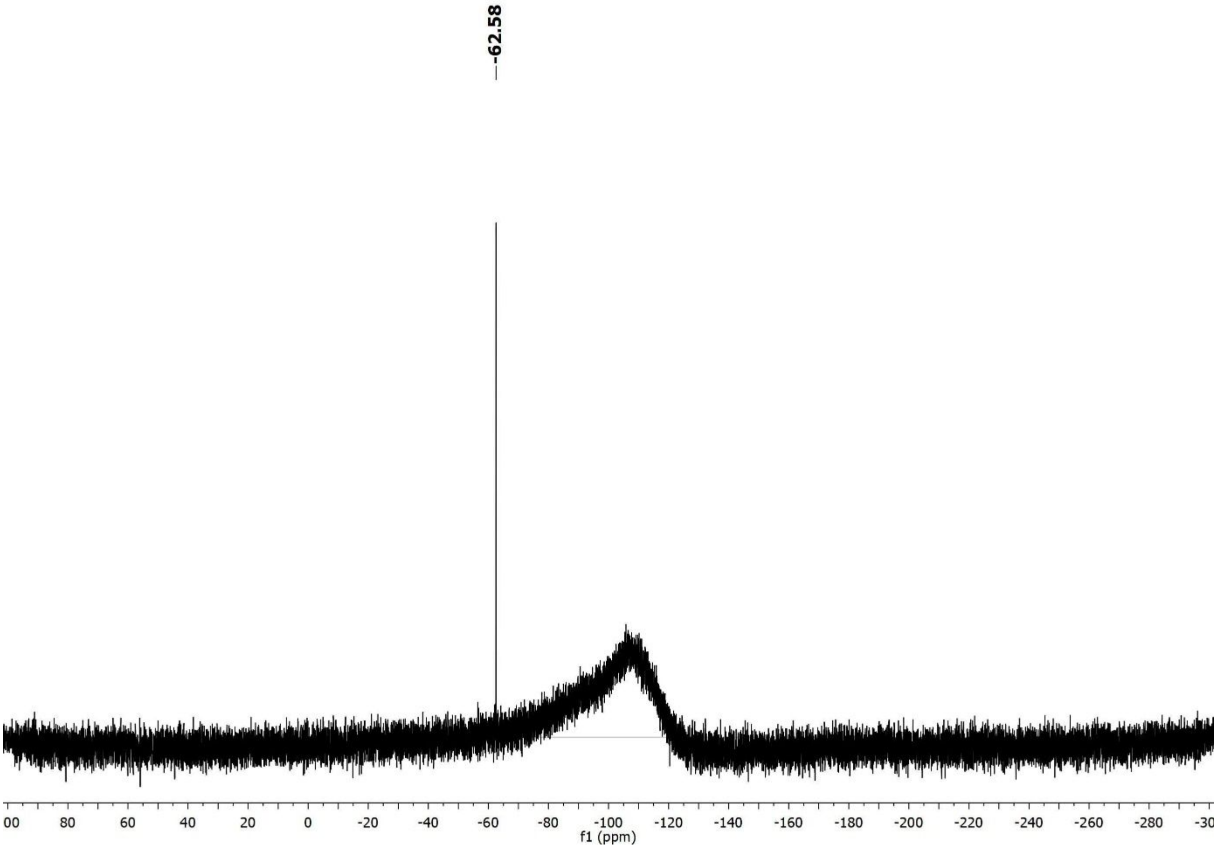

Figure S36. ^{29}Si NMR (119 MHz, Chloroform-d) of phenyl((tributylgermyl)ethynyl)silane (**3ca**)

Hexyl((tributylgermyl)ethynyl)silane (3cb)

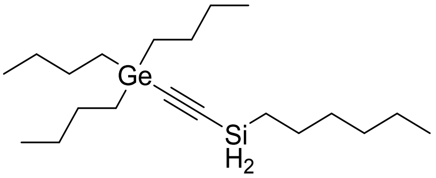

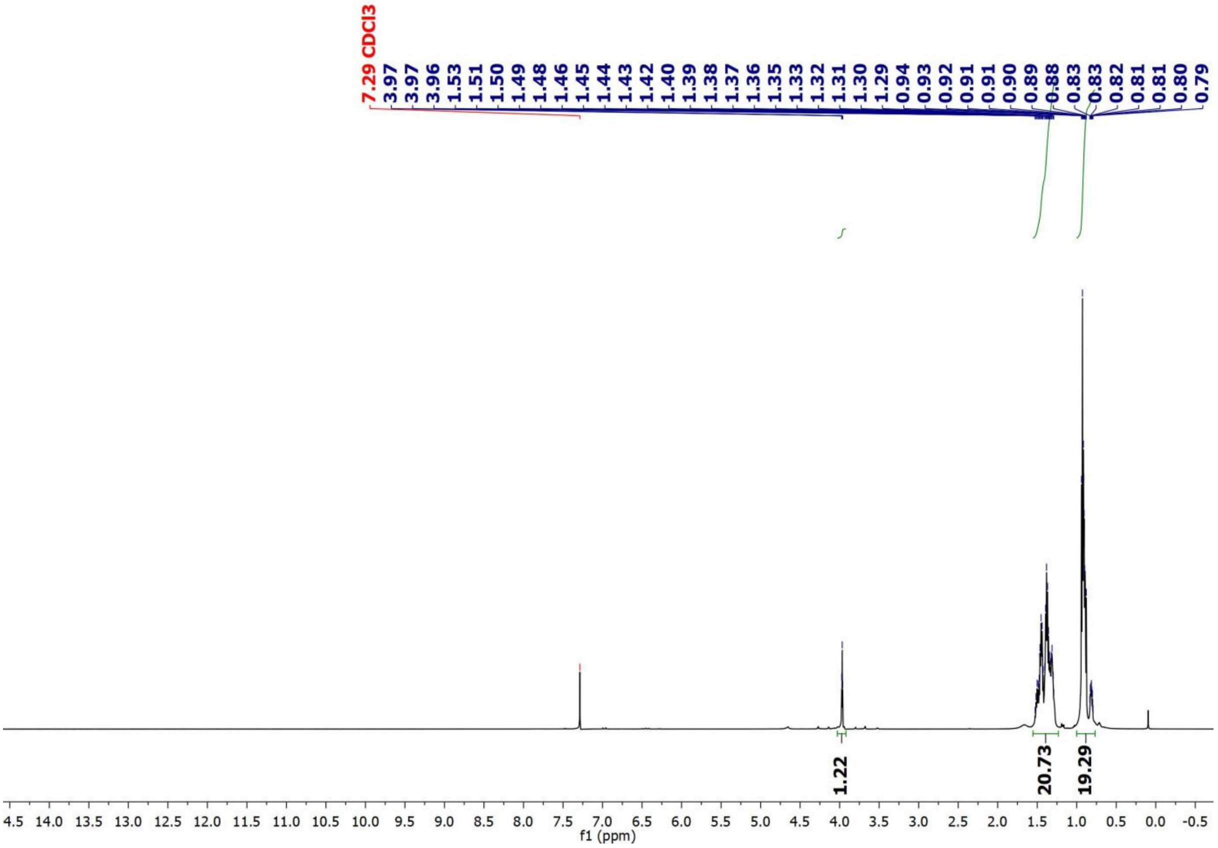

Figure S37. ^{1}H NMR (600 MHz, Chloroform-d) of hexyl((tributylgermyl)ethynyl)silane **(3cb)**

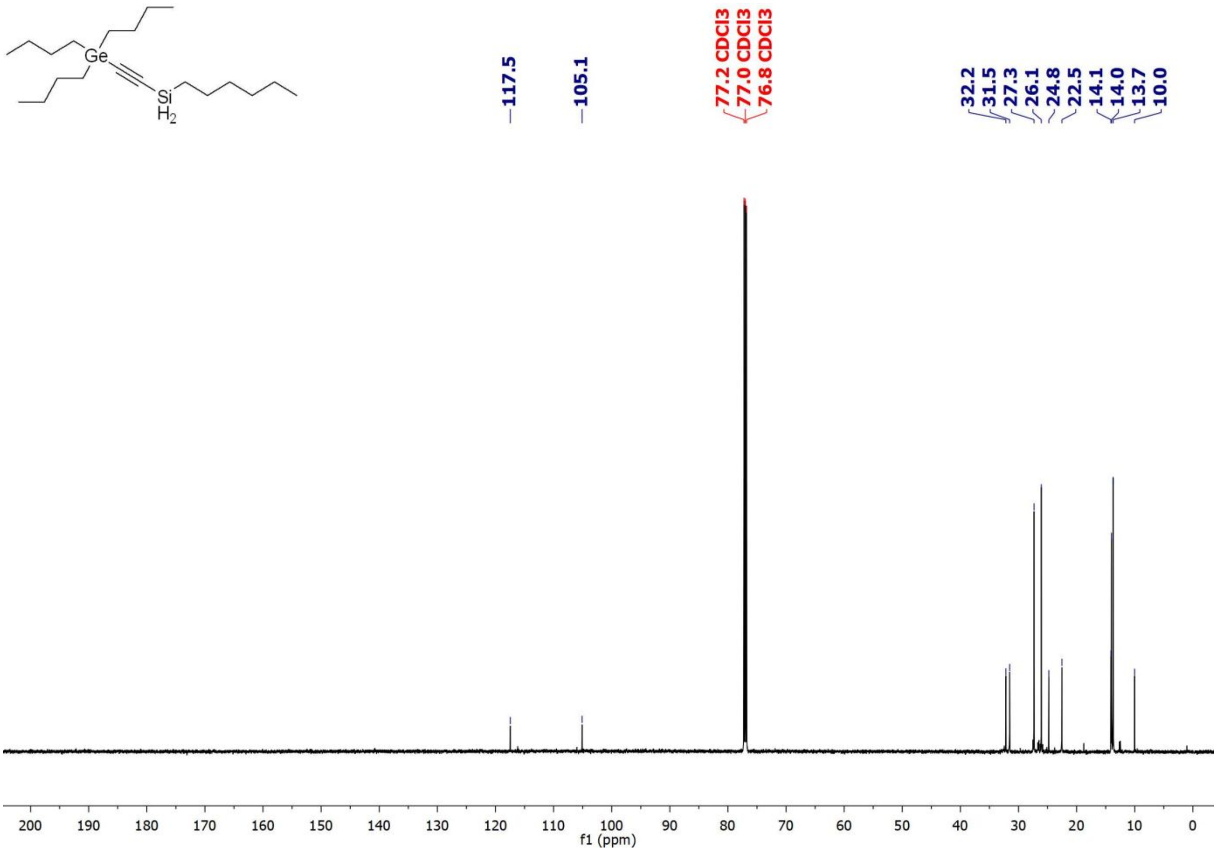

Figure S38. ^{13}C NMR (151 MHz, Chloroform-d) of hexyl((tributylgermyl)ethynyl)silane **(3cb)**

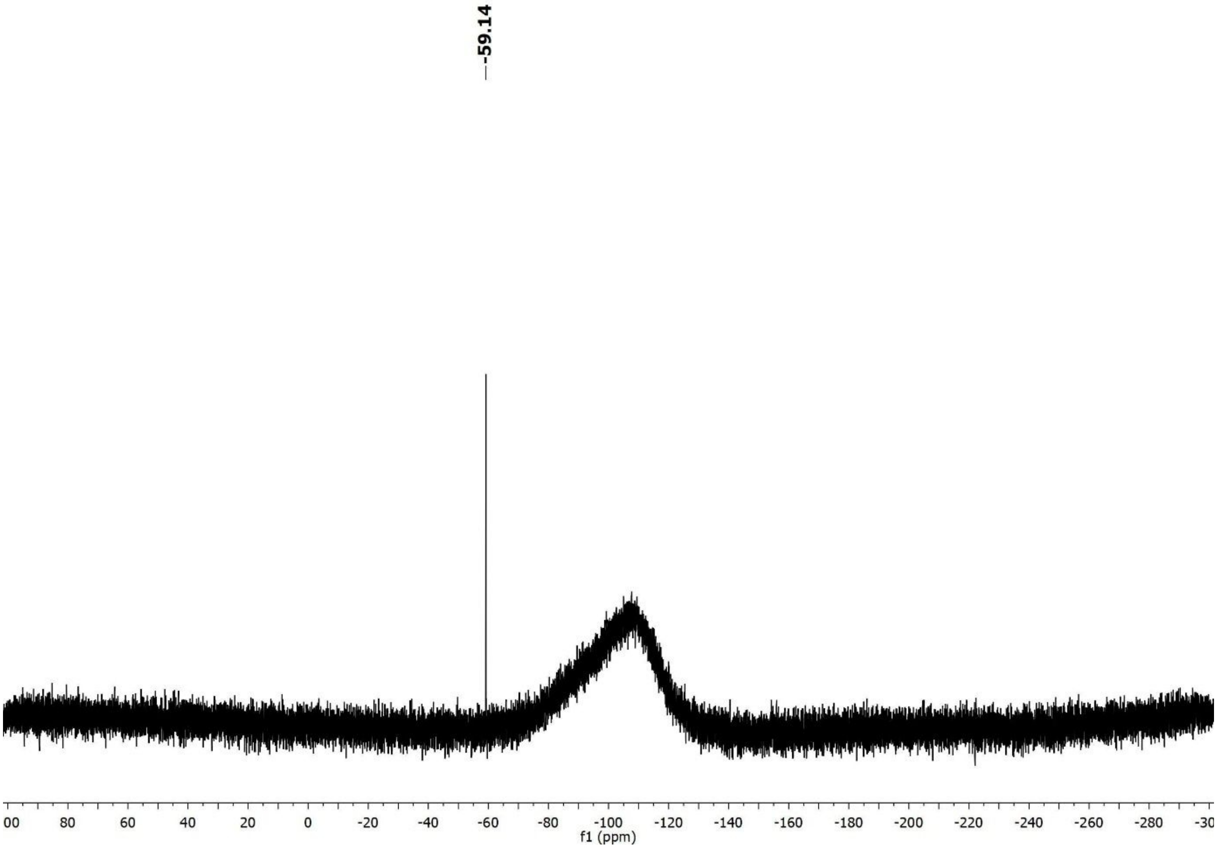

Figure S39. ^{29}Si NMR (119 MHz, Chloroform-d) of hexyl((tributylgermyl)ethynyl)silane **(3cb)**

p-Tolyl((tributylgermyl)ethynyl)silane (3cc)

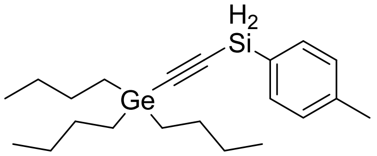

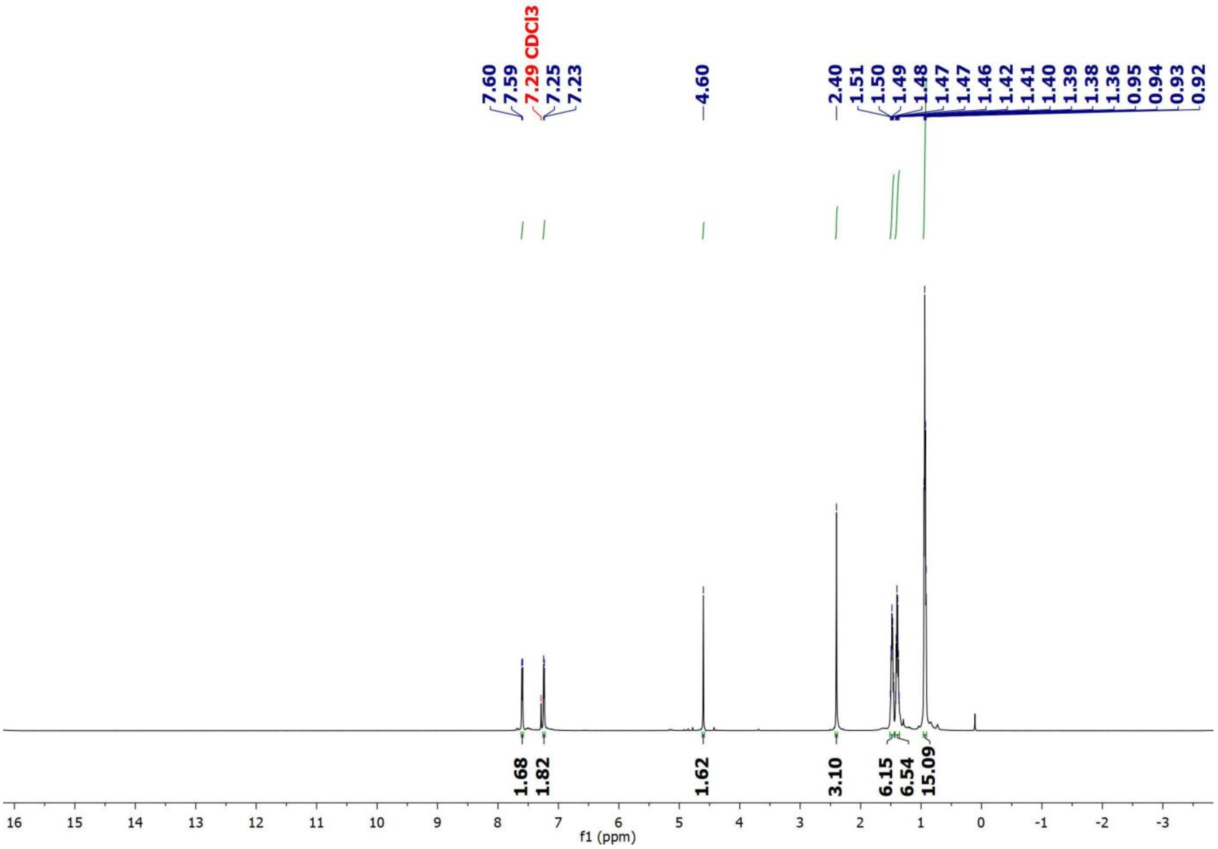

Figure S40. ^{1}H NMR (600 MHz, Chloroform-d) of *p*-tolyl((tributylgermyl)ethynyl)silane **(3cc)**

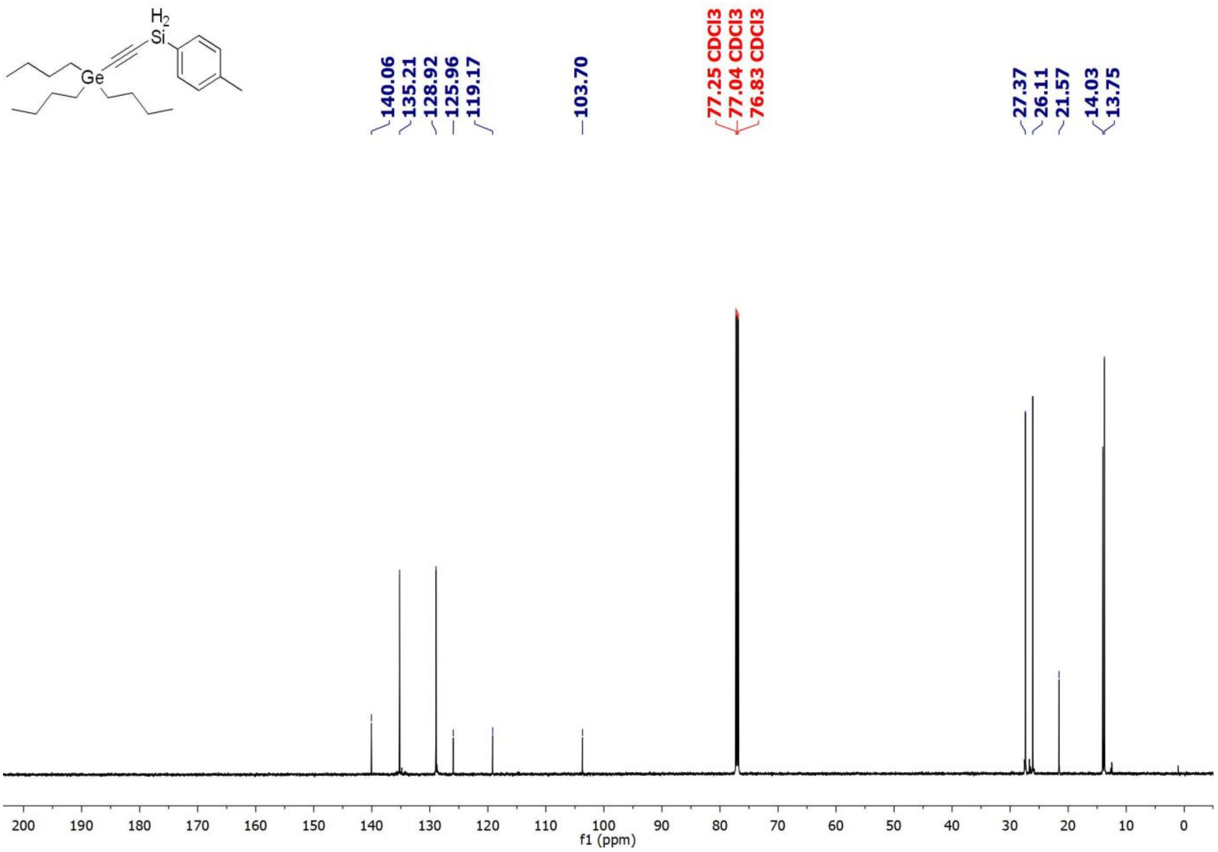

Figure S41. ^{13}C NMR (151 MHz, Chloroform-d) of *p*-tolyl((tributylgermyl)ethynyl)silane **(3cc)**

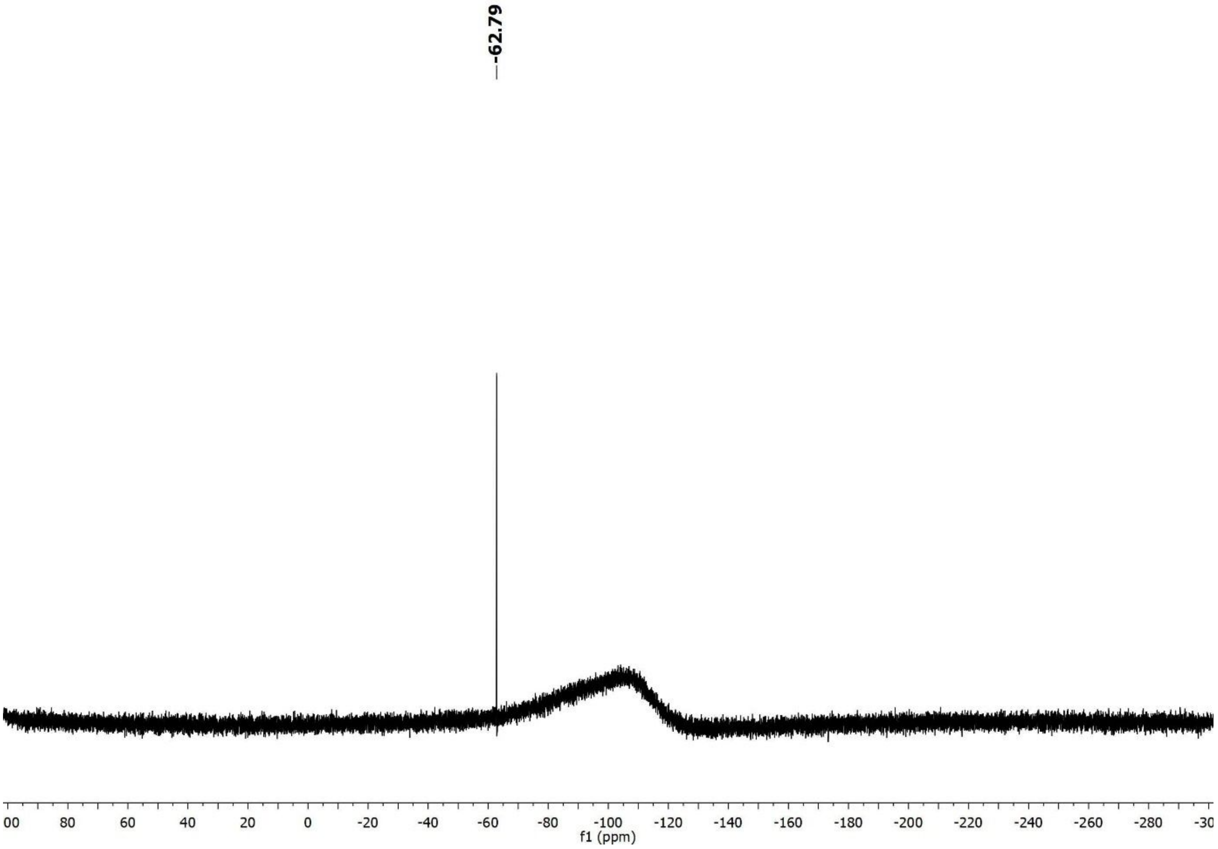

Figure S42. ^{29}Si NMR (119 MHz, Chloroform-d) of *p*-tolyl((tributylgermyl)ethynyl)silane **(3cc)**

Octyl((tributylgermyl)ethynyl)silane (3cd)

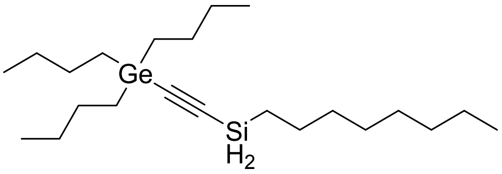

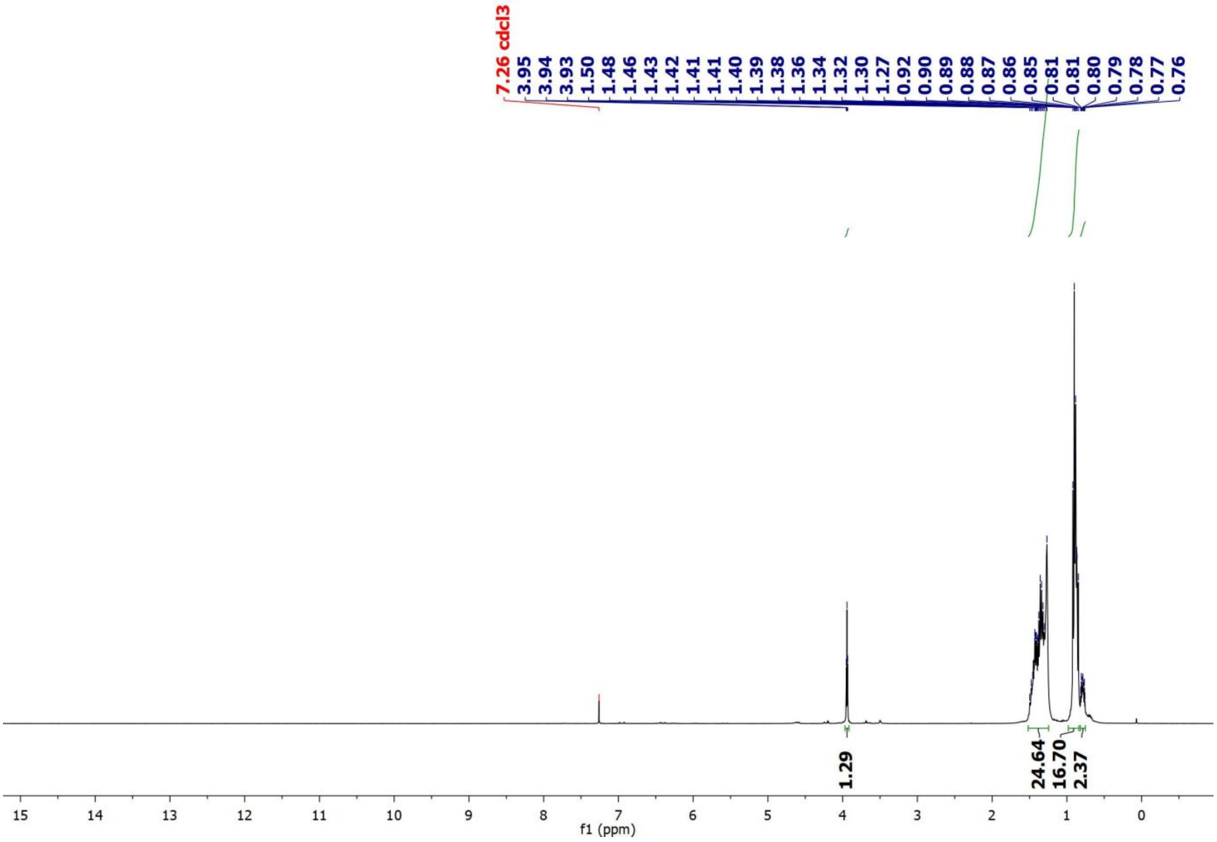

Figure S43. ^{1}H NMR (400 MHz, Chloroform-d) of octyl((tributylgermyl)ethynyl)silane **(3cd)**

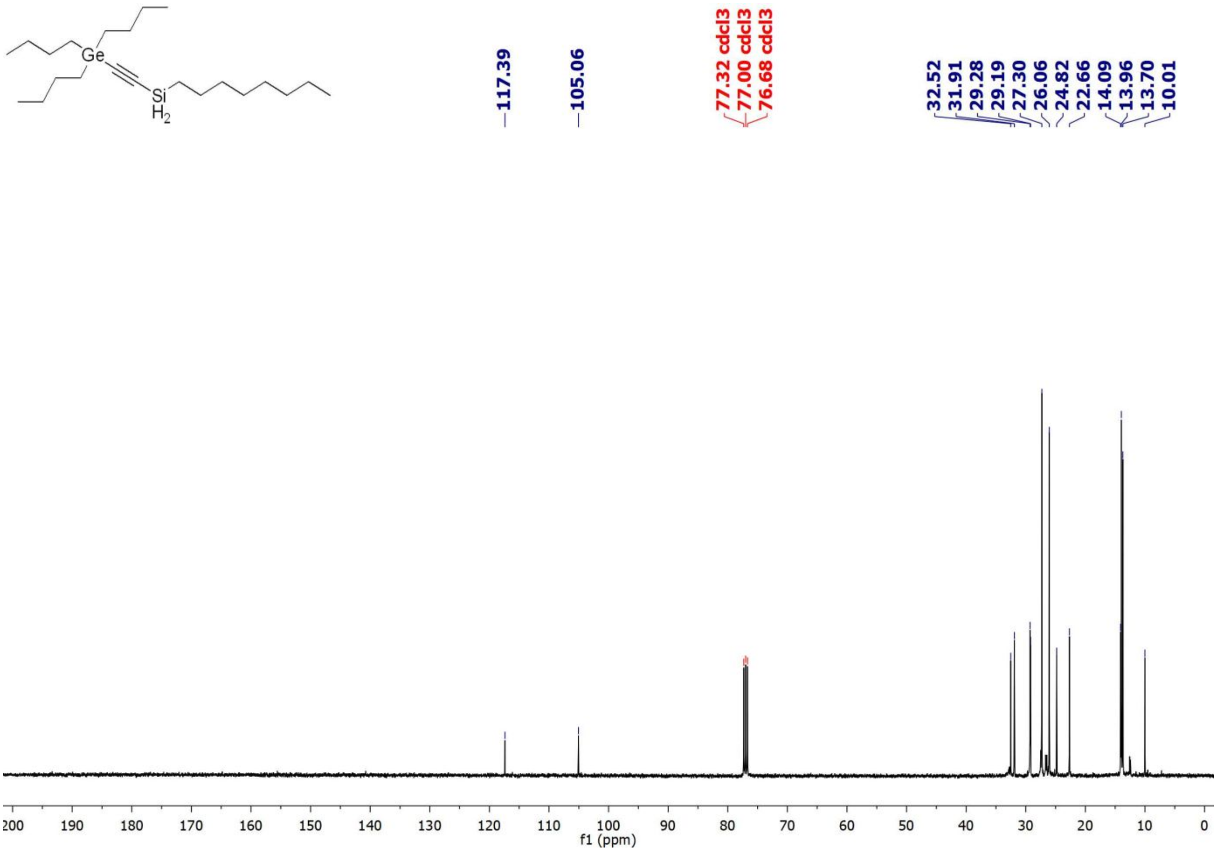

Figure S44. ^{13}C NMR (101 MHz, Chloroform-d) of octyl((tributylgermyl)ethynyl)silane **(3cd)**

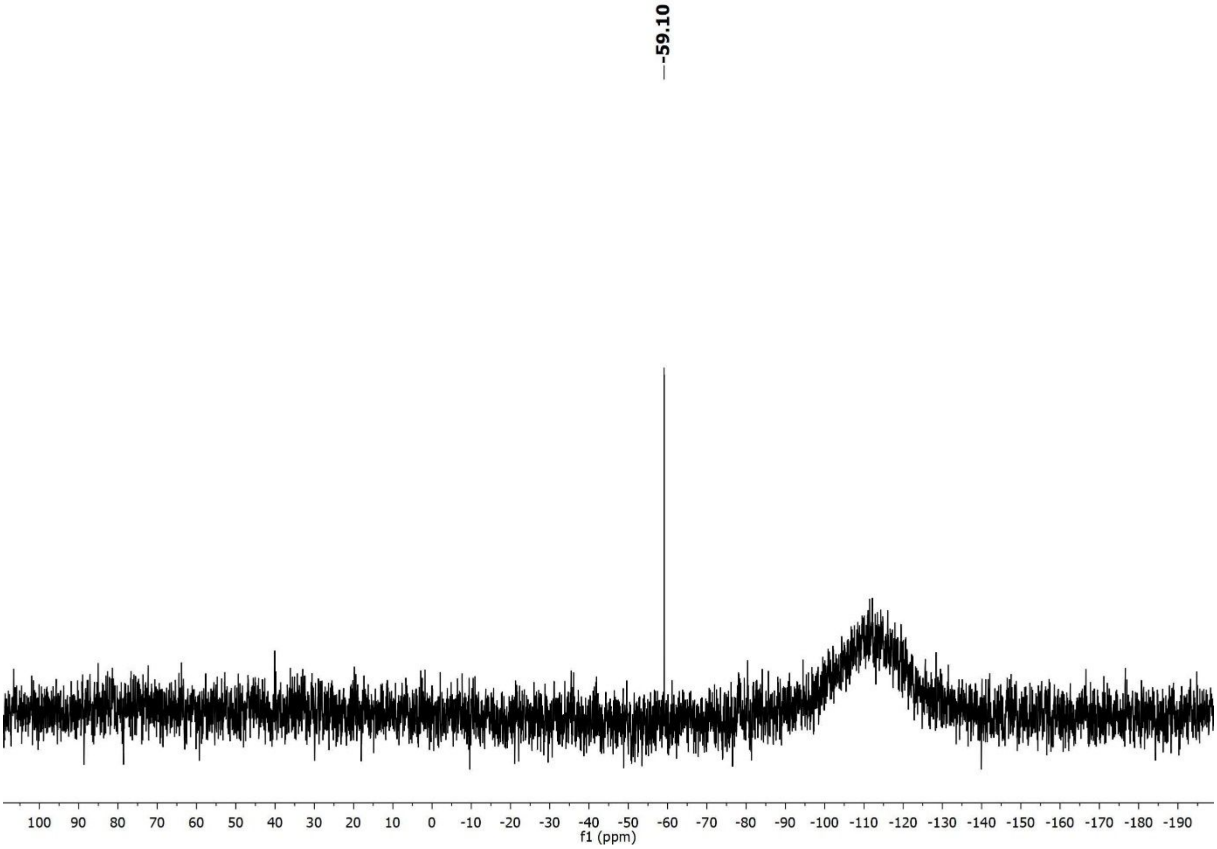

Figure S45. ^{29}Si NMR (79 MHz, Chloroform-d) of octyl((tributylgermyl)ethynyl)silane **(3cd)**

Cyclohexyl((tributylgermyl)ethynyl)silane (3ce)

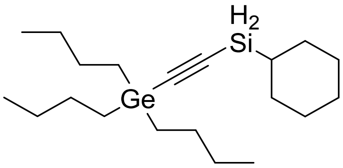

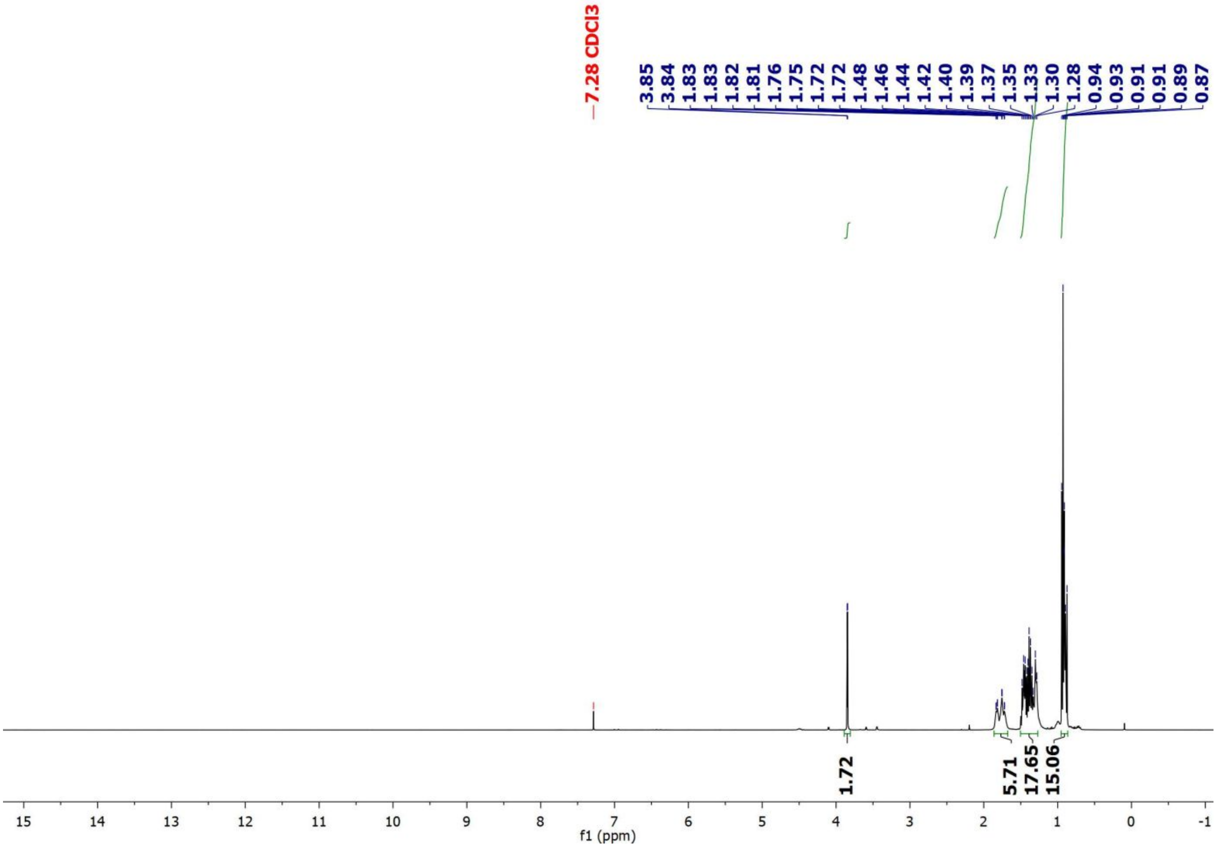

Figure S46. ^1H NMR (400 MHz, Chloroform-d) of cyclohexyl((tributylgermyl)ethynyl)silane **(3ce)**

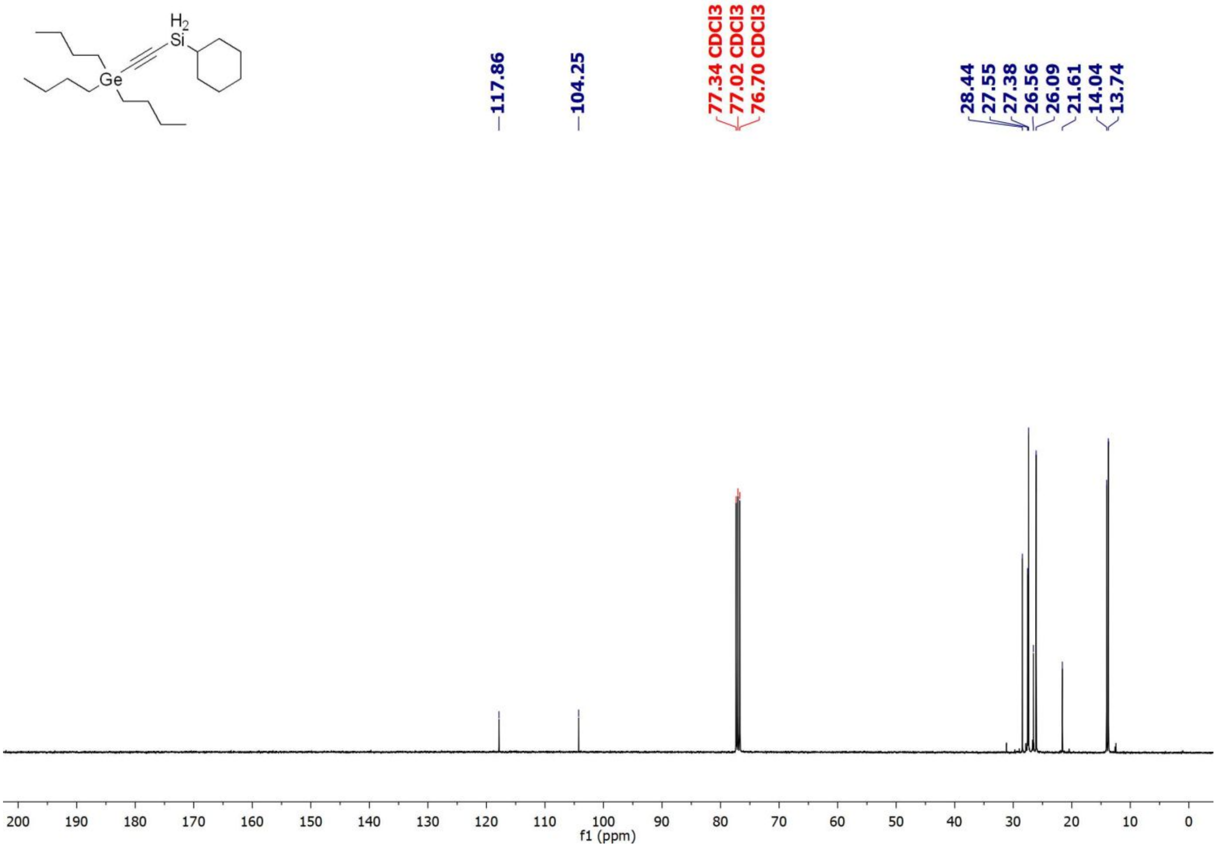

Figure S47. ^{13}C NMR (101 MHz, Chloroform-d) of cyclohexyl((tributylgermyl)ethynyl)silane **(3ce)**

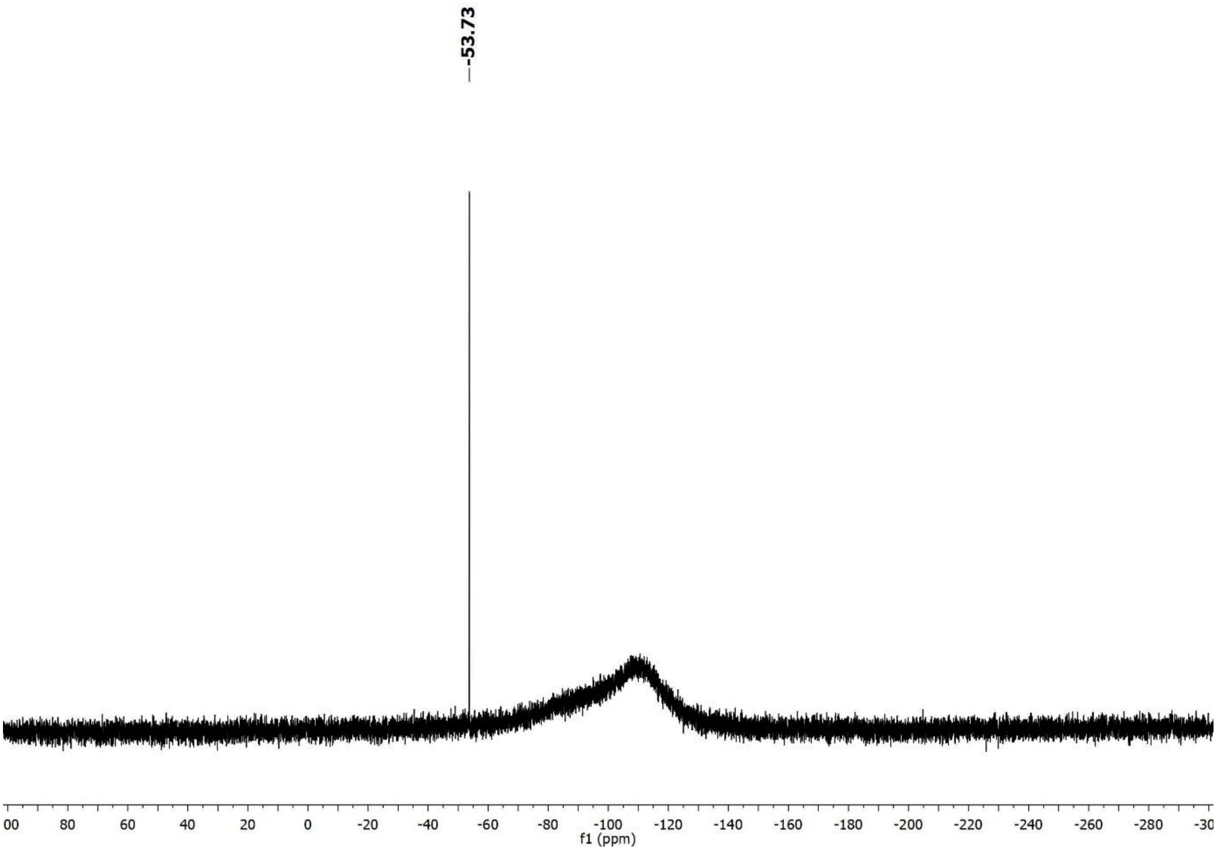

Figure S48. ^{29}Si NMR (79 MHz, Chloroform-d) of cyclohexyl((tributylgermyl)ethynyl)silane **(3ce)**

Methyl(phenyl)((triisopropylgermyl)ethynyl)silane (4bg)

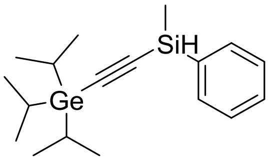

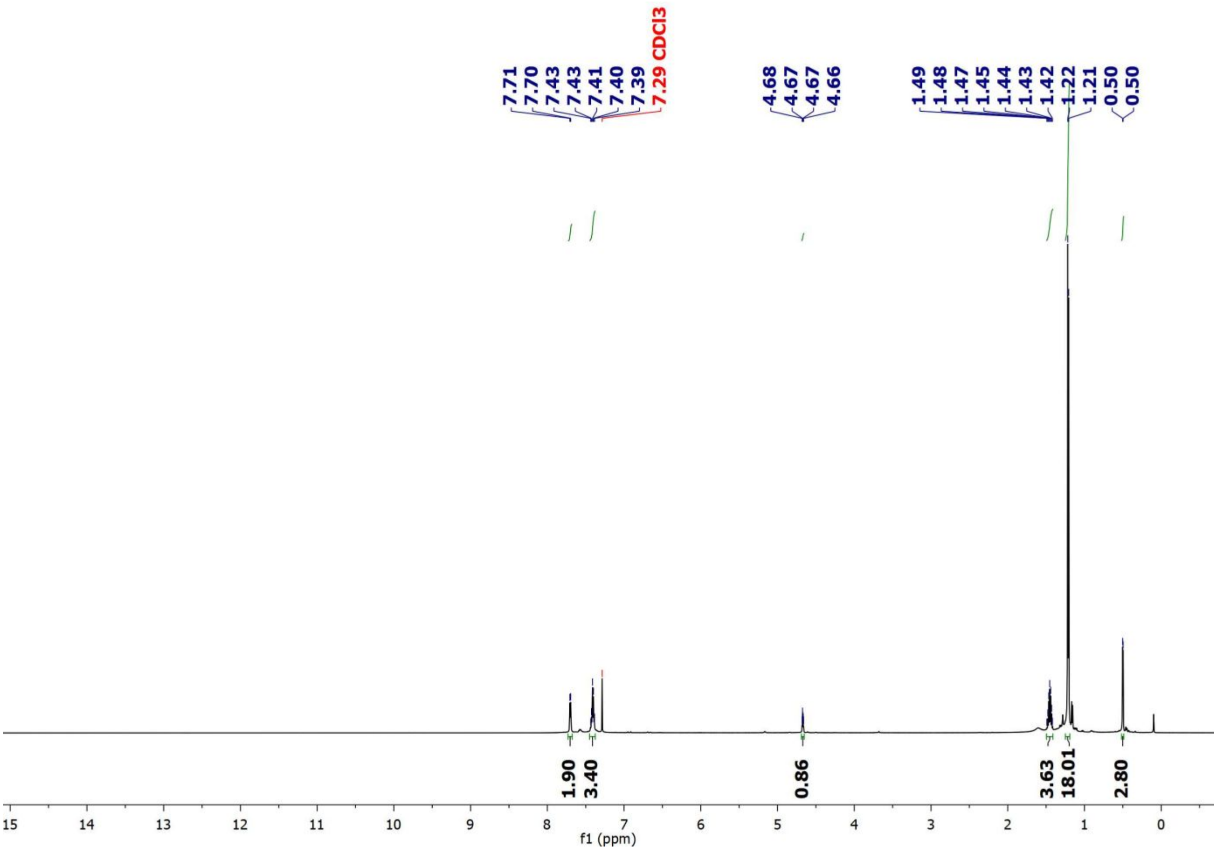

Figure S49. ^{1}H NMR (600 MHz, Chloroform-d) of methyl(phenyl)((triisopropylgermyl)ethynyl)silane **(4bg)**

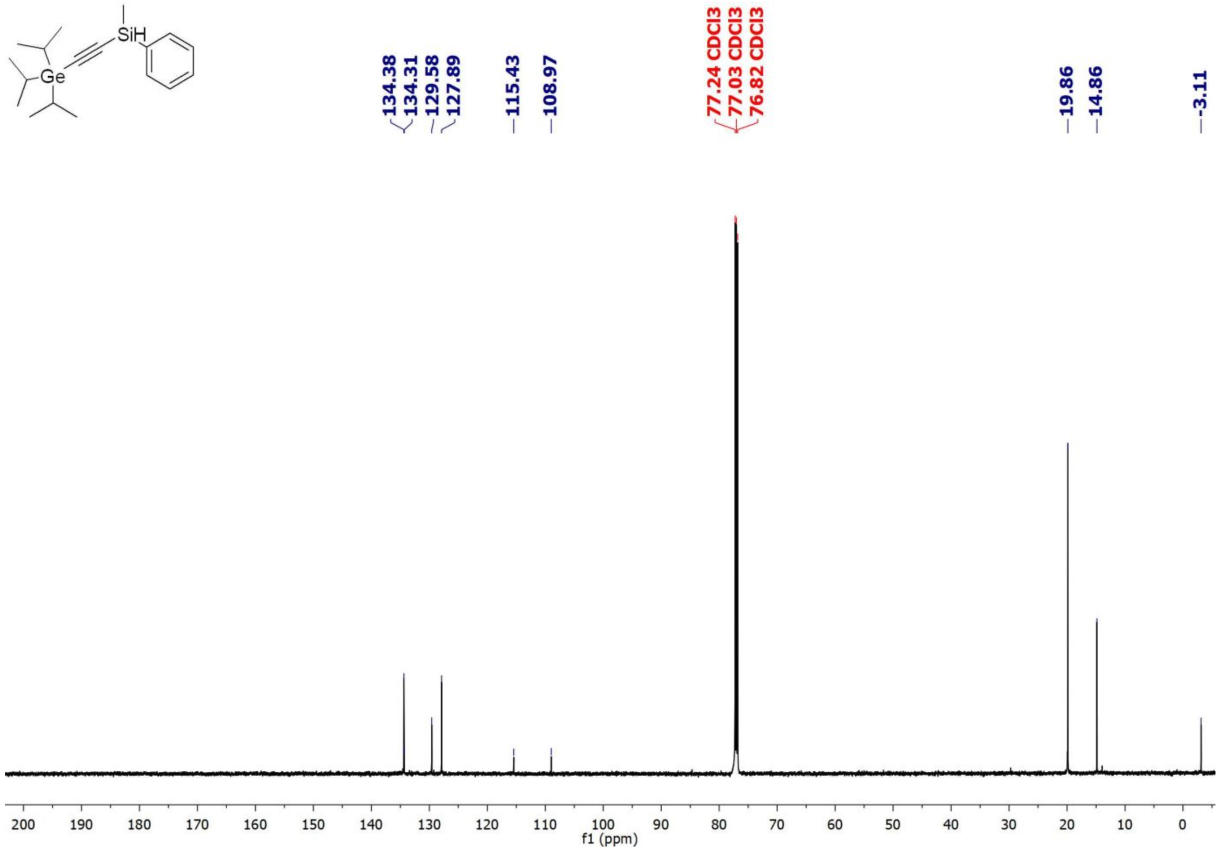

Figure S50. ^{13}C NMR (151 MHz, Chloroform-d) of methyl(phenyl)((triisopropylgermyl)ethynyl)silane **(4bg)**

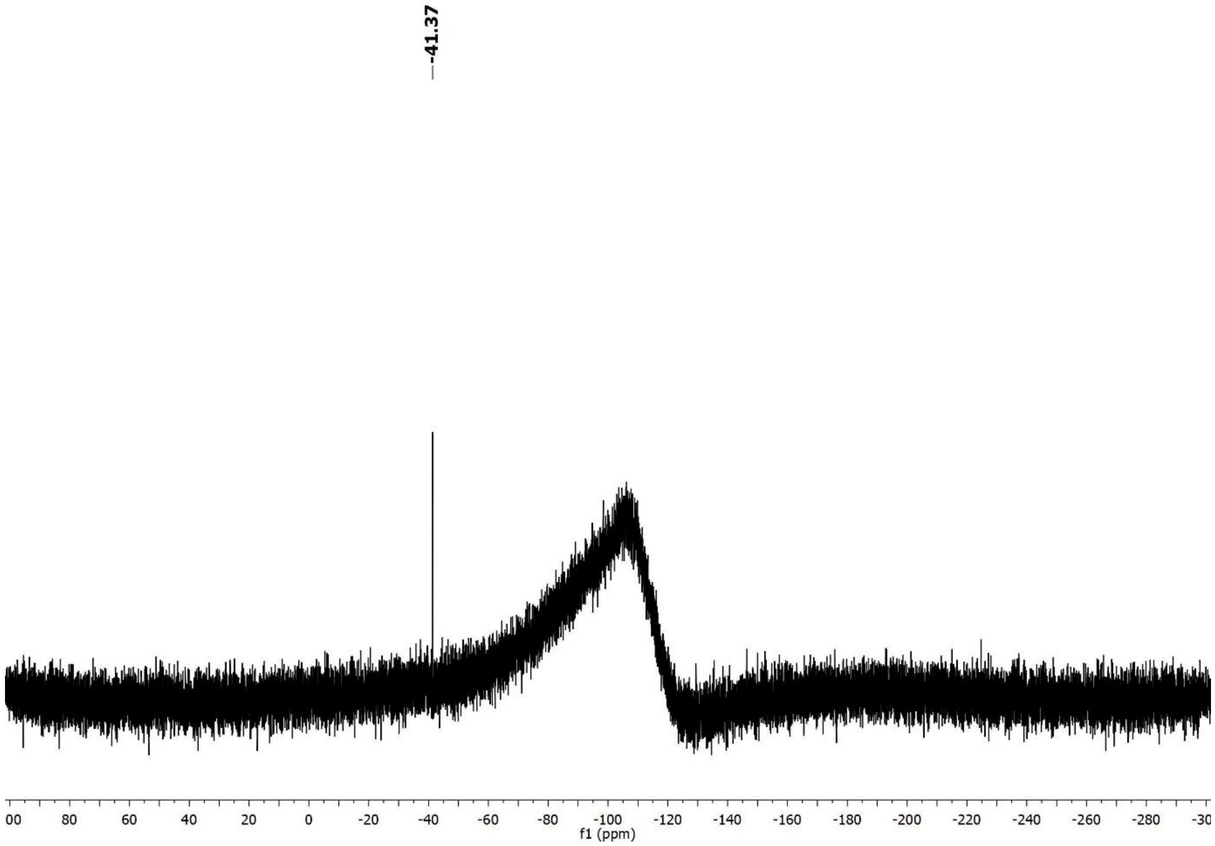

Figure S51. ^{29}Si NMR (119 MHz, Chloroform-d) of methyl(phenyl)((triisopropylgermyl)ethynyl)silane **(4bg)**

Diphenyl((triisopropylgermyl)ethynyl)silane (4bh)

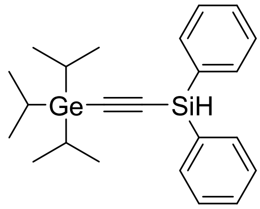

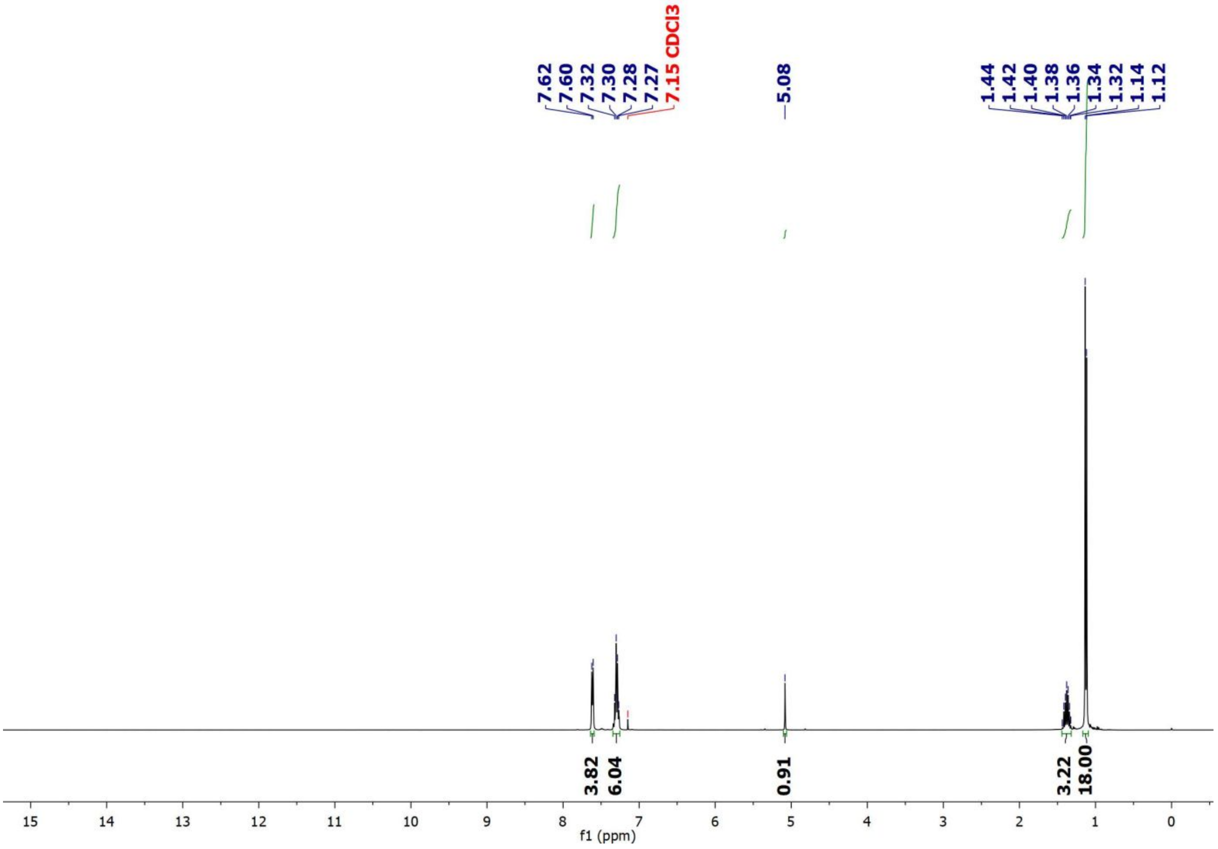

Figure S52. ^{1}H NMR (400 MHz, Chloroform-d) of diphenyl((triisopropylgermyl)ethynyl)silane **(4bh)**

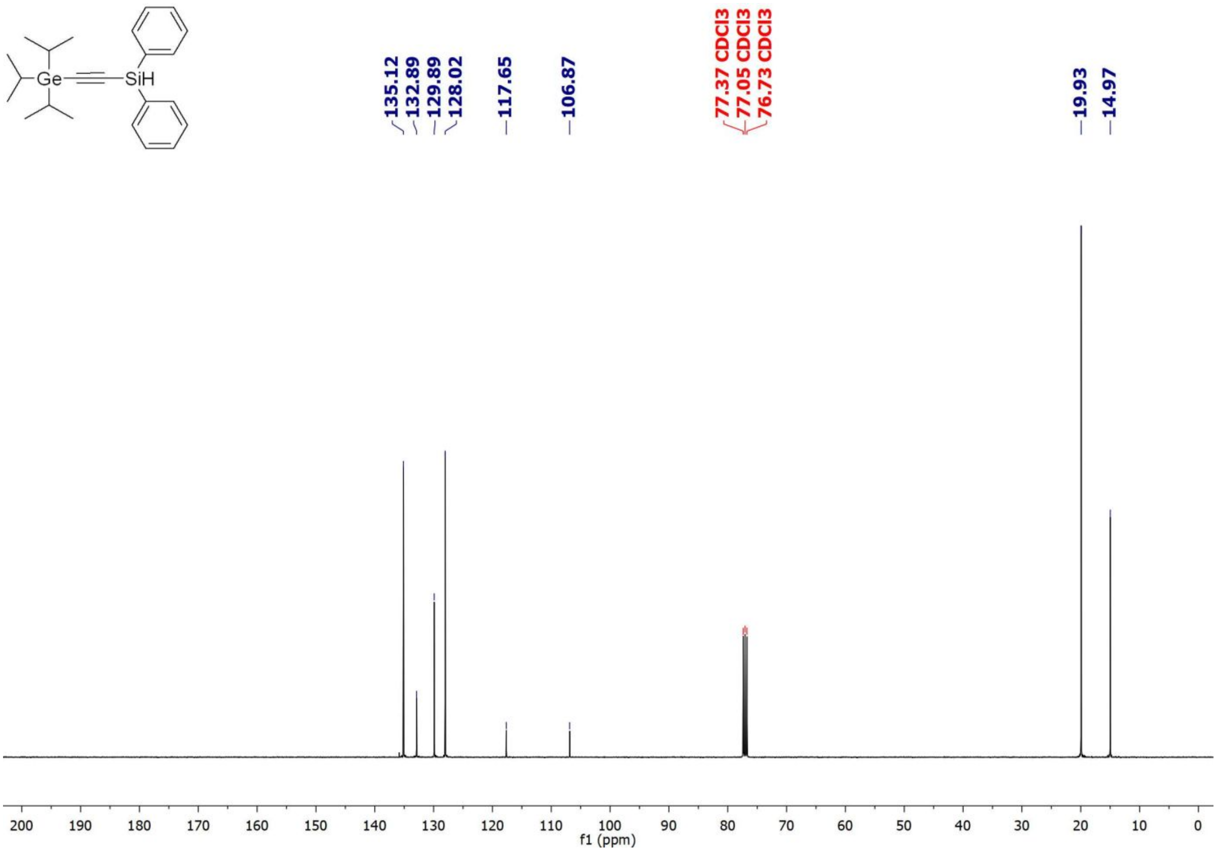

Figure S53. ^{13}C NMR (101 MHz, Chloroform-d) of diphenyl((triisopropylgermyl)ethynyl)silane **(4bh)**

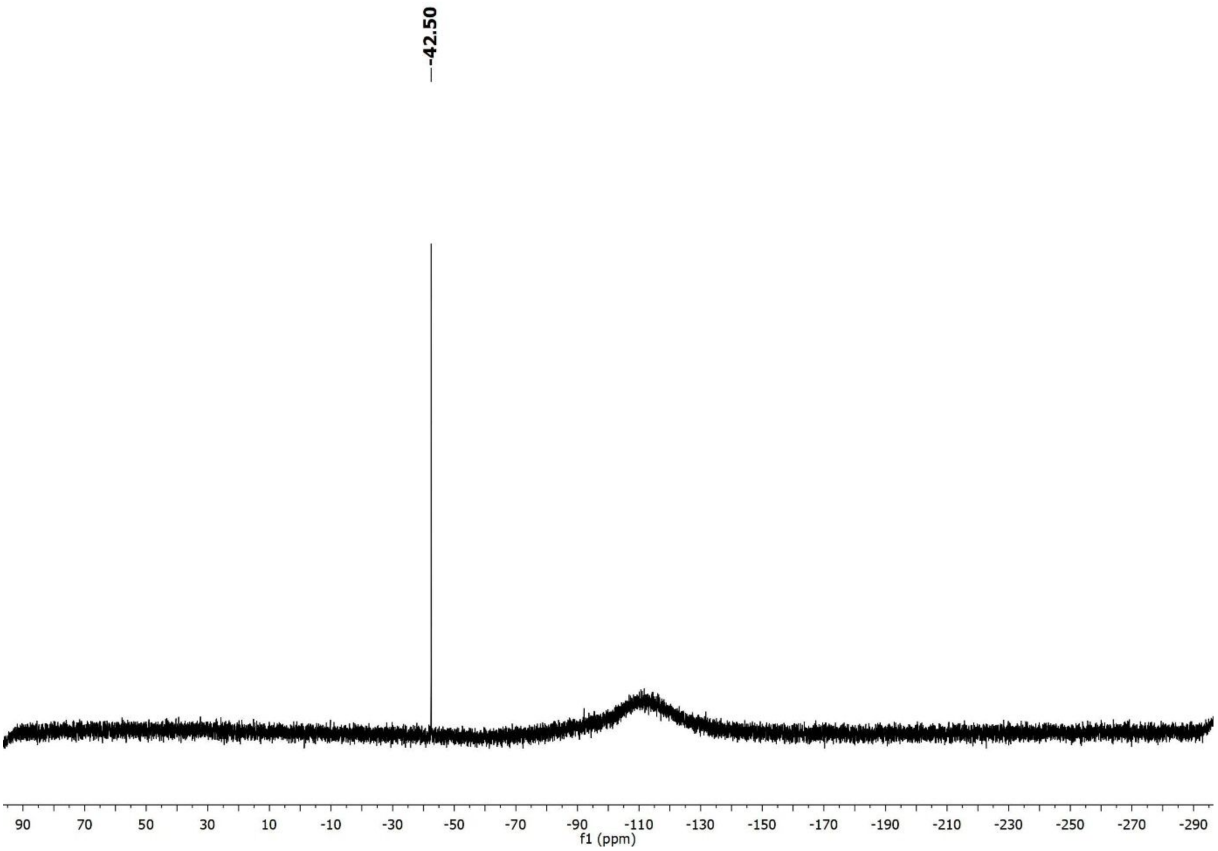

Figure S54. ^{29}Si NMR (79 MHz, Chloroform-d) of diphenyl((triisopropylgermyl)ethynyl)silane **(4bh)**

Diethyl((triisopropylgermyl)ethynyl)silane (4bi)

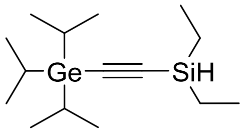

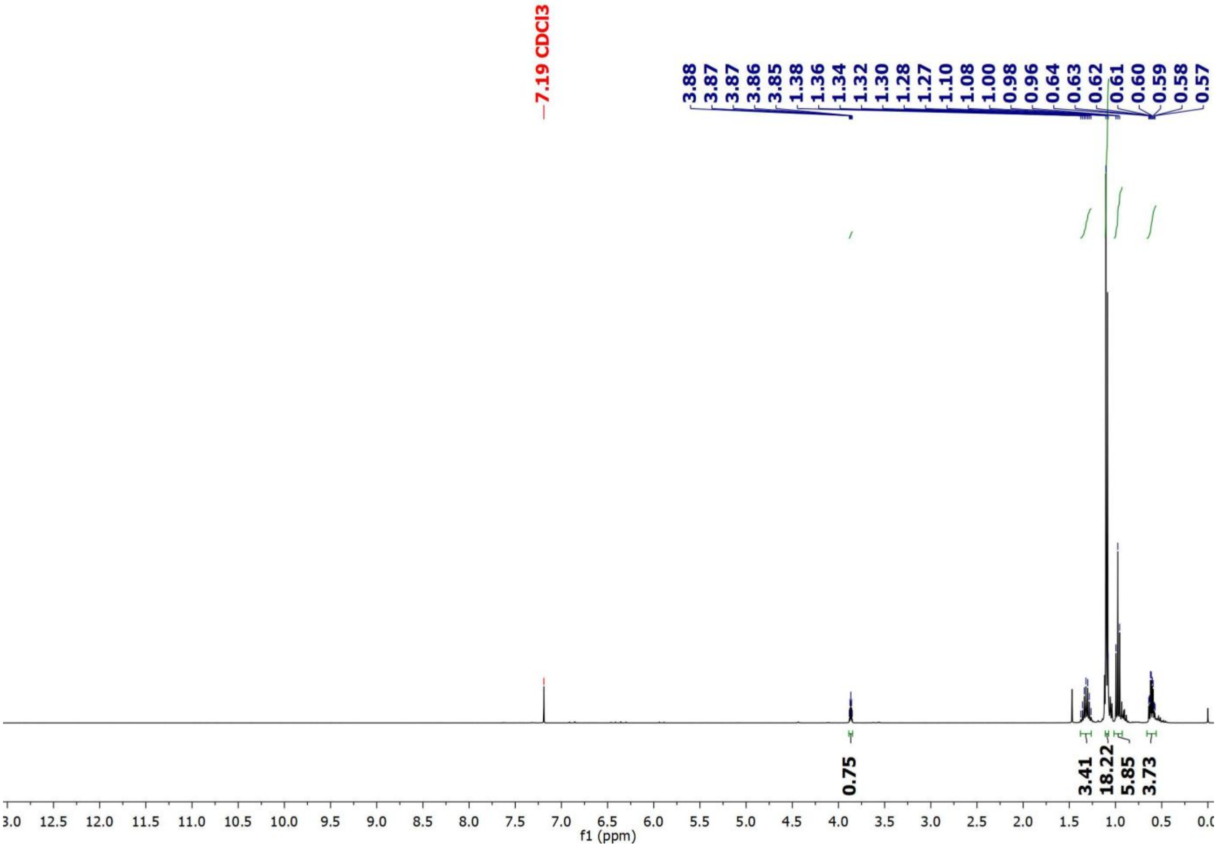

Figure S55. ^{1}H NMR (400 MHz, Chloroform-d) of diethyl((triisopropylgermyl)ethynyl)silane **(4bi)**

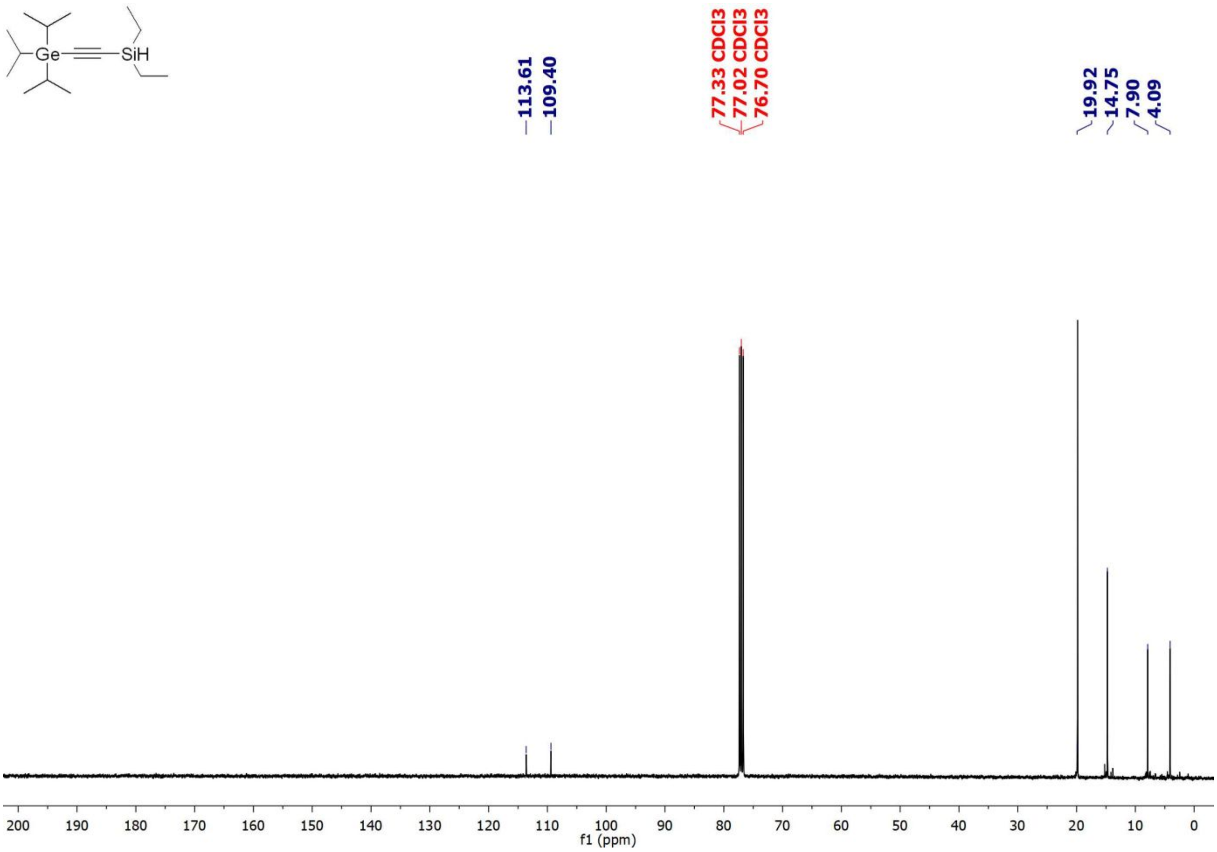

Figure S56. ^{13}C NMR (101 MHz, Chloroform-d) of diethyl((triisopropylgermyl)ethynyl)silane **(4bi)**

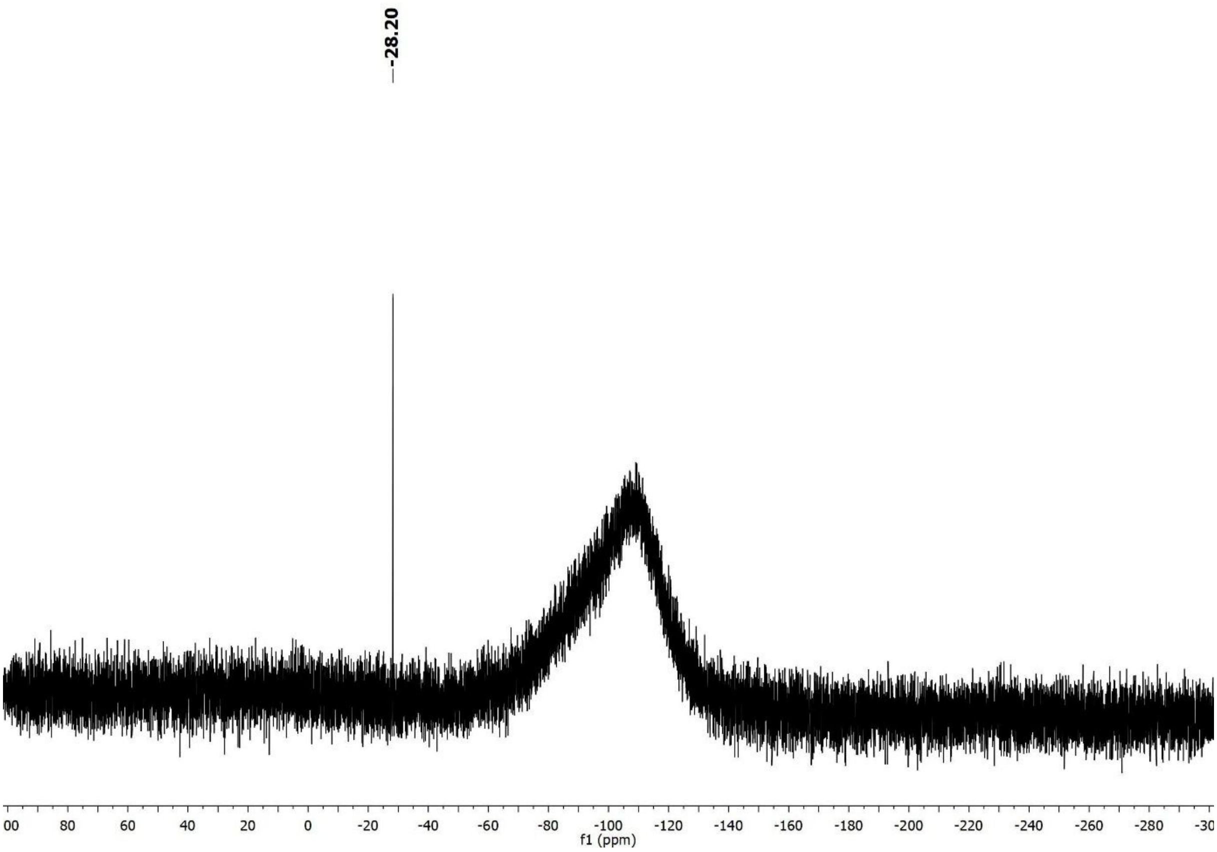

Figure S57. ^{29}Si NMR (79 MHz, Chloroform-d) of diethyl((triisopropylgermyl)ethynyl)silane **(4bi)**

Methyl(phenyl)(2-(triethylgermyl)vinyl)silane (5ag')

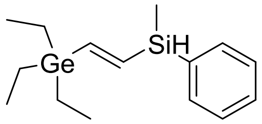

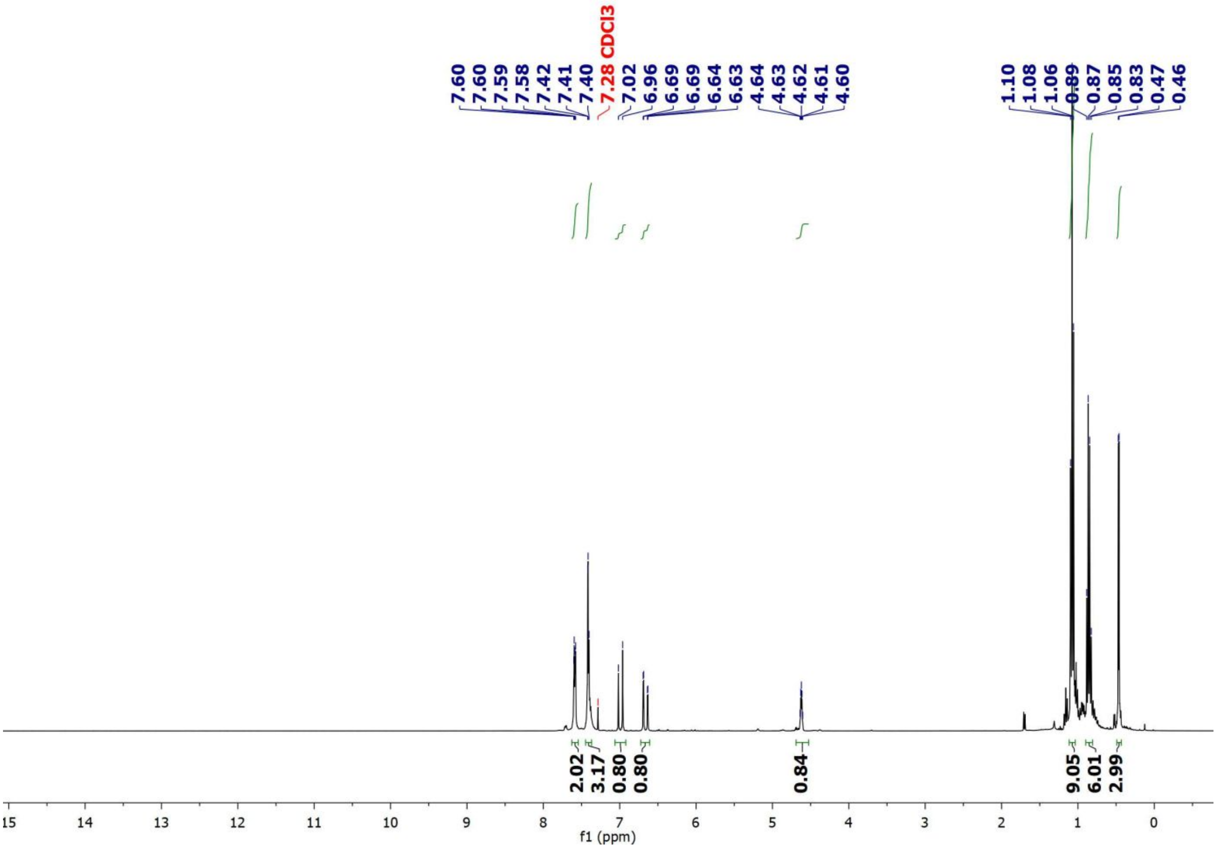

Figure S58. ^{1}H NMR (400 MHz, Chloroform-d) of methyl(phenyl)(2-(triethylgermyl)vinyl)silane **(5ag')**

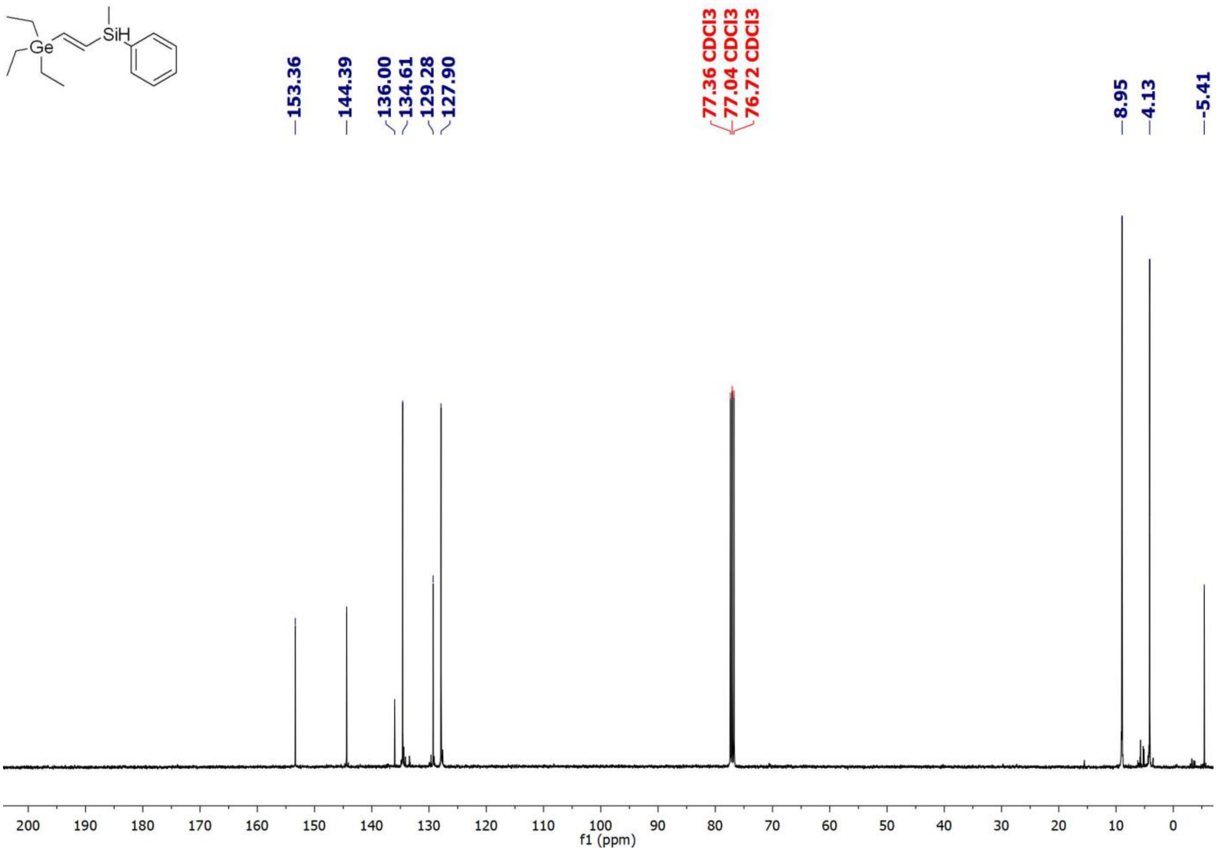

Figure S59. ^{13}C NMR (101 MHz, Chloroform-d) of methyl(phenyl)(2-(triethylgermyl)vinyl)silane **(5ag')**

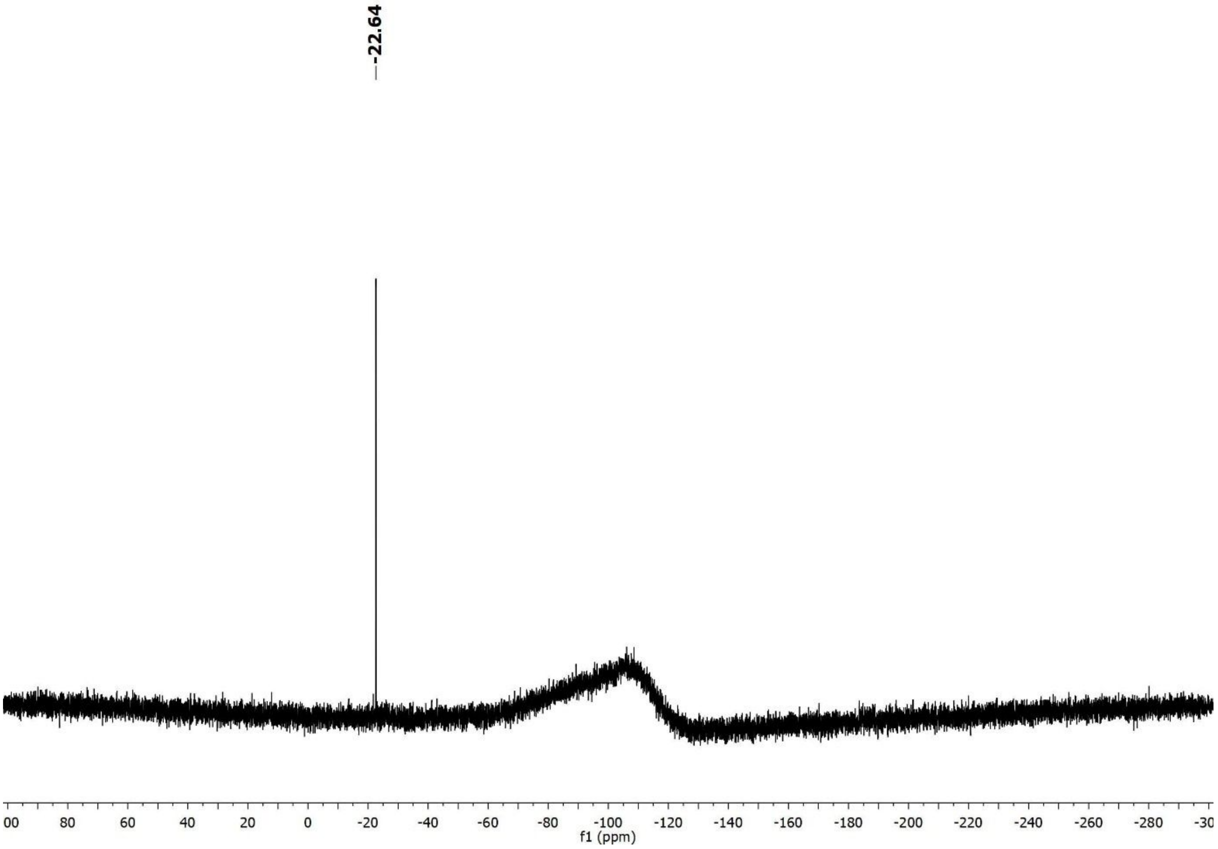

Figure S60. ^{29}Si NMR (79 MHz, Chloroform-d) of methyl(phenyl)(2-(triethylgermyl)vinyl)silane **(5ag')**

Diphenyl(2-(triethylgermyl)vinyl)silane (5ah')

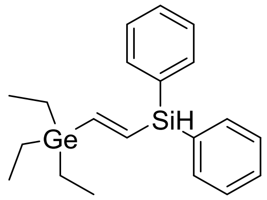

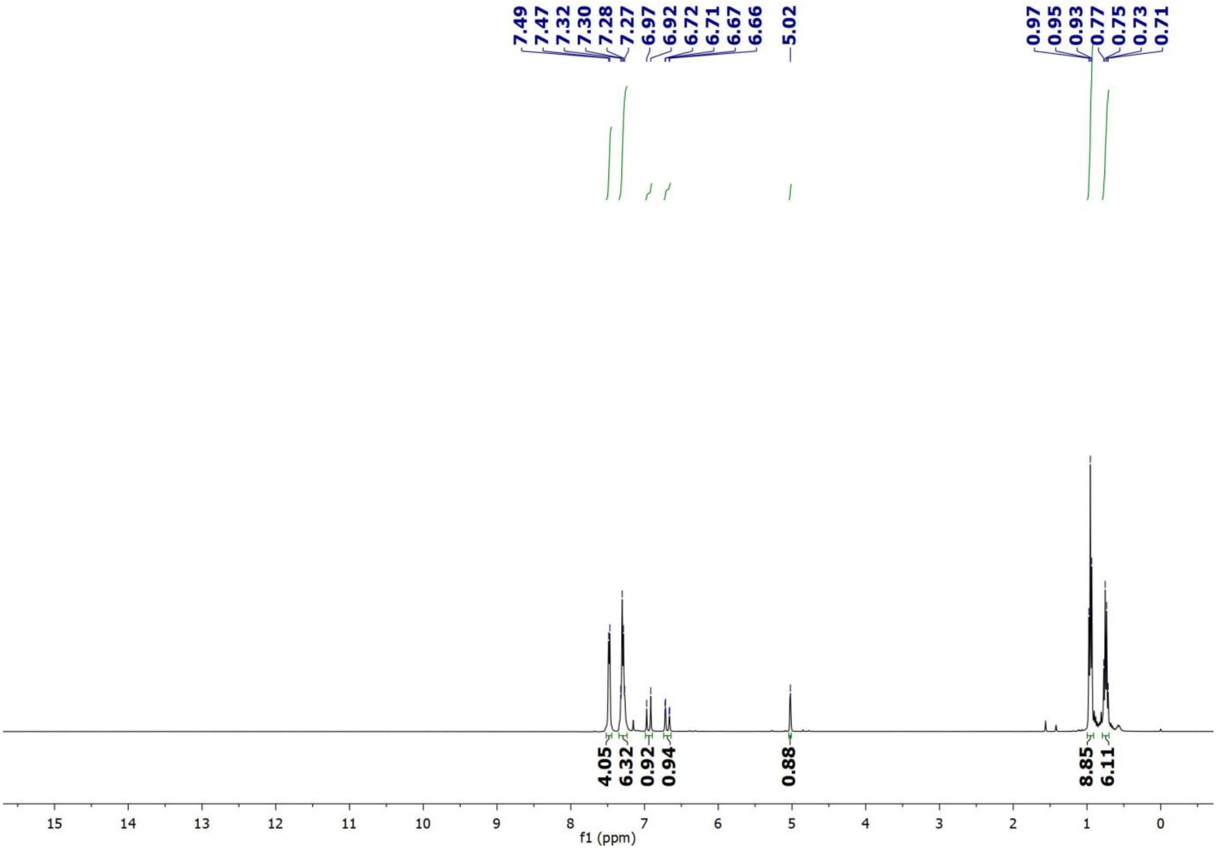

Figure S61. ^{1}H NMR (400 MHz, Chloroform-d) of diphenyl(2-(triethylgermyl)vinyl)silane **(5ah')**

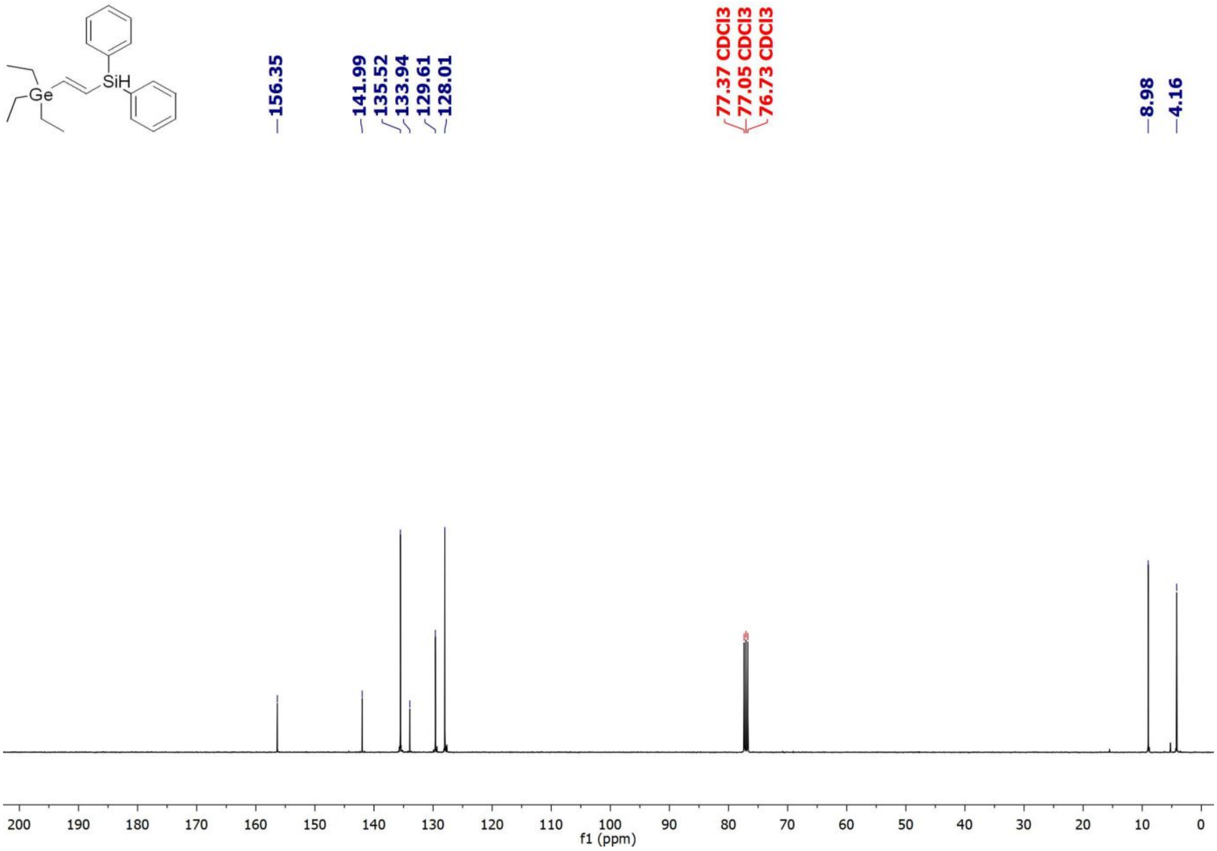

Figure S62. ^{13}C NMR (101 MHz, Chloroform-d) of diphenyl(2-(triethylgermyl)vinyl)silane **(5ah')**

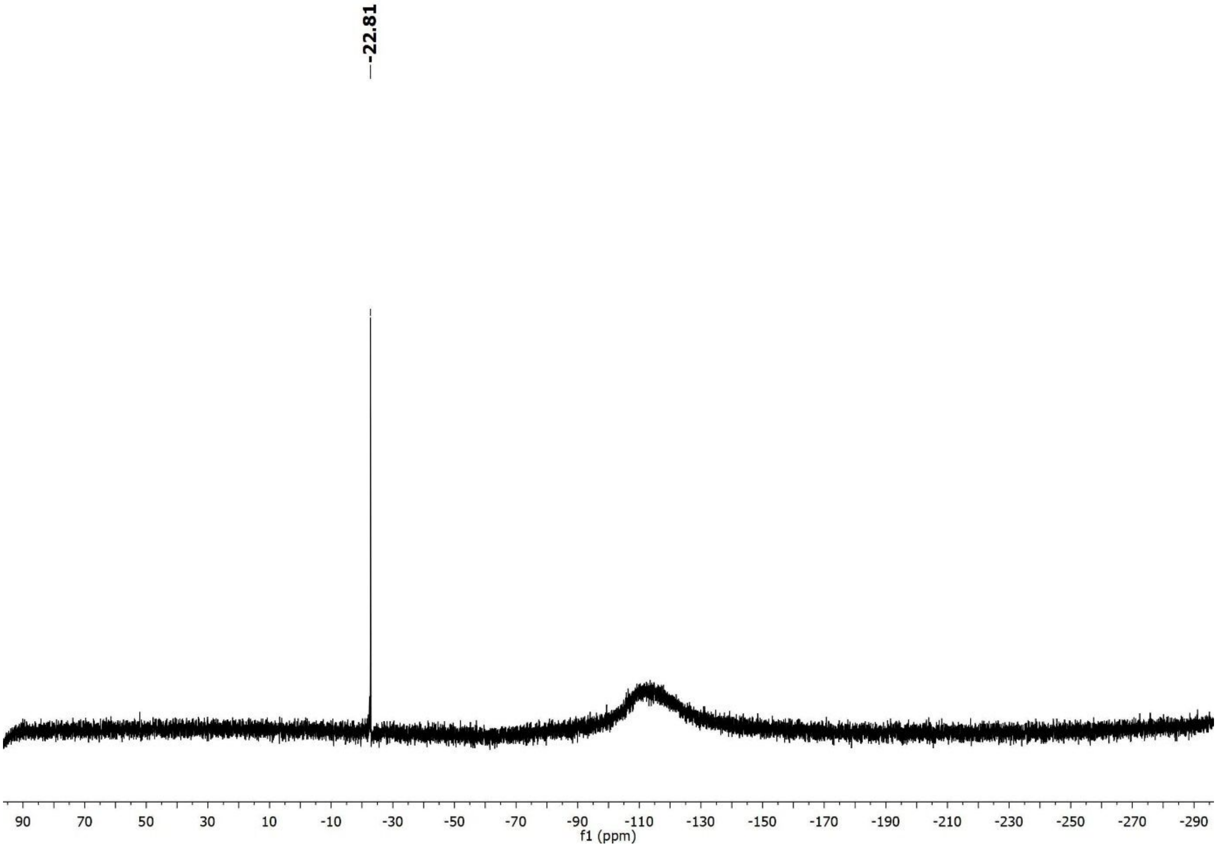

Figure S63. ^{29}Si NMR (79 MHz, Chloroform-d) of diphenyl(2-(triethylgermyl)vinyl)silane **(5ah')**

Diethyl(2-(triethylgermyl)vinyl)silane (5ai')

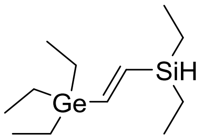

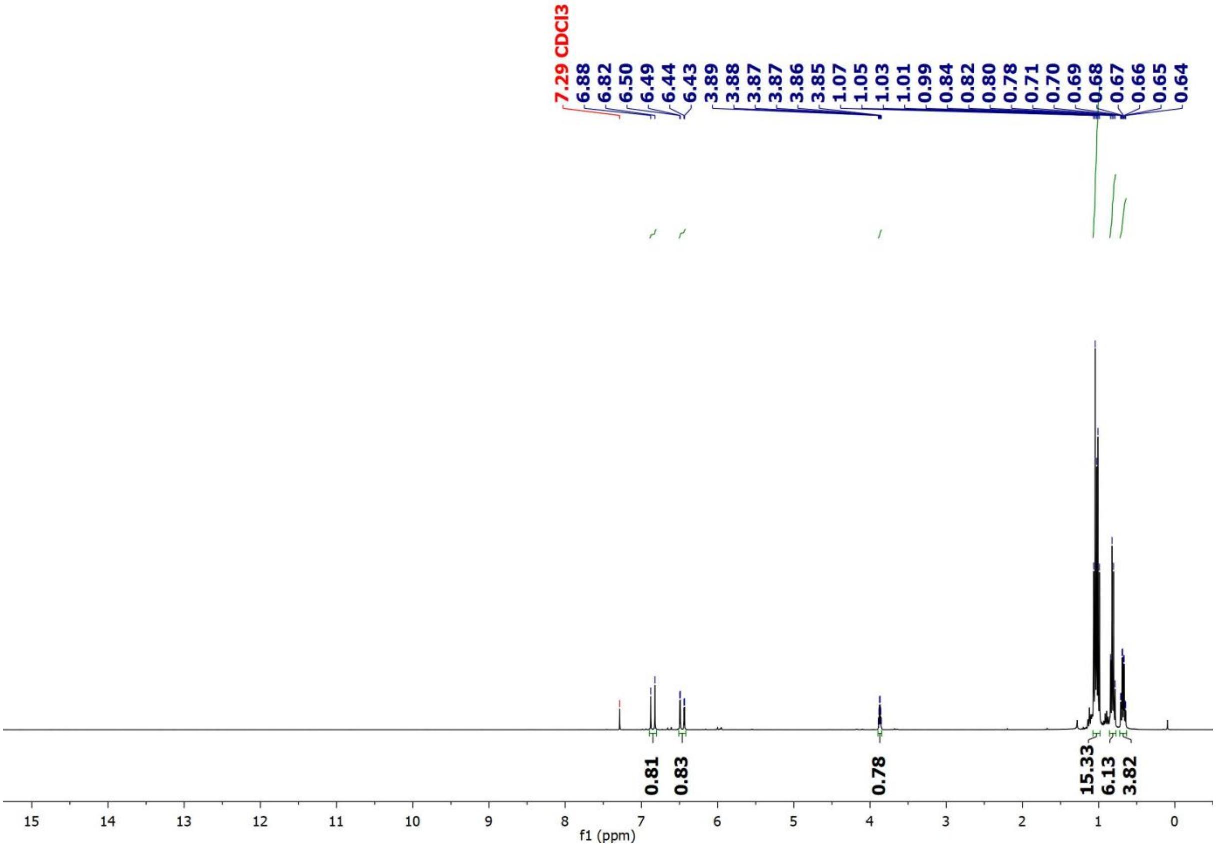

Figure S64. ^{1}H NMR (400 MHz, Chloroform-d) of diethyl(2-(triethylgermyl)vinyl)silane **(5ai')**

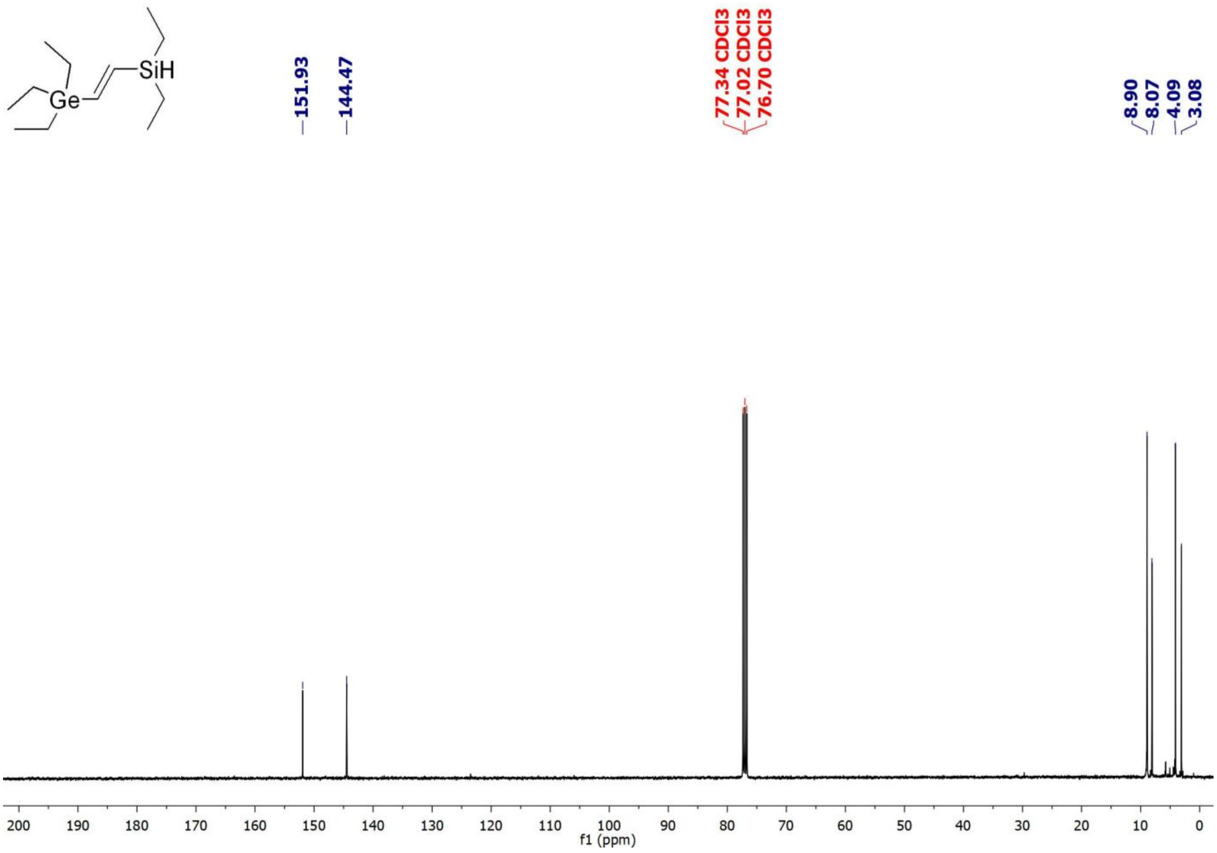

Figure S65. ^{13}C NMR (101 MHz, Chloroform-d) of diethyl(2-(triethylgermyl)vinyl)silane **(5ai')**

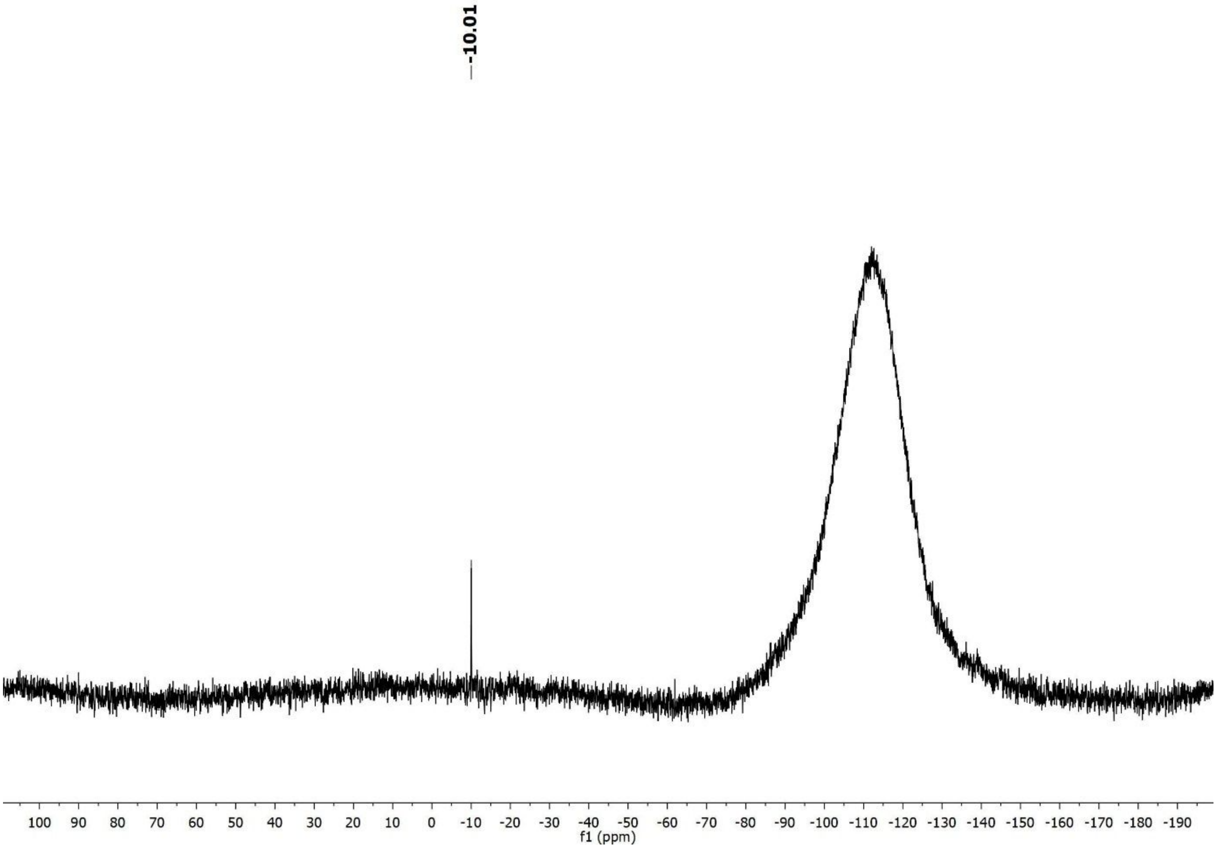

Figure S66. ^{29}Si NMR (79 MHz, Chloroform-d) of diethyl(2-(triethylgermyl)vinyl)silane **(5ai')**

(2-(diphenyl(2-(triethylgermyl)vinyl)silyl)ethyl)trimethylsilane (6a)

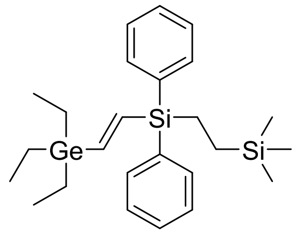

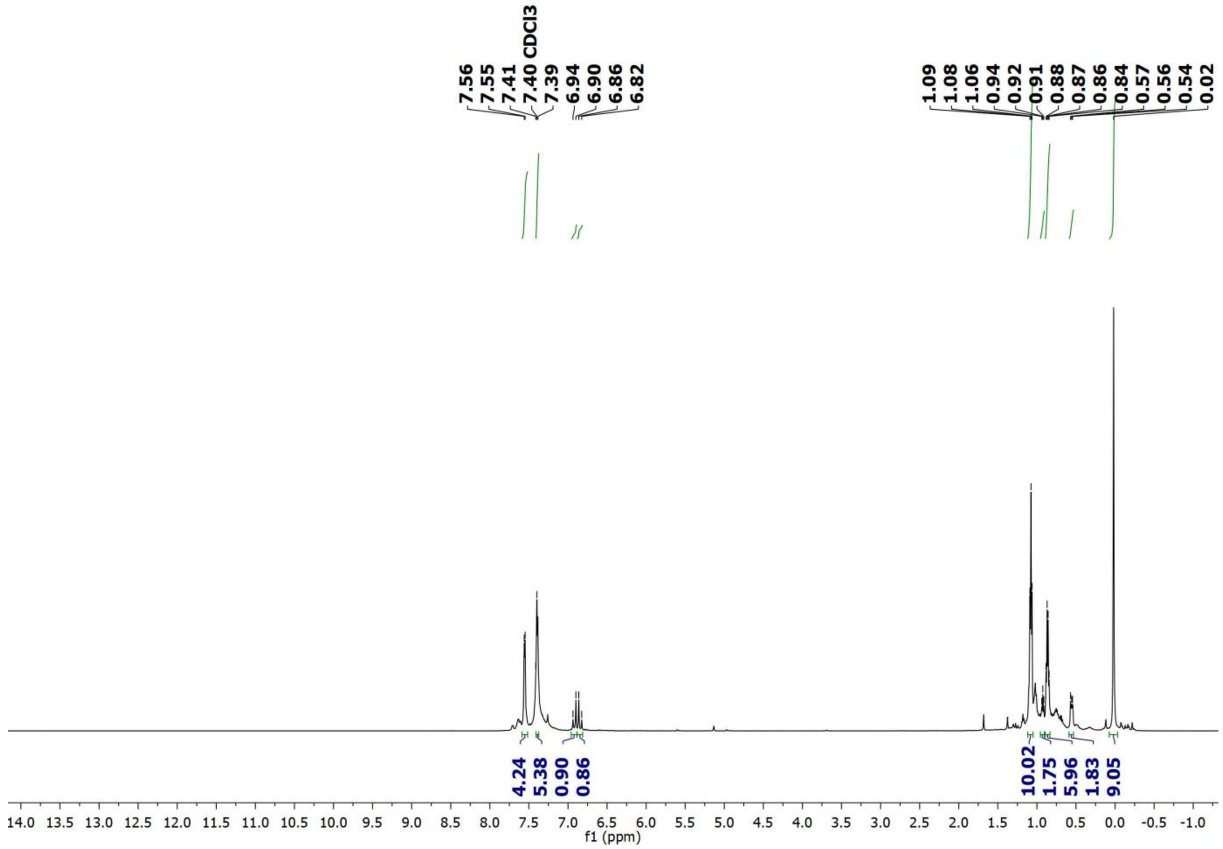

Figure S67. ^{1}H NMR (600 MHz, Chloroform-d) of (2-(diphenyl(2-(triethylgermyl)vinyl)silyl)ethyl)-trimethylsilane **(6a)**

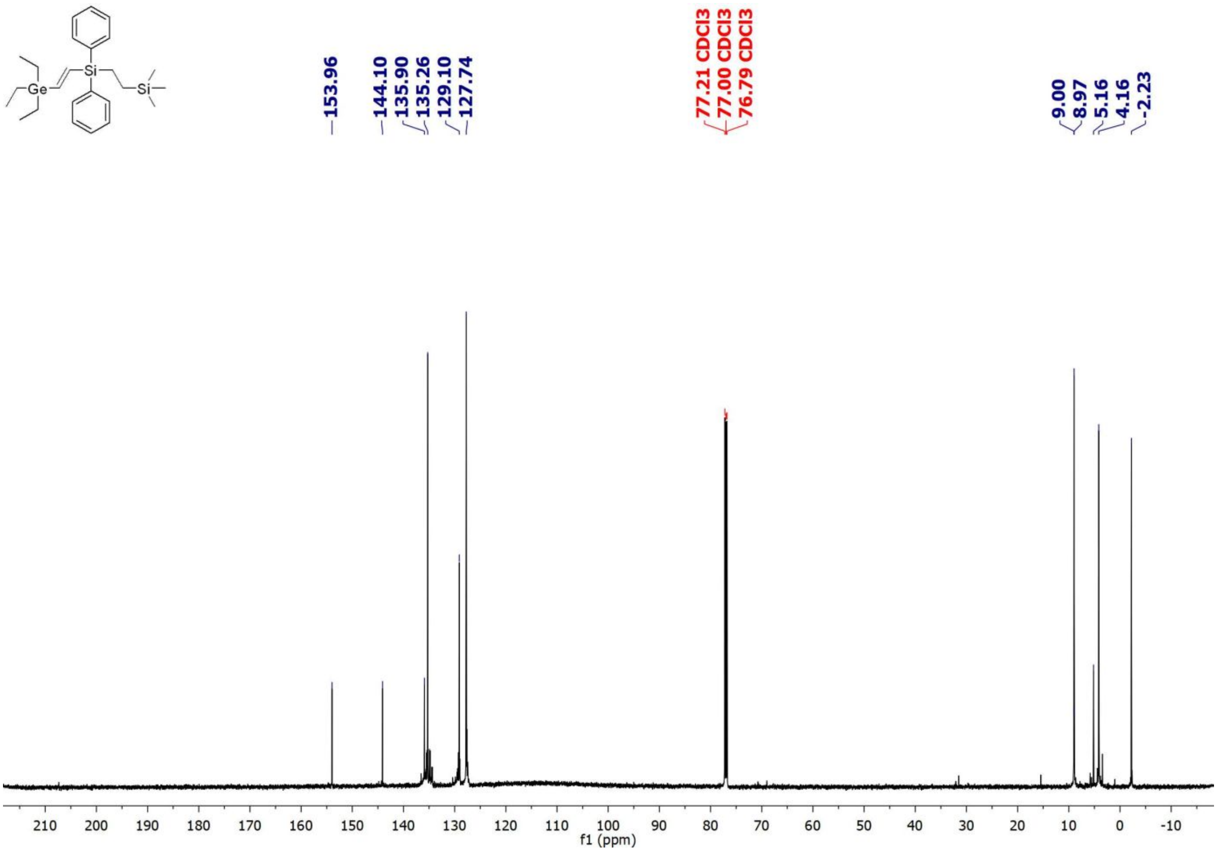

Figure S68. ^{13}C NMR (151 MHz, Chloroform-d) of (2-(diphenyl(2-(triethylgermyl)vinyl)silyl)ethyl)-trimethylsilane **(6a)**

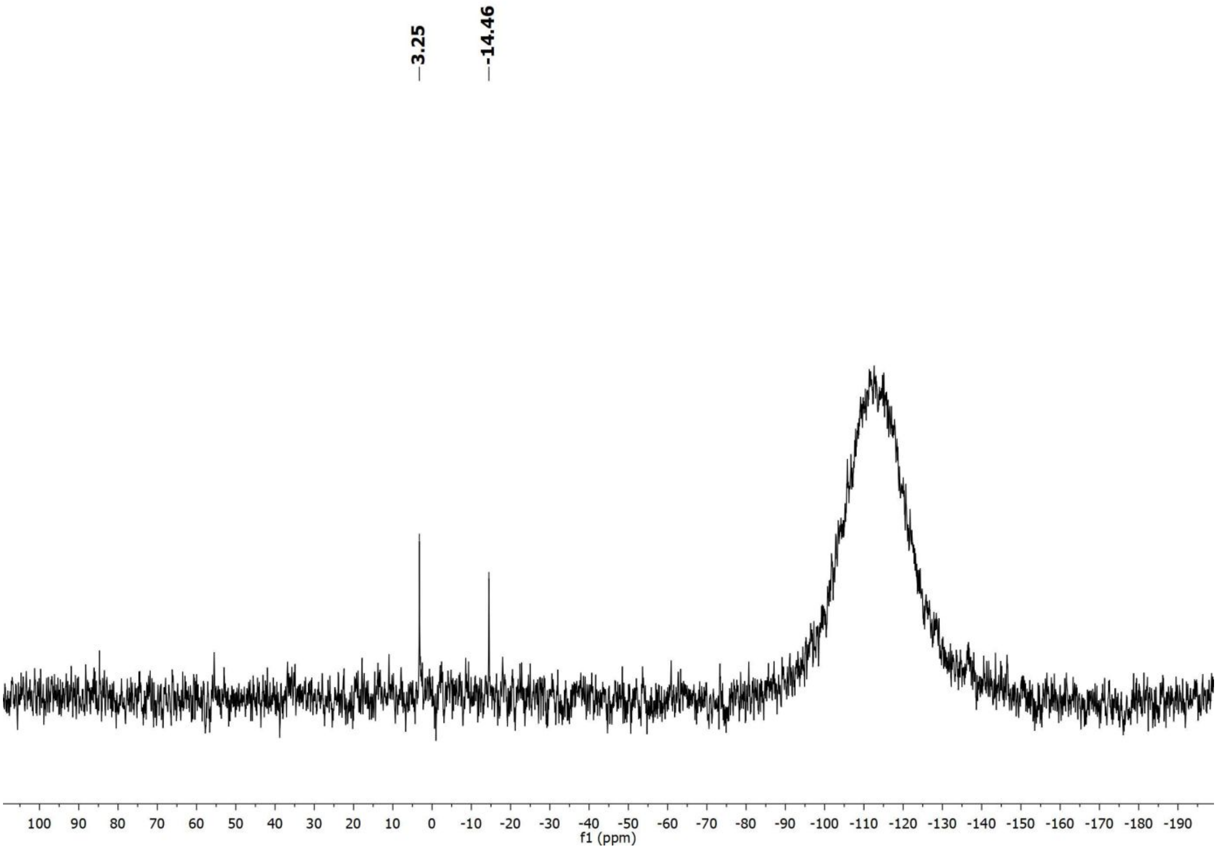

Figure S69. ^{29}Si NMR (79 MHz, Chloroform-d) of (2-(diphenyl(2-(triethylgermyl)vinyl)silyl)ethyl)-trimethylsilane **(6a)**

(2-isobutoxyethyl)diphenyl((triisopropylgermyl)ethynyl)silane (6b)

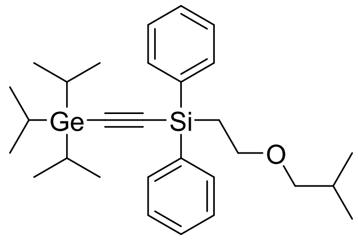

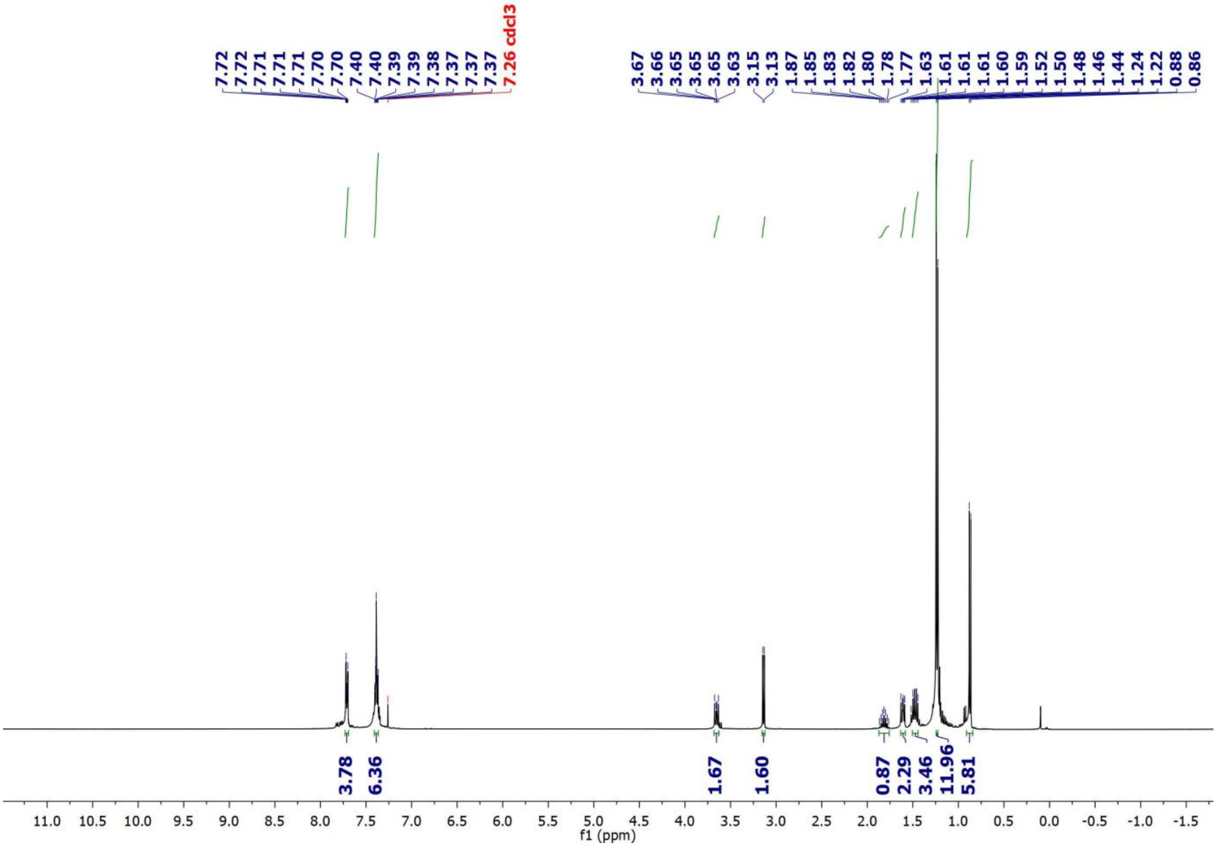

Figure S70. ^{1}H NMR (401 MHz, Chloroform-d) of (2-isobutoxyethyl)diphenyl((triisopropylgermyl)-ethynyl)silane **(6b)**

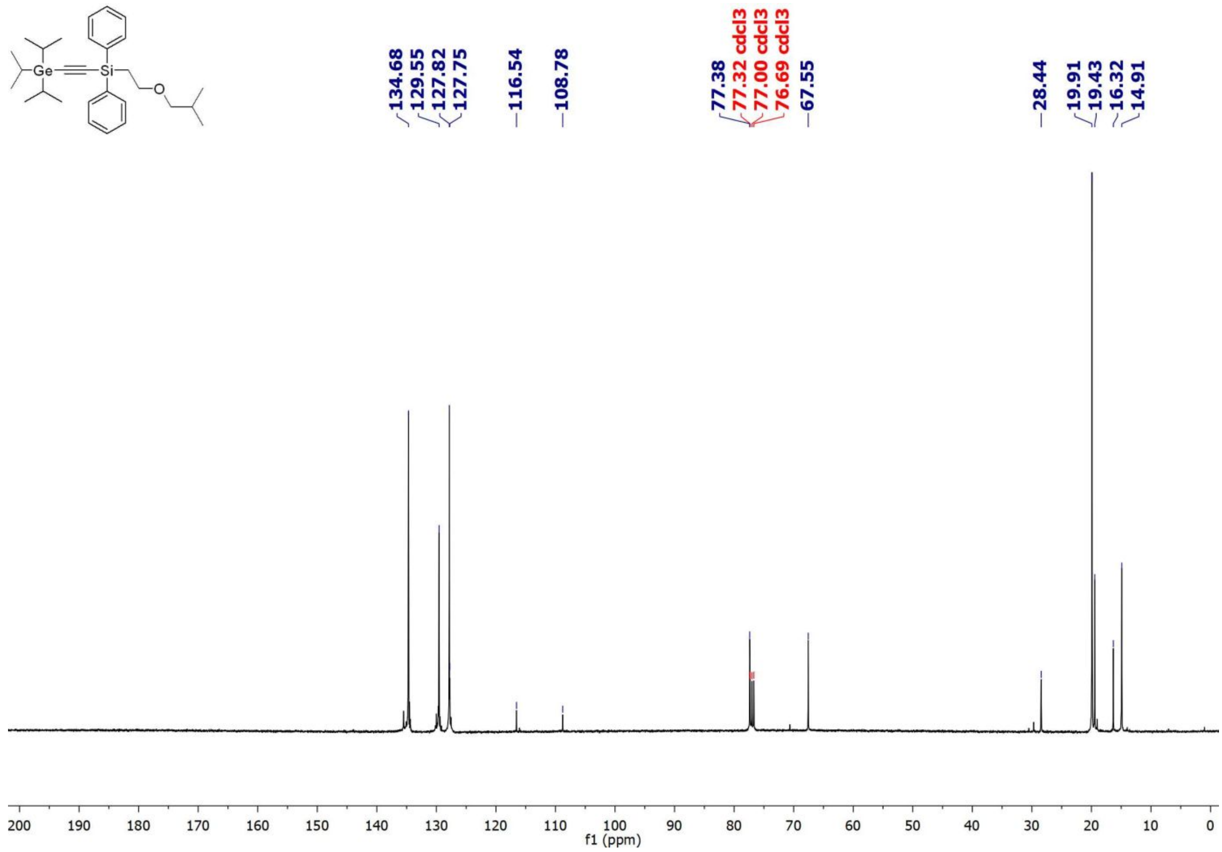

Figure S71. ^{13}C NMR (101 MHz, Chloroform-d) of (2-isobutoxyethyl)diphenyl((triisopropylgermyl)-ethynyl)silane **(6b)**

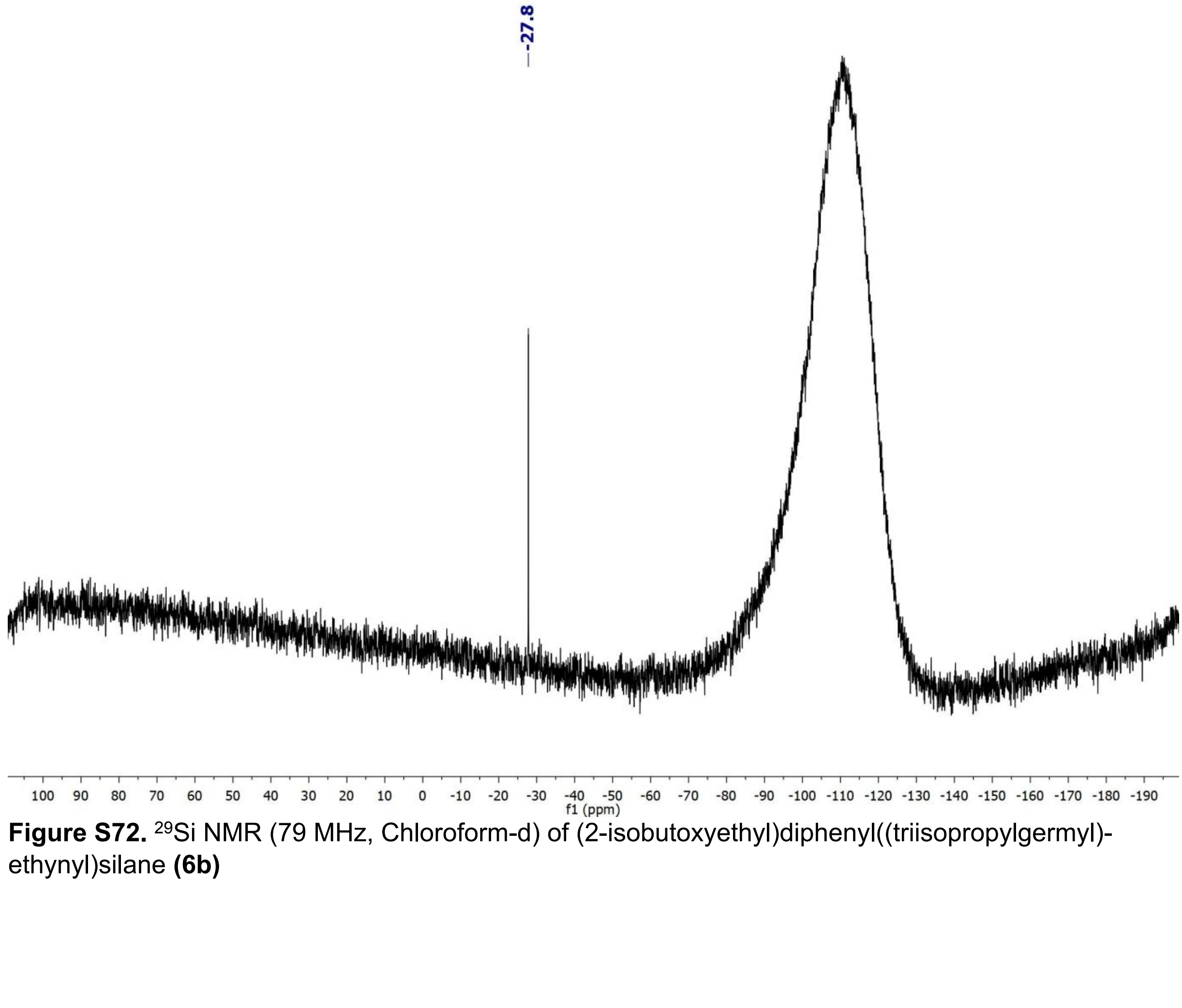

Figure S72. ^{29}Si NMR (79 MHz, Chloroform-d) of (2-isobutoxyethyl)diphenyl((triisopropylgermyl)-ethynyl)silane **(6b)**

(2-(dimethyl(phenyl)silyl)ethyl)diphenyl(2-(triethylgermyl)vinyl)silane (6c)

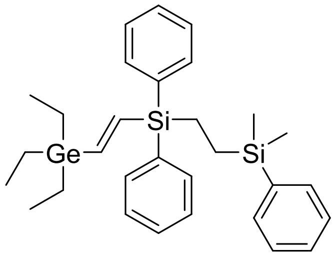

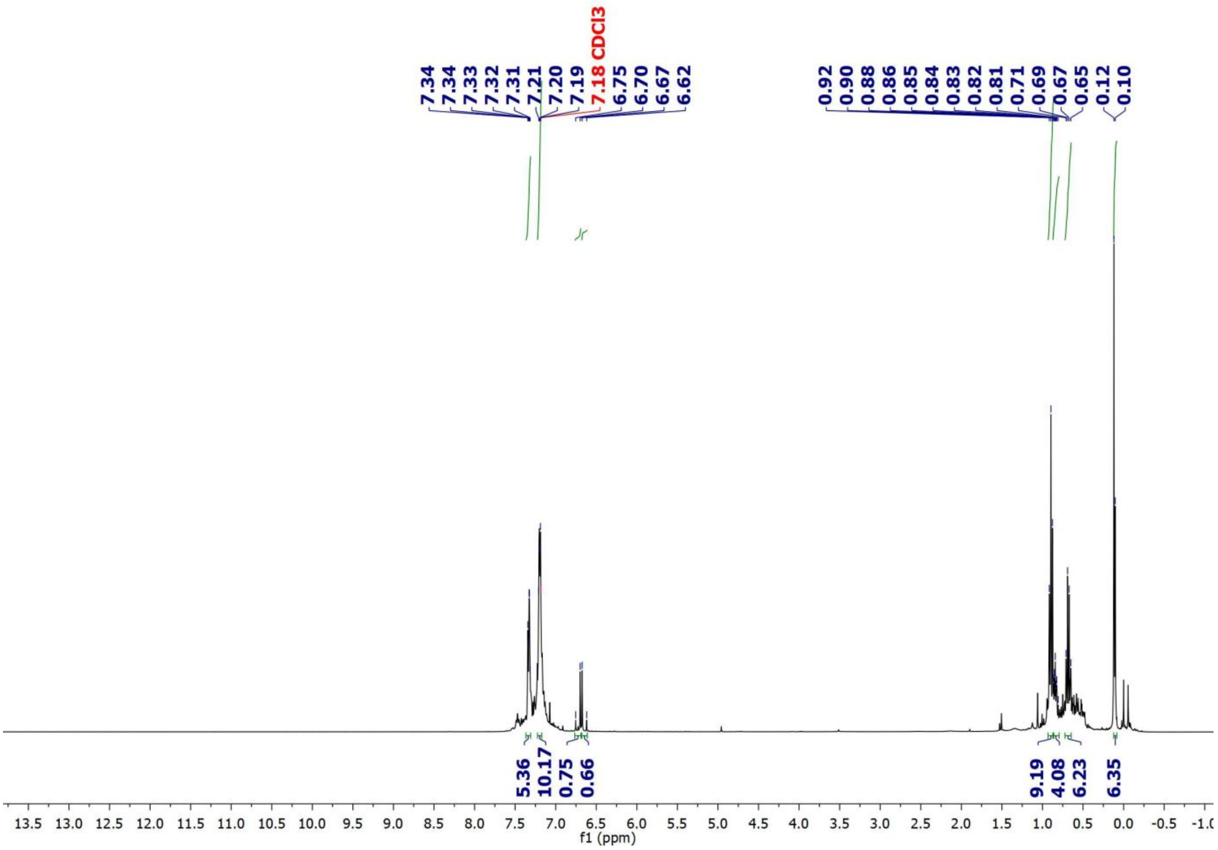

Figure S73. ^{1}H NMR (400 MHz, Chloroform-d) of (2-(dimethyl(phenyl)silyl)ethyl)diphenyl(2-(triethylgermyl)vinyl)silane **(6c)**

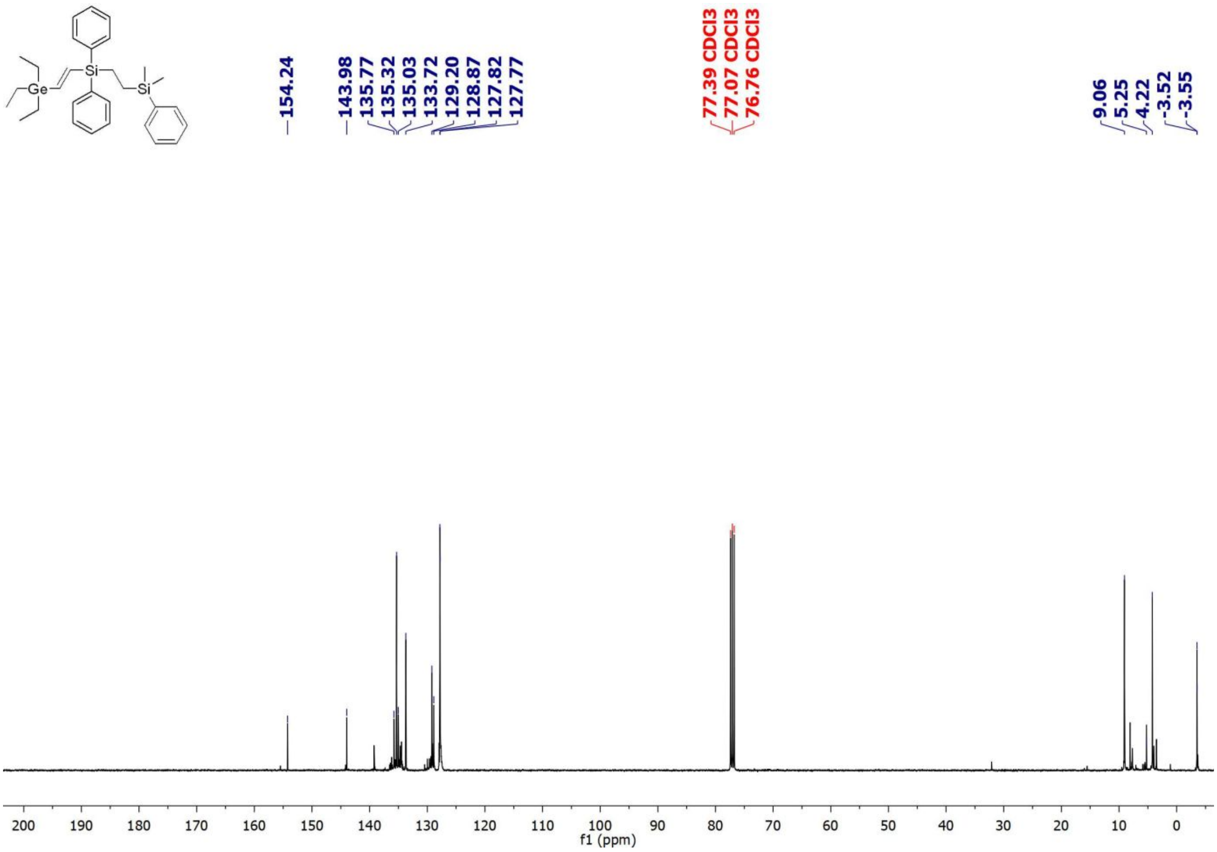

Figure S74. ^{13}C NMR (400 MHz, Chloroform-d) of (2-(dimethyl(phenyl)silyl)ethyl)diphenyl(2-(triethylgermyl)vinyl)silane **(6c)**

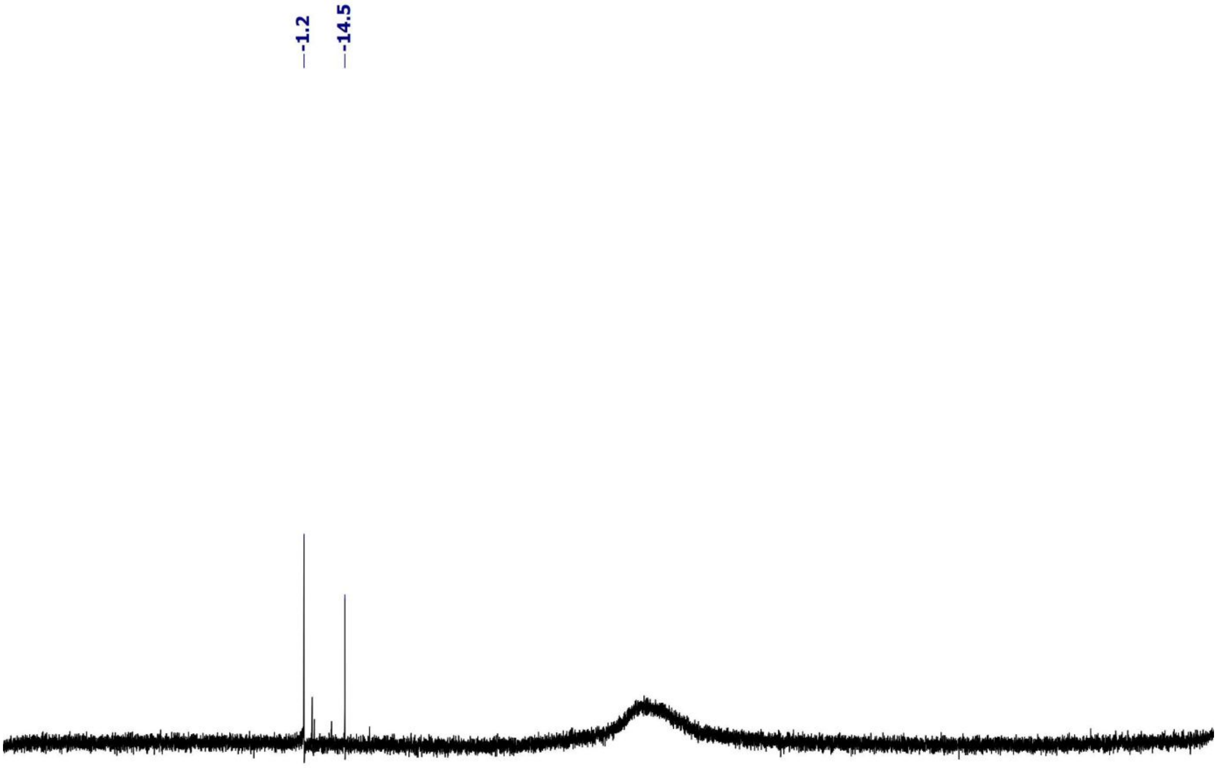

Figure S75. ^{29}Si NMR (400 MHz, Chloroform-d) of (2-(dimethyl(phenyl)silyl)ethyl)diphenyl(2-(triethylgermyl)vinyl)silane **(6c)**

MECHANISTIC STUDIES

Supplement 1

Conditions: 1 eq of **A**, 10 eq of **2a**, THF-d8, 40°C, 24 h

Triplet from formation [Co]-H: **^{1}H NMR** (400 MHz, THF-d_8) δ -9.89 (t, *J* = 43.0 Hz, 1H).

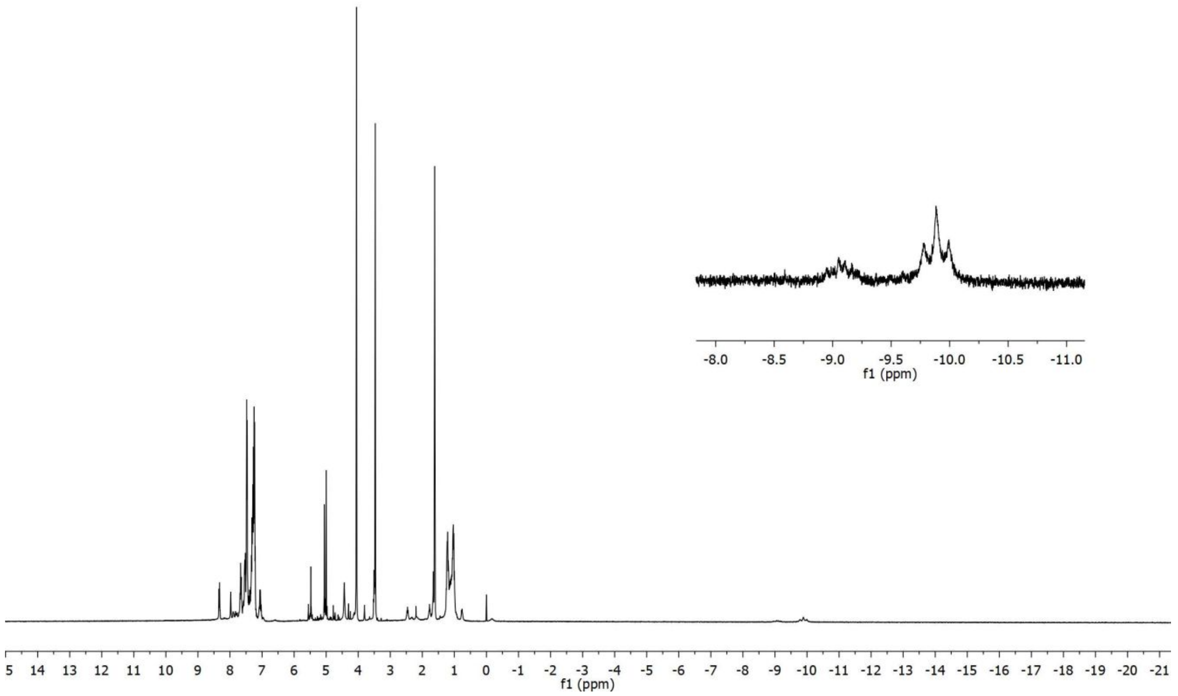

Figure S76. ^{1}H NMR (400 MHz, THF-d_8)

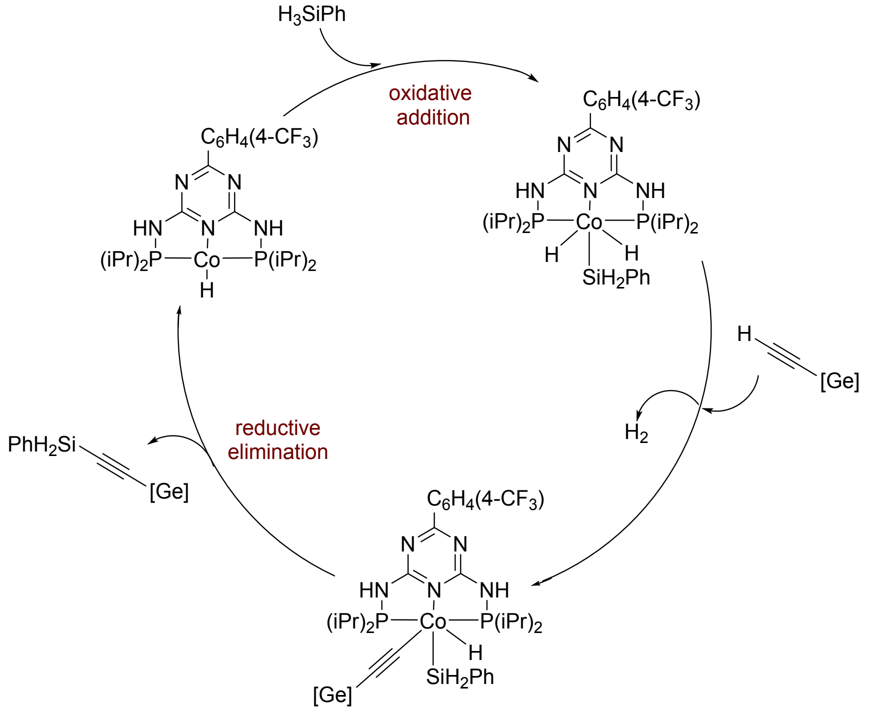

Figure S77. Plausible catalytic cycle.

Supplement 2

Conditions: 1 eq of **A**, 10 eq of **2h**, THF-d8, 40°C, 2 h

Triplets from formation of two [Co]-H species: **^{1}H NMR** (400 MHz, THF-d_8) δ -8.89 (t, *J* = 44.8 Hz, 1H), -9.63 (t, *J* = 44.7 Hz, 1H).

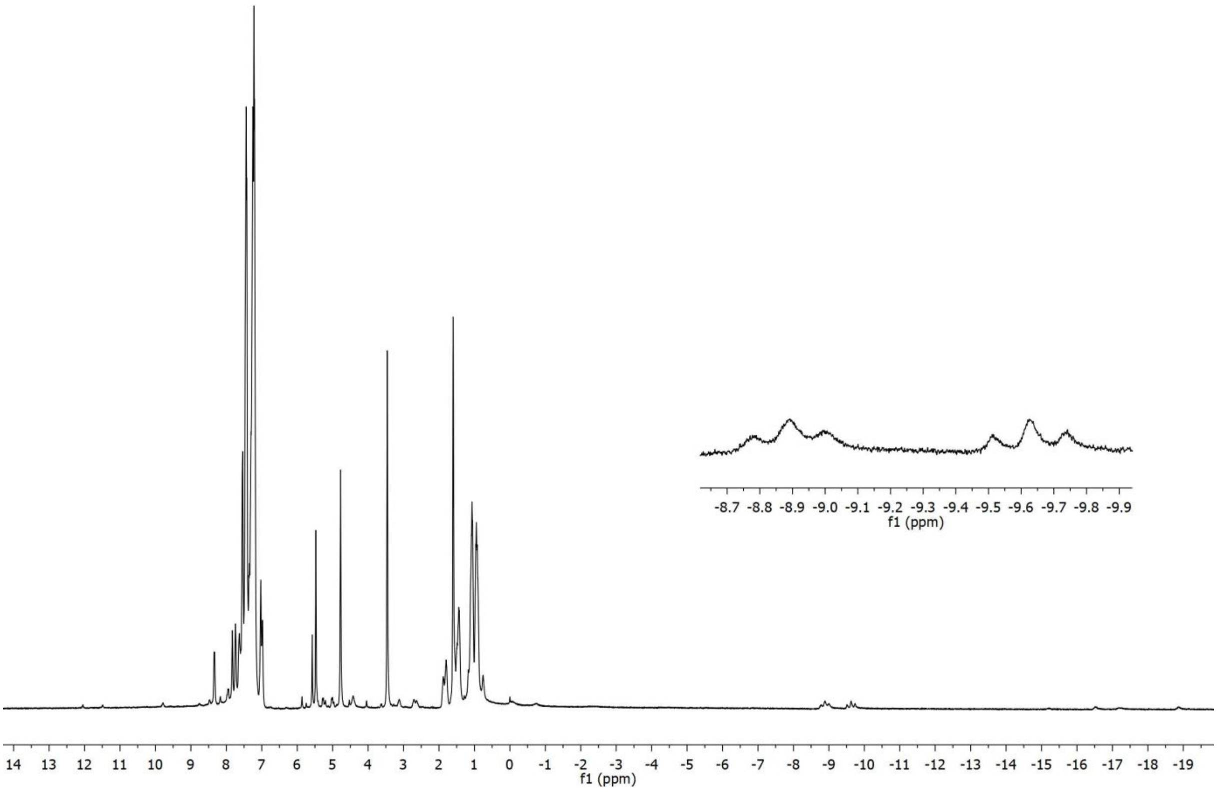

Figure S78. ^{1}H NMR (400 MHz, THF-d_8)

Supplement 3

Conditions: 1 eq of **A**, 2 eq of LiO^{t}Bu, 10 eq of **2h**, THF-d8, 40°C, 2 h

Triplet from formation of one[Co]-H species: **^{1}H NMR** (400 MHz, THF-d_8) δ -9.39 (t, *J* = 44.8 Hz, 1H)

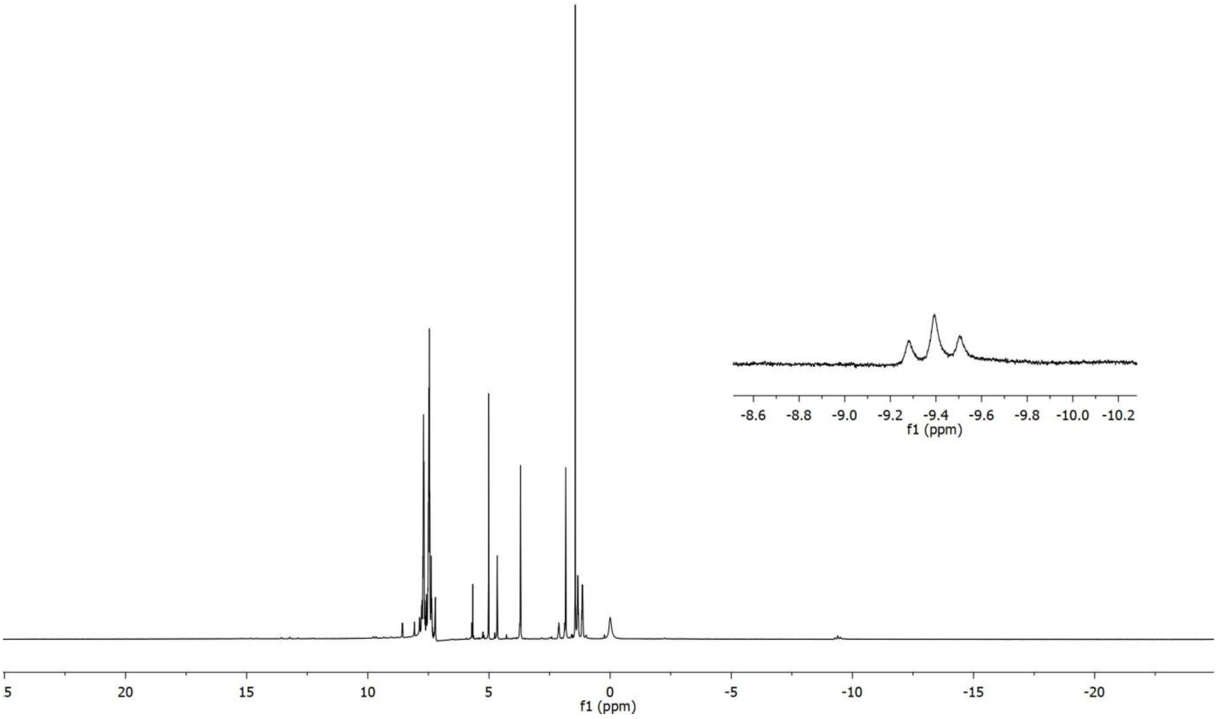

Figure S79. ^{1}H NMR (400 MHz, THF-d_8)
